# Chiral bifunctional sulfide-catalyzed enantioselective bromolactonizations of α- and β-substituted 5-hexenoic acids

**DOI:** 10.3762/bjoc.20.158

**Published:** 2024-07-30

**Authors:** Sao Sumida, Ken Okuno, Taiki Mori, Yasuaki Furuya, Seiji Shirakawa

**Affiliations:** 1 Institute of Integrated Science and Technology, Nagasaki University, 1-14 Bunkyo-machi, Nagasaki 852-8521, Japan

**Keywords:** asymmetric catalysis, enantioselectivity, halogenation, lactones, organocatalysis

## Abstract

Enantioselective halolactonizations of sterically less hindered alkenoic acid substrates without substituents on the carbon–carbon double bond have remained a formidable challenge. To address this limitation, we report herein the asymmetric bromolactonization of 5-hexenoic acid derivatives catalyzed by a BINOL-derived chiral bifunctional sulfide.

## Introduction

**Scheme 1:**
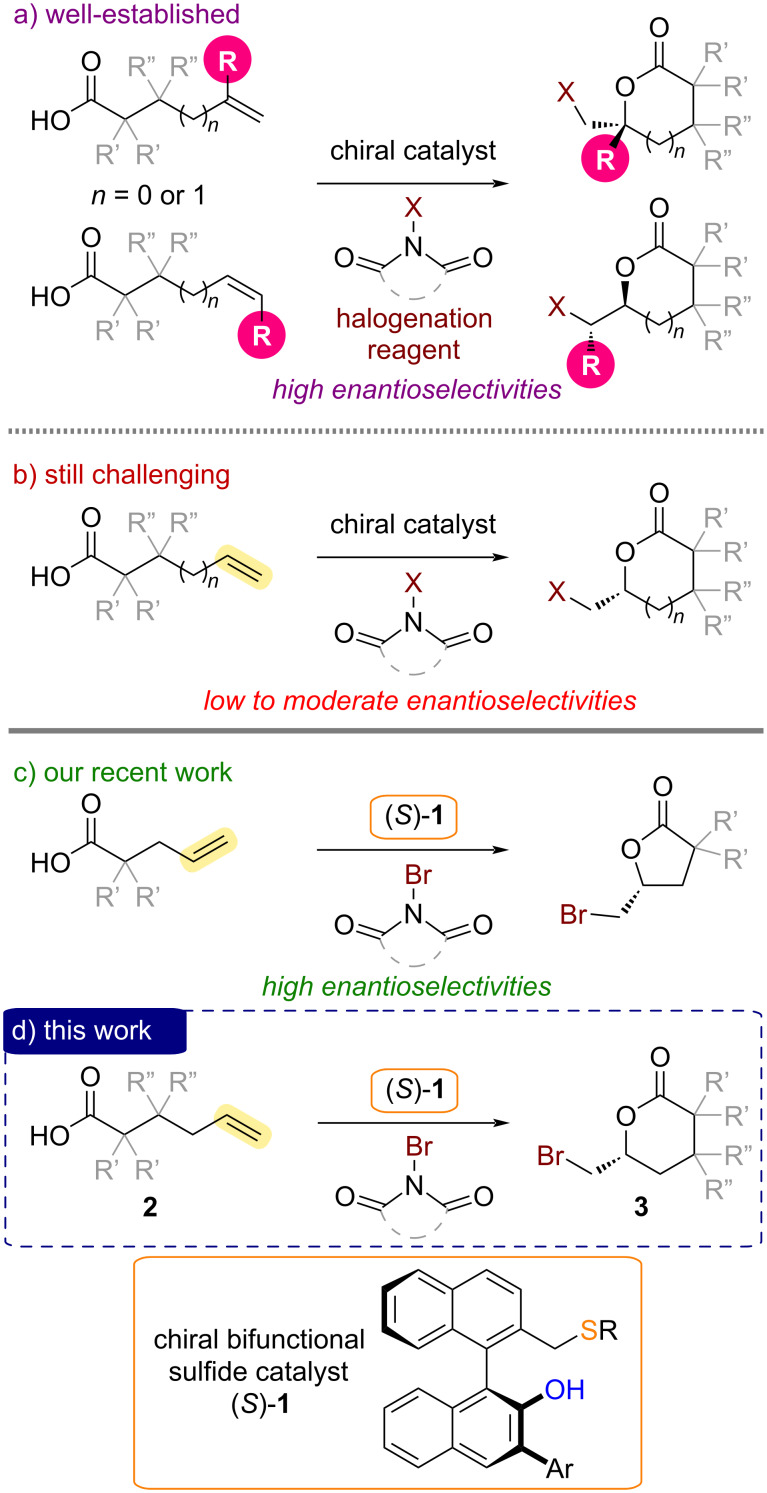
Catalytic asymmetric halolactonizations of alkenoic acids.

Catalytic asymmetric halolactonizations of alkenoic acids are powerful methods for the preparation of important chiral lactones in enantioenriched forms [1–11]. A wide variety of chiral catalysts have been applied to asymmetric halolactonizations, especially for the synthesis of chiral γ-butyrolactones and δ-valerolactones via the reaction of 4-pentenoic acid and 5-hexenoic acid derivatives (Scheme 1). Notably, however, substituents on the carbon–carbon double bond of alkenoic acid substrates are generally required to achieve highly enantioselective halolactonizations (Scheme 1a) [1–22]. Enantioselective halolactonizations of sterically less hindered alkenoic acid substrates without substituents on the carbon–carbon double bond have remained a formidable challenge in the field of catalytic asymmetric synthesis (Scheme 1b) [23–25]. To address this limitation, we have investigated the use of BINOL-derived chiral bifunctional sulfide catalysts, which were developed by our group [10], in asymmetric bromolactonizations of α-substituted 4-pentenoic acids without additional substituents on the carbon–carbon double bond (Scheme 1c) [26–27]. Chiral α-substituted γ-butyrolactone products as important building blocks for pharmaceutical development were obtained in a highly enantioselective manner in our catalytic system using bifunctional sulfide (*S*)-**1** [26–31]. To further demonstrate the utility of our chiral bifunctional sulfide catalysts in challenging halolactonizations, we next turned our attention to the asymmetric bromolactonizations of 5-hexenoic acid derivatives **2** for the synthesis of optically active δ-valerolactones **3** (Scheme 1d). Herein, we report our additional efforts to overcome limitations in catalytic asymmetric halolactonizations.

## Results and Discussion

**Scheme 2:**
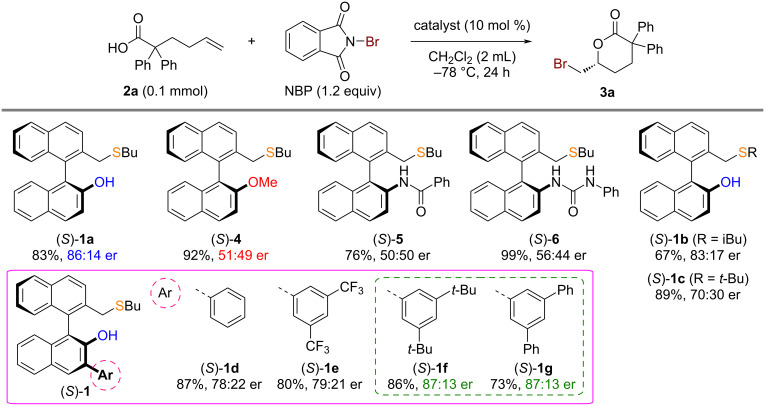
Effects of chiral sulfide catalysts.

α,α-Diphenyl-5-hexenoic acid (**2a**) was selected as a model substrate to evaluate the performance of our BINOL-derived chiral bifunctional sulfide catalysts in the asymmetric bromolactonization of 5-hexenoic acid derivatives without additional substituents on the carbon–carbon double bond (Scheme 2). The catalytic asymmetric bromolactonization of model substrate **2a** with *N*-bromophthalimide (NBP) was conducted at −78 °C for 24 hours using chiral bifunctional sulfide (*S*)-**1a** (10 mol %) bearing a hydroxy group. This reaction yielded the desired δ-valerolactone product **3a** with good yield and enantioselectivity [83% yield, 86:14 enantiomeric ratio (er)]. We further tested the reaction of **2a** with a hydroxy-protected sulfide catalyst (*S*)-**4** under the same conditions to evaluate the importance of the bifunctional design of the hydroxy-type chiral sulfide catalyst (*S*)-**1a**. As expected, the use of the hydroxy-protected catalyst (*S*)-**4** produced **3a** with significantly lower enantioselectivity (51:49 er). This outcome clearly underscores the crucial role of the bifunctional design in chiral sulfide catalysts (*S*)-**1** for enantioselective bromolactonizations of 5-hexenoic acid derivatives without additional substituents on the carbon–carbon double bond [26–31]. We also investigated the effects of other types of BINOL-derived chiral bifunctional sulfide catalysts. Asymmetric bromolactonizations of model substrate **2a** with amide- and urea-type chiral bifunctional sulfides (*S*)-**5** and **6**, known to be effective for other asymmetric halocyclizations [32–35], resulted in δ-valerolactone product **3a** with low enantioselectivities (50:50 and 56:44 er, respectively). These findings led us to further optimize the hydroxy-type chiral sulfide catalysts of type (*S*)-**1**. Substituting an alkyl group on sulfur of catalyst (*S*)-**1** with isobutyl and *tert*-butyl [(*S*)-**1b** and **1c**, respectively] decreased enantioselectivity compared with the *n*-butyl group-substituted catalyst [(*S*)-**1a**]. Next, the effects of aryl substituents at the 3-position of a binaphthyl unit on the hydroxy-type chiral sulfide catalysts [(*S*)-**1d**–**g**] were investigated. Fortunately, the attachments of 3,5-di-*tert*-butylphenyl and 3,5-diphenylphenyl groups [(*S*)-**1f** and **1g**, respectively] slightly improve the enantioselectivity (87:13 er).

**Scheme 3:**
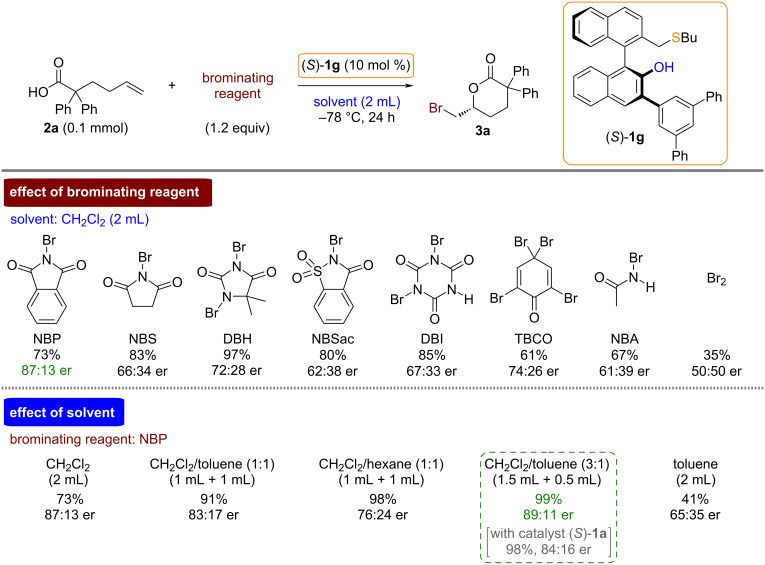
Effects of brominating reagents and solvents.

We next examined the effects of brominating reagents in the asymmetric bromolactonization of **2a** under the influence of chiral bifunctional sulfide catalyst (*S*)-**1g** in dichloromethane (Scheme 3). Among the examined brominating reagents, NBP provided higher enantioselectivity for the bromolactonization product **3a**. It should be noted that the asymmetric reaction using bromine (Br_2_) as a brominating reagent gave product **3a** in a racemic form. Additionally, iodolactonization of **2a** using *N*-iodosuccinimide in the presence of catalyst (*S*)-**1g** was performed. The reaction in dichloromethane, however, provided the corresponding iodolactonization product in racemic form with a good yield (80% yield, 50:50 er) [36].

To improve the enantioselectivity in the asymmetric bromolactonization of **2a** using NBP and catalyst (*S*)-**1g**, the optimization of reaction solvents was also performed. Based on our recent studies of chiral bifunctional sulfide-catalyzed bromolactonizations [26–31], mixed solvent systems were investigated. Among the examined solvent systems, a dichloromethane/toluene mixed solvent (3:1 ration) showed the best enantioselectivity (89:11 er).

**Scheme 4:**
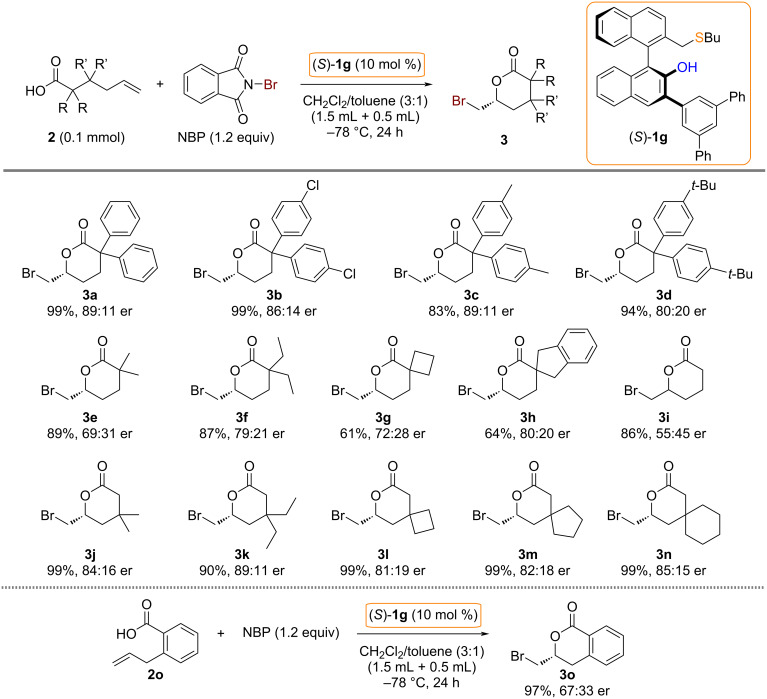
Substrate scope.

With the optimal catalyst (*S*)-**1g** and reaction conditions in hand, we investigated the generality of catalytic asymmetric bromolactonizations of 5-hexenoic acids **2** (Scheme 4). Asymmetric bromolactonizations with α,α-diaryl type 5-hexenoic acids **2a**–**d** provided δ-valerolactone products **3a**–**d** in high yields with good levels of enantioselectivity. The reactions of α,α-dialkyl-5-hexenoic acids **2e** and **2f** gave the corresponding bromolactonization products **3e** and **3f** with moderate enantioselectivities. The present catalytic method could also be applied to the asymmetric synthesis of spirolactones [37–39]. For example, α-spiro-δ-lactone products **3g** and **3h** were obtained with moderate to good levels of enantioselectivity. Unfortunately, the reaction with a simple 5-hexenoic acid **2i** gave a δ-valerolactone **3i** in low enantioselectivity. To expand the substrate scope of chiral bifunctional sulfide-catalyzed asymmetric bromolactonizations of 5-hexenoic acids **2**, β-substituted substrates **2j**–**n** were submitted to the present catalytic system. As a result of these asymmetric reactions, β,β-dialkyl-δ-valerolactones **3j**–**k** and β-spiro-δ-lactones **3l**–**n** were obtained in good levels of enantioselectivity. The reaction of 2-allylbenzoic acid **2o** as a related substrate was also examined to give a dihydroisocoumarin product **3o** in high yield with moderate enantioselectivity. The absolute stereochemistry of the bromolactonization product **3o** was confirmed by comparison with reported data [24].

**Scheme 5:**
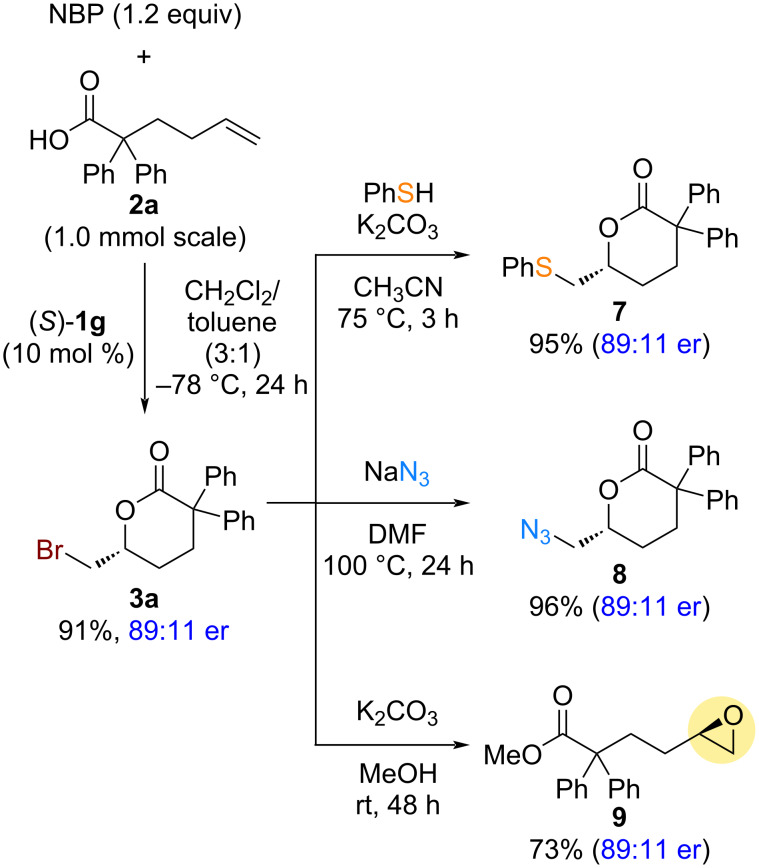
Larger-scale synthesis and transformations of bromolactonization product **3a**.

The transformations of the optically active bromolactonization product **3a** were explored to demonstrate the broader applicability of the current synthetic method (Scheme 5). Asymmetric bromolactonization of α,α-diphenyl-5-hexenoic acid (**2a**), using a chiral bifunctional sulfide catalyst (*S*)-**1g**, was scaled up to a 1.0 mmol scale to obtain the optically active bromolactonization product **3a** for further transformations. Comparable yield and enantioselectivity were observed relative to those of the smaller-scale reaction (0.1 mmol scale, Scheme 4). The bromomethyl group in **3a** readily undergoes nucleophilic substitution reactions, leading to the formation of optically active δ-valerolactones **7** and **8**, which are functionalized with sulfur and nitrogen, in high yields. Additionally, optically active δ-valerolactone **3a** was converted to optically active epoxy-ester **9** upon treatment with potassium carbonate in methanol. Notably, the transformed products were obtained without any loss of optical purity.

## Conclusion

In summary, our BINOL-derived chiral bifunctional sulfide-catalyzed enantioselective halocyclization technology was successfully applied to the catalytic asymmetric bromolactonization of α- and β-substituted 5-hexenoic acids. The target optically active δ-valerolactone products were obtained in moderate to good levels of enantioselectivity. The utility of the prepared optically active bromolactonization products was demonstrated in the transformations to functionalized δ-valerolactones and epoxy-esters. These transformations proceeded with no loss of optical purity. This report provides a valuable example of catalytic enantioselective halolactonization of 5-hexenoic acid derivatives without extra substituents on the carbon–carbon double bond.

## Supporting Information

File 1 Experimental procedures, characterization data, copies of NMR spectra, and copies of HPLC charts.

## Data Availability

All data that supports the findings of this study is available in the published article and/or the supporting information to this article.
