# Supplementary material for: Chiral bifunctional sulfide-catalyzed enantioselective bromolactonizations of α- and β-substituted 5-hexenoic acids
Source: Beilstein J Org Chem. 2024 Jul 30;20:1794–9. doi: 10.3762/bjoc.20.158 (PMC11301038; doi:10.3762/bjoc.20.158)
Supplement: File 1 — Experimental procedures, characterization data, copies of NMR spectra, and copies of HPLC charts. [file Beilstein_J_Org_Chem-20-1794-s001.pdf]

BEILSTEIN JOURNAL OF ORGANIC CHEMISTRY

# Supporting Information

for

# Chiral bifunctional sulfide-catalyzed enantioselective bromolactonizations of α- and β-substituted 5-hexenoic acids

Sao Sumida, Ken Okuno, Taiki Mori, Yasuaki Furuya and Seiji Shirakawa

*Beilstein J. Org. Chem.* **2024,** *20,* 1794–1799. doi:10.3762/bjoc.20.158

## Experimental procedures, characterization data, copies of NMR spectra, and copies of HPLC charts

License and Terms: This is a supporting information file under the terms of the Creative Commons Attribution License (https://creativecommons.org/licenses/by/4.0). Please note that the reuse, redistribution and reproduction in particular requires that the author(s) and source are credited and that individual graphics may be subject to special legal provisions.
The license is subject to the *Beilstein Journal of Organic Chemistry* terms and conditions: (https://www.beilstein-journals.org/bjoc/terms)

# Table of Contents

**General information** S-2

**Experimental section** S-3

**Synthesis of catalysts.** S-3

**Preparation of substrates.** S-3

**General procedure for asymmetric bromolactonizations (Scheme 4).** S-4

**Asymmetric bromolactonization of 2a on a larger scale (Scheme 5).** S-10

**Transformations of 3a (Scheme 5).** S-10

**Determination of the absolute configuration of products.** S-12

**References** S-13

**NMR Charts** S-14

**HPLC Charts** S-40

## General information

$^{1}H$ and $^{13}C$ NMR spectra were measured on a JEOL JNM-ECZ 400R NMR instrument (400 MHz for $^{1}H$ NMR, 100 MHz for $^{13}C$ NMR). Tetramethylsilane (TMS) served as the internal standard (0 ppm) for $^{1}H$ NMR, and $CDCl_3$ served as the internal standard (77.0 ppm) for $^{13}C$ NMR. The following abbreviations were used to express the multiplicities: s = singlet; d = doublet; t = triplet; q = quartet; m = multiplet. High-resolution mass spectra (HRMS) were measured on a JEOL JMS-700N. Infrared spectra (IR) were measured on a JASCO FT/IR-4200 spectrometer. Optical rotations were measured on a JASCO P-2100 polarimeter. High-performance liquid chromatography (HPLC) was performed on Shimadzu LC-20AT and SPD-20A instruments using Daicel Chiralpak AS-3, IB-3, IC-3, IE-3, IG-3 columns (4.6 mm × 250 mm). All reactions were monitored by thin-layer chromatography using Merck precoated TLC plates (silica gel 60GF-254, 0.25 mm), with visualization by the use of UV lamp (254 nm) or dyes. The products were purified by flash column chromatography on silica gel.

## Experimental section

### Synthesis of catalysts.

BINOL-derived chiral sulfide catalysts (*S*)-**1a**,[S1] (*S*)-**1b**,[S2] (*S*)-**1c**,[S3] (*S*)-**1d**,[S4] (*S*)-**1e**,[S2] (*S*)-**1f**,[S2] (*S*)-**1g**,[S4] (*S*)-**4**,[S1] (*S*)-**5**,[S5] and (*S*)-**6**[S1] were prepared according to the literature method.

### Preparation of substrates.

Substrates **2a**, **2e**, **2f**, **2g**, **2h**, **2i**, **2j**, and **2o** are known and commercially available compounds. Substrates **2b**,[S6] **2c**,[S6] **2d**,[S6] **2k**,[S7] **2l**,[S7] **2m**[S7] and **2n**[S7] were prepared according to the literature procedures.

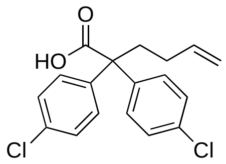


**2b**: $^1$H NMR (400 MHz, $CDCl_3$) δ 7.29 (d, *J* = 8.8 Hz, 4H), 7.22 (d, *J* = 8.8 Hz, 4H), 5.79–5.69 (m, 1H), 4.99–4.93 (m, 2H), 2.42–2.28 (m, 2H), 1.82–1.76 (m, 2H); $^{13}$C NMR (100 MHz, $CDCl_3$) δ 179.9, 140.3, 137.5, 133.3, 130.3, 128.3, 115.1, 59.2, 36.9, 29.4; IR (neat): 3076, 2977, 2958, 2936, 1698, 1492, 1095, 1013, 905, 820, 729 cm$^{-1}$; HRMS (EI) calcd for $C_{18}H_{16}Cl_2O_2$: 334.0527 ([M]$^+$), found 334.0527.

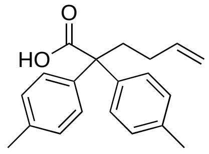


**2c**: $^1$H NMR (400 MHz, $CDCl_3$) δ 7.18 (dt, *J* = 8.4, 2.0 Hz, 4H), 7.10 (d, *J* = 8.0 Hz, 4H), 5.80–5.70 (m, 1H), 4.98–4.88 (m, 2H), 2.42–2.38 (m, 2H), 2.33 (s, 6H), 1.83–1.77 (m, 2H); $^{13}$C NMR (100 MHz, $CDCl_3$) δ 181.2, 139.3, 138.3, 136.5, 128.8, 128.6, 114.5, 59.4, 37.0, 29.6, 20.9; IR (neat): 3064, 2923, 2871, 1696, 1510, 1260, 907, 811, 732 cm$^{-1}$; HRMS (EI) calcd for $C_{20}H_{22}O_2$: 294.1620 ([M]$^+$), found 294.1620.

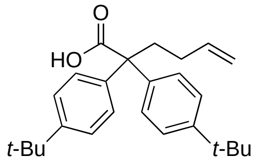


**2d**: $^1$H NMR (400 MHz, $CDCl_3$) δ 7.31 (dt, *J* = 8.8, 2.0 Hz, 4H), 7.22 (dt, *J* = 8.4, 2.0 Hz, 4H), 5.82–5.72 (m, 1H), 5.00–4.89 (m, 2H), 2.44–2.40 (m, 2H), 1.85–1.80 (m, 2H), 1.31 (s, 18H); $^{13}$C NMR (100 MHz, $CDCl_3$) δ 181.0, 149.6, 139.0, 138.4, 128.6, 124.8, 114.4, 59.3, 37.0, 34.4, 31.3, 29.5; IR (neat): 3076, 2962, 2904, 2869, 1697, 1269, 908,

826, 733 cm$^{-1}$; HRMS (FAB) calcd for $C_{26}H_{35}O_2$: 379.2637 ([M+H]$^+$), found 379.2638.

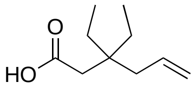

**2k**: $^1$H NMR (400 MHz, $CDCl_3$) δ 5.86–5.75 (m, 1H), 5.11–5.06 (m, 2H), 2.24 (s, 2H), 2.12 (d, $J$ = 7.5 Hz, 2H), 1.39 (q, $J$ = 7.5 Hz, 4H) , 0.84 (t, $J$ = 7.4 Hz, 6H); $^{13}$C NMR (100 MHz, $CDCl_3$) δ 179.2, 134.3, 117.8, 40.1, 39.9, 38.9, 28.2, 7.5; IR (neat): 3075, 2968, 2926, 2883, 1703, 1458, 1408, 1297, 1217, 914 cm$^{-1}$; HRMS (EI) calcd for $C_{10}H_{18}O_2$: 170.1307 ([M]$^+$), found 170.1307.

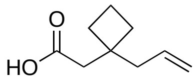

**2l**: $^1$H NMR (400 MHz, $CDCl_3$) δ 5.85–5.75 (m, 1H), 5.12–5.07 (m, 2H), 2.47 (s, 2H), 2.34 (dt, $J$ = 7.2, 1.2 Hz, 2H), 1.99–1.86 (m, 6H); $^{13}$C NMR (100 MHz, $CDCl_3$) δ 179.0, 134.5, 117.7, 42.91, 42.89, 39.7, 31.1, 15.2; IR (neat): 3077, 2978, 2933, 2859, 1702, 1408, 1274, 915 cm$^{-1}$; HRMS (EI) calcd for $C_9H_{14}O_2$: 154.0994 ([M]$^+$), found 154.0994.

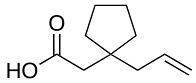

**2m**: $^1$H NMR (400 MHz, $CDCl_3$) δ 5.88–5.78 (m, 1H), 5.10–5.05 (m, 2H), 2.35 (s, 2H), 2.22 (d, $J$ = 7.5 Hz, 2H), 1.66–1.53 (m, 8H); $^{13}$C NMR (100 MHz, $CDCl_3$) δ 179.4, 135.2, 117.7, 44.3, 42.8, 42.5, 37.1, 24.2; IR (neat): 3076, 2952, 2869, 1702, 1442, 1407, 1291, 1224, 995, 914 cm$^{-1}$; HRMS (EI) calcd for $C_{10}H_{16}O_2$: 168.1150 ([M]$^+$), found 168.1150.

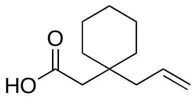

**2n**: $^1$H NMR (400 MHz, $CDCl_3$) δ 5.89–5.79 (m, 1H), 5.11–5.05 (m, 2H), 2.31 (s, 2H), 2.22 (d, $J$ = 7.5 Hz, 2H), 1.56–1.35 (m, 10H); $^{13}$C NMR (100 MHz, $CDCl_3$) δ 179.3, 134.2, 118.0, 41.7, 41.6, 36.1, 35.4, 26.0, 21.5; IR (neat): 3074, 2925, 2853, 1700, 1455, 1407, 1294, 1210, 913 cm$^{-1}$; HRMS (EI) calcd for $C_{11}H_{18}O_2$: 182.1307 ([M]$^+$), found 182.1307.

**General procedure for asymmetric bromolactonizations (Scheme 4).**

In a manner similar to our previous report,[S8] a solution of substrate **2** (0.10 mmol) and catalyst ($S$)-**1g** (6.0 mg, 0.010 mmol, 10 mol %) in $CH_2Cl_2$ (1.5 mL)-toluene (0.5 mL) was cooled to –78 °C. After stirring for 10 min at –78 °C, $N$-bromophthalimide (NBP) (27.1 mg, 0.12 mmol, 1.2 equiv) was added to the cooled reaction solution. The

reaction mixture was stirred for 24 h at –78 °C. After 24 h, the reaction mixture was quenched with saturated aqueous $Na_2SO_3$ (3 mL) at –78 °C and stirred for 10 min at –78 °C. The quenched reaction mixture was diluted with $CH_2Cl_2$ (2 mL) and $H_2O$ (2 mL), and the solution was warmed to room temperature. The organic materials were extracted with $CH_2Cl_2$ for three times (5 mL × 3). The combined extracts were dried over $Na_2SO_4$ and concentrated. The residue was purified by flash column chromatography on silica gel to give product **3**. The enantioselectivities of the isolated products **3** were determined by HPLC analysis on a chiral stationary phase.

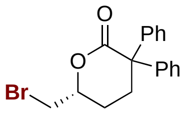

**3a**: $[\alpha]^{26}_D$ –23.4 (*c* = 1.0, $CHCl_3$, 89:11 er); HPLC analysis: Daicel Chiralpak IE-3, hexane/2-propanol = 2:1, flow rate = 0.5 mL/min, 254 nm; retention time: 22.2 min (major) and 25.6 min (minor). $^1$H NMR (400 MHz, $CDCl_3$) δ 7.38–7.24 (m, 8H), 7.18–7.15 (m, 2H), 4.54–4.47 (m, 1H), 3.49 (dd, *J* = 10.7, 4.3 Hz, 1H), 3.43 (dd, *J* = 10.6, 6.3 Hz, 1H), 2.81–2.74 (m, 1H), 2.69–2.63 (m, 1H), 2.11–2.03 (m, 1H), 1.94–1.85 (m, 1H); $^{13}$C NMR (100 MHz, $CDCl_3$) δ 172.4, 142.1, 141.1, 128.6, 128.4, 128.11, 128.09, 127.6, 127.3, 77.9, 56.8, 34.0, 31.6, 24.1; IR (neat): 3060, 3026, 2963, 2932, 1736, 1496, 1446, 1263, 1164, 1144, 1121, 1109, 1063, 751, 698 cm$^{-1}$; HRMS (EI) calcd for $C_{18}H_{17}BrO_2$: 344.0412 ($[M]^+$), found 344.0411.

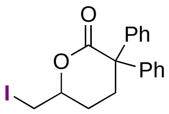

**Iodolactonization Product**: HPLC analysis: Daicel Chiralpak IE-3, hexane/2-propanol = 2:1, flow rate = 0.5 mL/min, 254 nm; retention time: 23.6 min and 27.7 min. $^1$H NMR (400 MHz, $CDCl_3$) δ 7.38–7.22 (m, 8H), 7.19–7.15 (m, 2H), 4.32–4.26 (m, 1H), 3.32 (ddd, *J* = 10.4, 4.2, 1.6 Hz, 1H), 3.24 (dd, *J* = 10.4, 6.7 Hz, 1H), 2.80–2.73 (m, 1H), 2.67–2.61 (m, 1H), 2.17–2.08 (m, 1H), 1.84–1.74 (m, 1H); $^{13}$C NMR (100 MHz, $CDCl_3$) δ 172.4, 142.0, 141.2, 128.5, 128.4, 128.14, 128.08, 127.5, 127.3, 78.1, 56.6, 31.8, 25.9, 7.7; IR (neat): 3059, 3031, 2958, 2937, 1733, 1496, 1446, 1258, 1156, 1137, 1121, 1108, 1052, 751, 698 cm$^{-1}$; HRMS (FAB) calcd for $C_{18}H_{18}IO_2$: 393.0351 ($[M+H]^+$), found 393.0351.

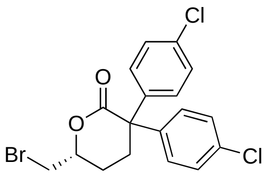

**3b**: $[\alpha]^{25}_D$ –10.4 (*c* = 1.0, $CHCl_3$, 86:14 er); HPLC analysis: Daicel Chiralpak IC-3, hexane/2-propanol = 2:1, flow rate = 0.5 mL/min, 254 nm; retention time: 19.9 min (minor) and 22.8 min (major). $^1$H NMR (400 MHz, $CDCl_3$) δ 7.35–7.29 (m, 4H), 7.16 (dt, *J* = 8.9,

2.2 Hz, 2H), 7.10 (dt, $J$ = 8.9, 2.2 Hz, 2H), 4.57–4.51 (m, 1H), 3.49 (dd, $J$ = 10.8, 4.4 Hz, 1H), 3.46 (dd, $J$ = 10.8, 5.8 Hz, 1H), 2.76–2.69 (m, 1H), 2.63–2.57 (m, 1H), 2.09–2.02 (m, 1H), 1.93–1.84 (m, 1H); $^{13}$C NMR (100 MHz, $CDCl_3$) δ 171.6, 140.3, 139.6, 133.8, 133.6, 129.49, 129.48, 128.8, 128.7, 78.3, 55.9, 33.9, 31.7, 24.0; IR (neat): 2963, 1732, 1492, 1248, 1164, 1145, 1128, 1095, 1012, 820, 734 cm$^{-1}$; HRMS (FAB) calcd for $C_{18}H_{16}BrCl_2O_2$: 412.9711 ([M+H]$^+$), found 412.9711.

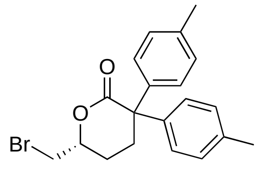

**3c**: $[\alpha]^{26}_{D}$ –20.7 ($c$ = 1.0, $CHCl_3$, 89:11 er); HPLC analysis: Daicel Chiralpak IE-3, hexane/2-propanol = 2:1, flow rate = 0.5 mL/min, 254 nm; retention time: 28.8 min (major) and 33.9 min (minor). $^1$H NMR (400 MHz, $CDCl_3$) δ 7.17–7.11 (m, 6H), 7.03 (dt, $J$ = 8.4, 2.0 Hz, 2H), 4.51–4.45 (m, 1H), 3.47 (dd, $J$ = 10.7, 4.3 Hz, 1H), 3.41 (dd, $J$ = 10.7, 6.2 Hz, 1H), 2.77–2.70 (m, 1H), 2.64–2.58 (m, 1H), 2.34 (s, 3H), 2.33 (s, 3H), 2.08–2.01 (m, 1H), 1.93–1.83 (m, 1H); $^{13}$C NMR (100 MHz, $CDCl_3$) δ 172.8, 139.3, 138.1, 137.3, 136.9, 129.3, 129.0, 127.93, 127.87, 77.7, 56.1, 34.1, 31.5, 24.2, 21.0, 20.9; IR (neat): 3024, 2961, 2922, 1733, 1512, 1249, 1160, 1143, 1134, 1104, 1062, 810, 731 cm$^{-1}$; HRMS (EI) calcd for $C_{20}H_{21}BrO_2$: 372.0725 ([M]$^+$), found 372.0719.

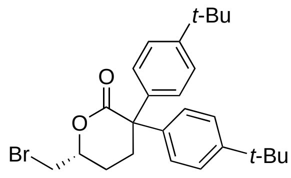

**3d**: $[\alpha]^{19}_{D}$ –13.2 ($c$ = 1.0, $CHCl_3$, 80:20 er); HPLC analysis: Daicel Chiralpak IE-3, hexane/2-propanol = 5:1, flow rate = 0.5 mL/min, 254 nm; retention time: 24.3 min (major) and 29.5 min (minor). $^1$H NMR (400 MHz, $CDCl_3$) δ 7.35 (dt, $J$ = 8.4, 2.2 Hz, 2H), 7.32 (dt, $J$ = 8.4, 2.2 Hz, 2H), 7.16 (dt, $J$ = 8.4, 2.2 Hz, 2H), 7.06 (dt, $J$ = 8.8, 2.2 Hz, 2H), 4.51–4.45 (m, 1H), 3.49 (dd, $J$ = 10.7, 4.3 Hz, 1H), 3.42 (dd, $J$ = 10.5, 6.4 Hz, 1H), 2.76–2.69 (m, 1H), 2.65–2.58 (m, 1H), 2.11–2.04 (m, 1H), 1.93–1.84 (m, 1H), 1.31 (s, 9H), 1.30 (s, 9H); $^{13}$C NMR (100 MHz, $CDCl_3$) δ 172.9, 150.3, 149.9, 139.2, 138.0, 127.7, 127.6, 125.5, 125.2, 77.8, 56.1, 34.43, 34.38, 34.0, 31.8, 31.3, 24.2; IR (neat): 2962, 2903, 2868, 1734, 1509, 1463, 1363, 1268, 1164, 1138, 1108, 911, 827, 735 cm$^{-1}$; HRMS (FAB) calcd for $C_{26}H_{34}BrO_2$: 457.1742 ([M+H]$^+$), found 457.1742.

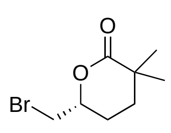

**3e**: $[\alpha]^{26}_{D}$ +5.2 ($c$ = 1.1, $CHCl_3$, 69:31 er); HPLC analysis: Daicel Chiralpak IC-3, hexane/2-propanol = 3:1, flow rate = 0.5 mL/min, 214 nm; retention time: 19.6 min (major) and 23.4 min (minor). $^1$H NMR (400 MHz,

$CDCl_3$) δ 4.54–4.48 (m, 1H), 3.53 (dd, *J* = 10.8, 4.4 Hz, 1H), 3.50 (dd, *J* = 10.6, 5.8 Hz, 1H), 2.05–1.89 (m, 2H), 1.86–1.71 (m, 2H), 1.32 (s, 3H), 1.31 (s, 3H); $^{13}$C NMR (100 MHz, $CDCl_3$) δ 176.3, 79.1, 37.9, 34.3, 33.7, 27.6, 27.4, 24.2; IR (neat): 2966, 2871, 1729, 1387, 1287, 1146, 1121, 1016, 749 cm$^{-1}$; HRMS (EI) calcd for $C_8H_{13}BrO_2$: 220.0099 ($[M]^+$), found 220.0097.

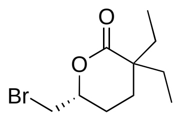


**3f**: $[\alpha]^{23}_{D}$ +4.1 (*c* = 0.91, $CHCl_3$, 79:21 er); HPLC analysis: Daicel Chiralpak IC-3, hexane/2-propanol = 10:1, flow rate = 0.5 mL/min, 214 nm; retention time: 24.4 min (major) and 28.2 min (minor). $^{1}$H NMR (400 MHz, $CDCl_3$) δ 4.49–4.40 (m, 1H), 3.52 (dd, *J* = 10.9, 4.0 Hz, 1H), 3.46 (dd, *J* = 10.6, 6.3 Hz, 1H), 2.10–1.99 (m, 1H), 1.89–1.72 (m, 5H), 1.68–1.58 (m, 1H), 1.53–1.46 (m, 1H), 0.92 (t, *J* = 7.4 Hz, 3H), 0.91 (t, *J* = 7.4 Hz, 3H); $^{13}$C NMR (100 MHz, $CDCl_3$) δ 175.2, 78.7, 45.7, 34.1, 31.8, 31.0, 27.3, 24.8, 8.7, 8.6; IR (neat): 2964, 2925, 1723, 1461, 1249, 1139, 1116, 1034, 948 cm$^{-1}$; HRMS (FAB) calcd for $C_{10}H_{18}BrO_2$: 249.0490 ($[M+H]^+$), found 249.0490.

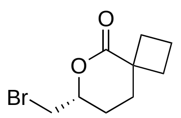


**3g**: $[\alpha]^{25}_{D}$ +5.6 (*c* = 1.1, $CHCl_3$, 72:28 er); HPLC analysis: Daicel Chiralpak IC-3, hexane/2-propanol = 10:1, flow rate = 0.5 mL/min, 254 nm; retention time: 40.4 min (major) and 50.4 min (minor). $^{1}$H NMR (400 MHz, $CDCl_3$) δ 4.47–4.41 (m, 1H), 3.50 (dd, *J* = 10.7, 4.3 Hz, 1H), 3.44 (dd, *J* = 10.7, 6.2 Hz, 1H), 2.73–2.65 (m, 1H), 2.59–2.51 (m, 1H), 2.16–1.70 (m, 8H); $^{13}$C NMR (100 MHz, $CDCl_3$) δ 175.3, 78.0, 42.6, 34.0, 32.3, 31.0, 30.5, 24.4, 15.5; IR (neat): 2928, 2855, 1732, 1306, 1151, 1113, 1090, 1012 cm$^{-1}$; HRMS (EI) calcd for $C_9H_{13}BrO_2$: 232.0099 ($[M]^+$), found 232.0099.

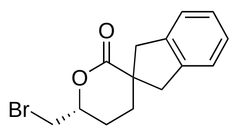


**3h**: $[\alpha]^{25}_{D}$ +28.4 (*c* = 1.3, $CHCl_3$, 80:20 er); HPLC analysis: Daicel Chiralpak IE-3, hexane/2-propanol = 2:1, flow rate = 0.5 mL/min, 254 nm; retention time: 22.2 min (major) and 23.7 min (minor). $^{1}$H NMR (400 MHz, $CDCl_3$) δ 7.22–7.16 (m, 4H), 4.65–4.59 (m, 1H), 3.79 (d, *J* = 15.8 Hz, 1H), 3.60–3.53 (m, 2H), 3.48 (d, *J* = 15.8 Hz, 1H), 3.05 (d, *J* = 15.8 Hz, 1H), 2.89 (d, *J* = 15.8 Hz, 1H), 2.13–1.86 (m, 4H); $^{13}$C NMR (100 MHz, $CDCl_3$) δ 175.6, 140.7, 139.5, 127.0, 126.8, 124.6, 124.5, 78.7, 48.7, 45.6, 45.2, 34.3, 31.6, 24.6; IR (neat): 2937, 1731, 1302, 1228, 1150, 1101, 1071, 989, 913, 747 cm$^{-1}$; HRMS (EI) calcd for $C_{14}H_{15}BrO_2$:

294.0255 ([M]$^+$), found 294.0255.

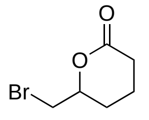

**3i**:[S9] HPLC analysis: Daicel Chiralpak IE-3, hexane/2-propanol = 5:1, flow rate = 0.5 mL/min, 214 nm; retention time: 34.0 min (major) and 37.1 min (minor). $^1$H NMR (400 MHz, $CDCl_3$) δ 4.54–4.48 (m, 1H), 3.54 (ddd, *J* = 10.7, 4.5, 0.8 Hz, 1H), 3.48 (dd, *J* = 10.7, 6.2 Hz, 1H), 2.67–2.59 (m, 1H), 2.53–2.44 (m, 1H), 2.18–2.10 (m, 1H), 2.04–1.83 (m, 2H), 1.77–1.67 (m, 1H); $^{13}$C NMR (100 MHz, $CDCl_3$) δ 170.4, 78.6, 33.7, 29.4, 26.3, 18.1; IR (neat): 1736, 1234, 1041 cm$^{-1}$; HRMS (EI) calcd for $C_6H_9BrO_2$: 191.9786 ([M]$^+$), found 191.9788.

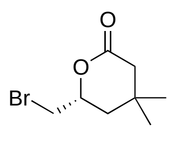

**3j**:[S10] $[\alpha]^{21}_{D}$ +3.0 (*c* = 1.2, $CHCl_3$, 84:16 er); HPLC analysis: Daicel Chiralpak IE-3, hexane/2-propanol = 5:1, flow rate = 0.5 mL/min, 214 nm; retention time: 25.0 min (major) and 27.2 min (minor). $^1$H NMR (400 MHz, $CDCl_3$) δ 4.62–4.55 (m, 1H), 3.54 (dd, *J* = 10.8, 4.8 Hz, 1H), 3.51 (dd, *J* = 10.8, 5.6 Hz, 1H), 2.40 (dd, *J* = 16.8, 2.2 Hz, 1H), 2.26 (d, *J* = 16.9 Hz, 1H), 1.87 (ddd, *J* = 14.0, 4.0, 2.0 Hz, 1H), 1.62 (dd, *J* = 14.0, 11.9 Hz, 1H), 1.12 (s, 3H), 1.11 (s, 3H); $^{13}$C NMR (100 MHz, $CDCl_3$) δ 170.5, 75.8, 43.6, 40.1, 34.4, 30.7, 29.8, 26.9; IR (neat): 2960, 1739, 1371, 1238, 1198, 1054, 1044 cm$^{-1}$; HRMS (EI) calcd for $C_8H_{13}BrO_2$: 220.0099 ([M]$^+$), found 220.0099.

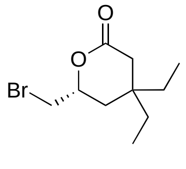

**3k**: $[\alpha]^{19}_{D}$ –11.2 (*c* = 1.1, $CHCl_3$, 89:11 er); HPLC analysis: Daicel Chiralpak IG-3, hexane/2-propanol = 5:1, flow rate = 0.5 mL/min, 214 nm; retention time: 22.1 min (major) and 24.1 min (minor). $^1$H NMR (400 MHz, $CDCl_3$) δ 4.51–4.45 (m, 1H), 3.53 (dd, *J* = 10.6, 4.7 Hz, 1H), 3.47 (dd, *J* = 10.6, 6.1 Hz, 1H), 2.37 (dd, *J* = 16.5, 1.4 Hz, 1H), 2.26 (d, *J* = 16.2 Hz, 1H), 1.94 (ddd, *J* = 14.2, 3.3, 1.3 Hz, 1H), 1.53–1.35 (m, 5H), 0.87 (t, *J* = 7.2 Hz, 3H), 0.85 (t, *J* = 7.6 Hz, 3H); $^{13}$C NMR (100 MHz, $CDCl_3$) δ 171.4, 75.4, 40.2, 36.4, 35.3, 33.9, 31.7, 29.2, 7.6, 7.3; IR (neat): 2965, 2927, 1748, 1457, 1248, 1218, 1053 cm$^{-1}$; HRMS (EI) calcd for $C_{10}H_{17}BrO_2$: 248.0412 ([M]$^+$), found 248.0415.

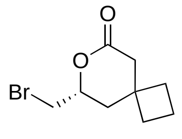

**3l**: $[\alpha]^{20}_{D}$ +9.7 (*c* = 1.4, $CHCl_3$, 81:19 er); HPLC analysis: Daicel Chiralpak IE-3, hexane/2-propanol = 5:1, flow rate = 0.5 mL/min, 214 nm; retention time: 30.2 min (major) and 31.8 min (minor). $^1$H NMR (400

MHz, $CDCl_3$) δ 4.53–4.46 (m, 1H), 3.54 (dd, $J$ = 10.8, 4.4 Hz, 1H), 3.51 (dd, $J$ = 10.8, 6.0 Hz, 1H), 2.80 (dd, $J$ = 17.5, 2.2 Hz, 1H), 2.43 (d, $J$ = 17.6 Hz, 1H), 2.18 (ddd, $J$ = 13.7, 3.5, 2.2 Hz, 1H), 2.02–1.87 (m, 6H), 1.75 (dd, $J$ = 13.7, 11.4 Hz, 1H); $^{13}$C NMR (100 MHz, $CDCl_3$) δ 170.1, 75.5, 42.3, 38.4, 36.4, 34.2, 33.1, 31.9, 14.9; IR (neat): 2950, 1739, 1236, 1195, 1123, 1036, 913, 741 cm$^{-1}$; HRMS (EI) calcd for $C_9H_{14}BrO_2$: 233.0177 ([M+H]$^+$), found 233.0175.

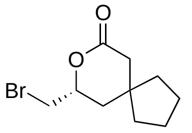


**3m**: $[\alpha]^{21}_D$ +5.1 ($c$ = 1.2, $CHCl_3$, 82:18 er); HPLC analysis: Daicel Chiralpak IG-3, hexane/2-propanol = 5:1, flow rate = 0.5 mL/min, 214 nm; retention time: 25.5 min (major) and 27.7 min (minor). $^1$H NMR (400 MHz, $CDCl_3$) δ 4.59–4.53 (m, 1H), 3.54 (dd, $J$ = 10.8, 4.8 Hz, 1H), 3.51 (dd, $J$ = 10.8, 5.6 Hz, 1H), 2.52 (dd, $J$ = 17.2, 2.1 Hz, 1H), 2.31 (d, $J$ = 17.2 Hz, 1H), 1.91 (ddd, $J$ = 13.6, 3.6, 2.2 Hz, 1H), 1.79–1.63 (m, 5H), 1.61–1.48 (m, 4H); $^{13}$C NMR (100 MHz, $CDCl_3$) δ 170.4, 76.5, 41.9, 40.54, 40.52, 38.6, 36.9, 34.5, 24.1, 23.5; IR (neat): 2952, 2864, 1733, 1245, 1099, 1056, 1041 cm$^{-1}$; HRMS (EI) calcd for $C_{10}H_{15}BrO_2$: 246.0255 ([M]$^+$), found 246.0255.

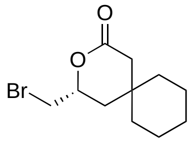


**3n**: $[\alpha]^{19}_D$ –9.8 ($c$ = 0.95, $CHCl_3$, 85:15 er); HPLC analysis: Daicel Chiralpak IG-3, hexane/2-propanol = 5:1, flow rate = 0.5 mL/min, 214 nm; retention time: 26.9 min (major) and 29.8 min (minor). $^1$H NMR (400 MHz, $CDCl_3$) δ 4.56–4.50 (m, 1H), 3.53 (dd, $J$ = 10.8, 4.8 Hz, 1H), 3.49 (dd, $J$ = 10.8, 6.0 Hz, 1H), 2.49 (dd, $J$ = 16.7, 1.4 Hz, 1H), 2.26 (d, $J$ = 16.5 Hz, 1H), 2.08–2.03 (m, 1H), 1.53–1.33 (m, 11H); $^{13}$C NMR (100 MHz, $CDCl_3$) δ 170.9, 75.2, 41.9, 39.4, 37.8, 35.7, 34.2, 32.7, 25.6, 21.6, 21.3; IR (neat): 2927, 2853, 1741, 1243, 1202, 1102, 1052, 1035, 913, 739 cm$^{-1}$; HRMS (EI) calcd for $C_{11}H_{17}BrO_2$: 260.0412 ([M]$^+$), found 260.0412.

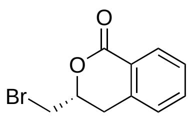


**3o**:[S11] $[\alpha]^{19}_D$ +34.7 ($c$ = 0.83, $CHCl_3$, 67:33 er), $[\alpha]^{21}_D$ +29.1 ($c$ = 1.4, $CH_2Cl_2$, 67:33 er); HPLC analysis: Daicel Chiralpak AS-3, hexane/2-propanol = 1:1, flow rate = 0.5 mL/min, 254 nm; retention time: 18.7 min (major) and 26.2 min (minor). $^1$H NMR (400 MHz, $CDCl_3$) δ 8.11 (dd, $J$ = 7.8, 0.9 Hz, 1H), 7.58 (td, $J$ = 7.4, 1.4 Hz, 1H), 7.43 (t, $J$ = 7.7 Hz, 1H), 7.30 (d, $J$ = 7.5 Hz, 1H), 4.78–4.72 (m, 1H), 3.69 (dd, $J$ = 10.7, 4.6 Hz, 1H), 3.60 (dd, $J$ = 10.7, 7.1 Hz, 1H), 3.24–3.15 (m, 2H); $^{13}$C NMR (100 MHz, $CDCl_3$) δ 164.3, 137.8, 134.1, 130.4, 128.0, 127.7,

124.5, 76.7, 32.4, 31.5; IR (neat): 1727, 1278, 1242, 1120, 1085, 1032, 913, 741 $cm^{-1}$; HRMS (EI) calcd for $C_{10}H_9BrO_2$: 239.9786 ($[M]^+$), found 239.9786.

**Asymmetric bromolactonization of 2a on a larger scale (Scheme 5).**

In a manner similar to our previous report,[S8] a solution of substrate **2a** (266 mg, 1.0 mmol) and catalyst (*S*)-**1g** (60.1 mg, 0.10 mmol, 10 mol %) in $CH_2Cl_2$ (15 mL)-toluene (5 mL) was cooled to –78 °C. After stirring for 10 min at –78 °C, *N*-bromophthalimide (NBP) (271 mg, 1.2 mmol) was added to the cooled reaction solution. The reaction mixture was stirred for 24 h at –78 °C. After 24 h, the reaction mixture was quenched with saturated aqueous $Na_2SO_3$ (30 mL) at –78 °C and stirred for 10 min at –78 °C. The quenched reaction mixture was diluted with $CH_2Cl_2$ (20 mL) and $H_2O$ (20 mL), and the mixture was warmed to room temperature. The organic materials were extracted with $CH_2Cl_2$ for three times (20 mL × 3). The combined extracts were dried over $Na_2SO_4$ and concentrated. The residue was purified by flash column chromatography on silica gel (hexane/ethyl acetate = 40:1–3:1) to give product **3a** in 91% yield (314 mg, 0.91 mmol). The enantioselectivity of isolated product **3a** was determined by HPLC analysis on a chiral stationary phase (Daicel Chiralpak IE-3, hexane/2-propanol = 2:1, flow rate = 0.5 mL/min, 254 nm).

**Transformations of 3a (Scheme 5).**

In a manner similar to our previous report,[S8] to a solution of **3a** (34.5 mg, 0.10 mmol) in $CH_3CN$ (1 mL) was added $K_2CO_3$ (41.5 mg, 0.30 mmol) and thiophenol (33.1 mg, 0.30 mmol). The reaction mixture was warmed to 75 °C and stirred for 3 h at 75 °C. After 3 h, the reaction mixture was cooled to 0 °C and quenched with saturated aqueous $NH_4Cl$ (5 mL). After evaporation to remove $CH_3CN$, the organic materials were extracted with ethyl acetate for three times (5 mL × 3). The combined extracts were dried over $Na_2SO_4$ and concentrated. The residue was purified by flash column chromatography on silica gel (hexane/ethyl acetate = 40:1–3:1 as eluent) to give product **7** in 95% yield (35.6 mg, 0.095 mmol).

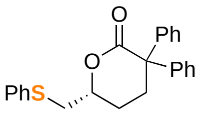

**7**: $[\alpha]^{20}_{D}$ –19.6 ($c$ = 1.1, $CHCl_3$, 89:11 er); HPLC analysis: Daicel Chiralpak IE-3, hexane/2-propanol = 2:1, flow rate = 0.5 mL/min, 254 nm; retention time: 37.9 min (major) and 39.5 min (minor). $^{1}H$ NMR (400 MHz, $CDCl_3$) δ 7.35–7.24 (m, 10H), 7.22–7.15 (m, 5H), 4.46–4.39 (m, 1H), 3.32 (dd, $J$ = 13.7, 4.6 Hz, 1H), 2.95 (dd, $J$ = 13.7, 8.2 Hz, 1H), 2.74–2.59 (m, 2H), 2.18–2.05 (m, 1H), 1.83–1.70 (m, 1H); $^{13}C$ NMR (100 MHz, $CDCl_3$) δ 172.8, 142.3, 141.5, 134.9, 129.9, 129.1, 128.4, 128.3, 128.11, 128.06, 127.4, 127.2, 126.7, 78.5, 56.9, 39.0, 31.9, 24.6; IR (neat): 3059, 2960, 2926, 1733, 1496, 1445, 1255, 1166, 1120, 1091, 1054, 745 $cm^{-1}$; HRMS (EI) calcd for $C_{24}H_{22}O_2S$: 374.1341 ($[M]^+$), found 374.1341.

In a manner similar to our previous report,[S8] to a solution of **3a** (34.5 mg, 0.10 mmol) in *N*,*N*-dimethylformamide (2 mL) was added sodium azide (19.5 mg, 0.30 mmol). The reaction mixture was warmed to 100 °C and stirred for 24 h at 100 °C. After 24 h, the reaction mixture was cooled to 0 °C and quenched with $H_2O$ (20 mL). The organic materials were extracted with ethyl acetate for three times (5 mL × 3). The combined extracts were dried over $Na_2SO_4$ and concentrated. The residue was purified by flash column chromatography on silica gel (hexane/ethyl acetate = 40:1–3:1 as eluent) to give product **8** in 96% yield (29.5 mg, 0.096 mmol).

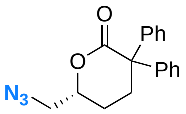

**8**: $[\alpha]^{23}_{D}$ –74.3 ($c$ = 1.2, $CHCl_3$, 89:11 er); HPLC analysis: Daicel Chiralpak IE-3, hexane/2-propanol = 2:1, flow rate = 0.5 mL/min, 254 nm; retention time: 22.1 min (major) and 25.4 min (minor). $^{1}H$ NMR (400 MHz, $CDCl_3$) δ 7.39–7.24 (m, 8H), 7.17–7.14 (m, 2H), 4.45–4.39 (m, 1H), 3.48 (dd, $J$ = 13.2, 4.2 Hz, 1H), 3.35 (dd, $J$ = 13.2, 4.9 Hz, 1H), 2.81–2.74 (m, 1H), 2.70–2.63 (m, 1H), 1.95–1.84 (m, 2H); $^{13}C$ NMR (100 MHz, $CDCl_3$) δ 172.5, 142.2, 140.9, 128.6, 128.4, 128.1, 127.6, 127.3, 77.8, 56.8, 54.4, 31.6, 22.6; IR (neat): 3059, 2955, 2937, 2099, 1732, 1496, 1446, 1287, 1257, 1172, 1122, 1100, 752, 698 $cm^{-1}$; HRMS (EI) calcd for $C_{18}H_{17}N_3O_2$: 307.1321 ($[M]^+$), found 307.1321.

In a manner similar to our previous report,[S8] to a solution of **3a** (34.5 mg, 0.10 mmol) in MeOH (1 mL) was added $K_2CO_3$ (27.6 mg, 0.20 mmol) at room temperature.

The reaction mixture was stirred for 48 h at room temperature. After 48 h, the reaction mixture was quenched with $H_2O$ (10 mL). The organic materials were extracted with ethyl acetate for three times (5 mL × 3). The combined extracts were dried over $Na_2SO_4$ and concentrated. The residue was purified by flash column chromatography on silica gel (hexane/ethyl acetate = 20:1–3:1 as eluent) to give product **9** in 73% yield (21.6 mg, 0.073 mmol).

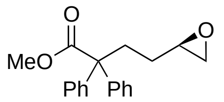


**9**: $[\alpha]^{22}_{D}$ +2.6 (*c* = 0.99, $CHCl_3$, 89:11 er); HPLC analysis: Daicel Chiralpak IB-3, hexane/2-propanol = 20:1, flow rate = 0.5 mL/min, 254 nm; retention time: 12.7 min (major) and 15.0 min (minor). $^1H$ NMR (400 MHz, $CDCl_3$) δ 7.33–7.22 (m, 10H), 3.69 (s, 3H), 2.88–2.83 (m, 1H), 2.70 (dd, *J* = 4.8, 4.1 Hz, 1H), 2.59 (ddd, *J* = 13.2, 11.2, 5.2 Hz, 1H), 2.48 (ddd, *J* = 13.2, 11.2, 5.2 Hz, 1H), 2.38 (dd, *J* = 4.8, 2.8 Hz, 1H), 1.39–1.23 (m, 2H); $^{13}C$ NMR (100 MHz, $CDCl_3$) δ 174.6, 142.5, 142.4, 128.82, 128.80, 128.0, 126.93, 126.89, 59.9, 52.4, 52.3, 47.0, 34.2, 28.5; IR (neat): 3057, 2950, 1729, 1496, 1445, 1224, 731, 700 $cm^{-1}$; HRMS (EI) calcd for $C_{19}H_{20}O_3$: 296.1412 ($[M]^+$), found 296.1412.

**Determination of the absolute configuration of products.**

Absolute stereochemistry of bromolactonization product **3o** was confirmed by comparison with reported data.[S11]

## References


S1. Nishiyori, R.; Tsuchihashi, A.; Mochizuki, A.; Kaneko, K.; Yamanaka, M.; Shirakawa, S. *Chem. – Eur. J.* **2018**, *24*, 16747–16752.

S2. Mori, T.; Abe, K.; Shirakawa, S. *J. Org. Chem.* **2023**, *88*, 7830–7838.

S3. Okada, M.; Kaneko, K.; Yamanaka, M.; Shirakawa, S. *Org. Biomol. Chem.* **2019**, *17*, 3747–3751.

S4. Nishiyori, R.; Okada, M.; Maynard, J. R. J.; Shirakawa, S. *Asian J. Org. Chem.* **2021**, *10*, 1444–1448.

S5. Tsuchihashi, A.; Shirakawa, S. *Synlett* **2019**, *30*, 1662–1666.

S6. Takenaka, K.; Akita, M.; Tanigaki, Y.; Takizawa, S.; Sasai, H. *Org. Lett.* **2011**, *13*, 3506–3509.

S7. Tsutsumi, T.; Saitoh, A.; Kasai, T.; Chu, MY.; Karanjit, S.; Nakayama, A.; Namba, K. *Tetrahedron Lett.* **2020**, *61*, 152047.

S8. Mori, T.; Sumida, S.; Furuya, Y.; Shirakawa, S. *Synlett* **2024**, *35*, 479–483.

S9. Moriyama, K.; Kuramochi, M.; Tsuzuki, S.; Fujii, K.; Morita, T. *Org. Lett.* **2021**, *23*, 268–273.

S10. China, H.; Yatabe, H. Kageyama, N.; Fujitake, M.; Kikushima, K.; Dohi, T. *Russ. Chem. Bull.* **2020**, *69*, 1804–1810.

S11. Armstrong, A.; Braddock, D. C.; Jones, A. X.; Clark, S. *Tetrahedron Lett.* **2013**, *54*, 7004–7008.

## NMR Charts

**2b**: $^{1}$H and $^{13}$C NMR

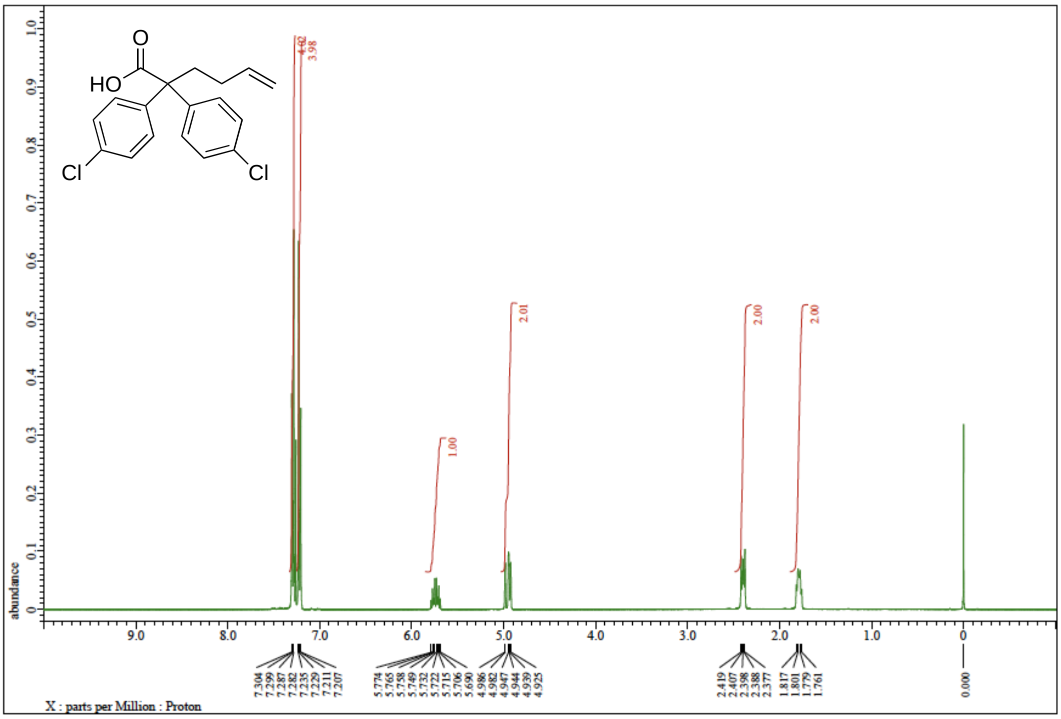


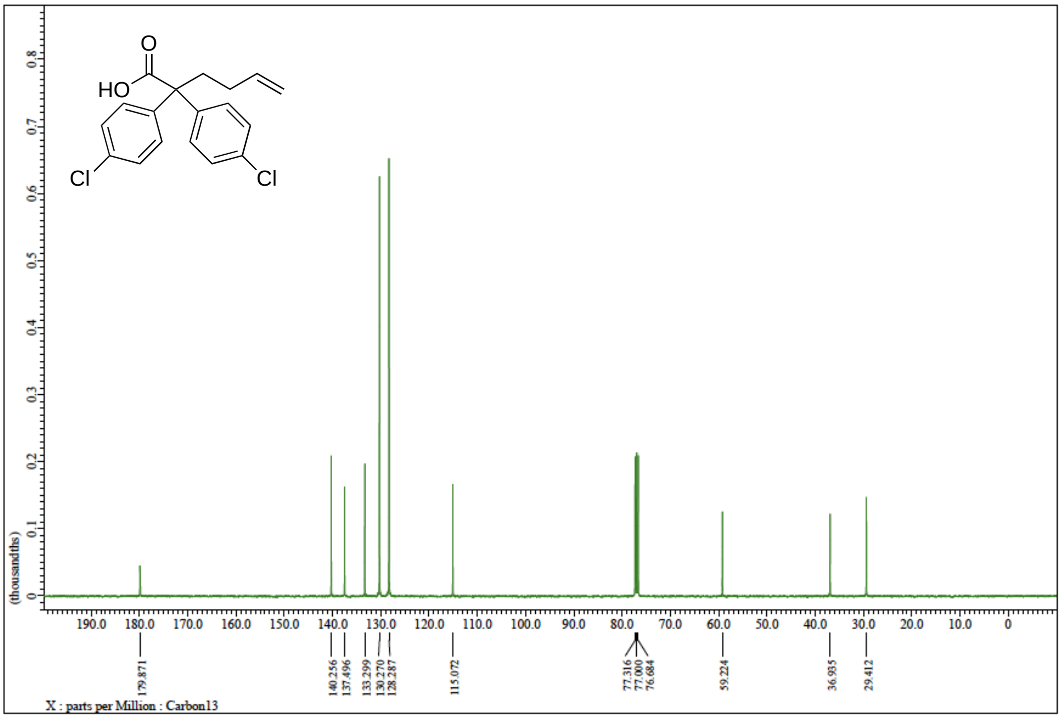

**2c**: $^{1}$H and $^{13}$C NMR

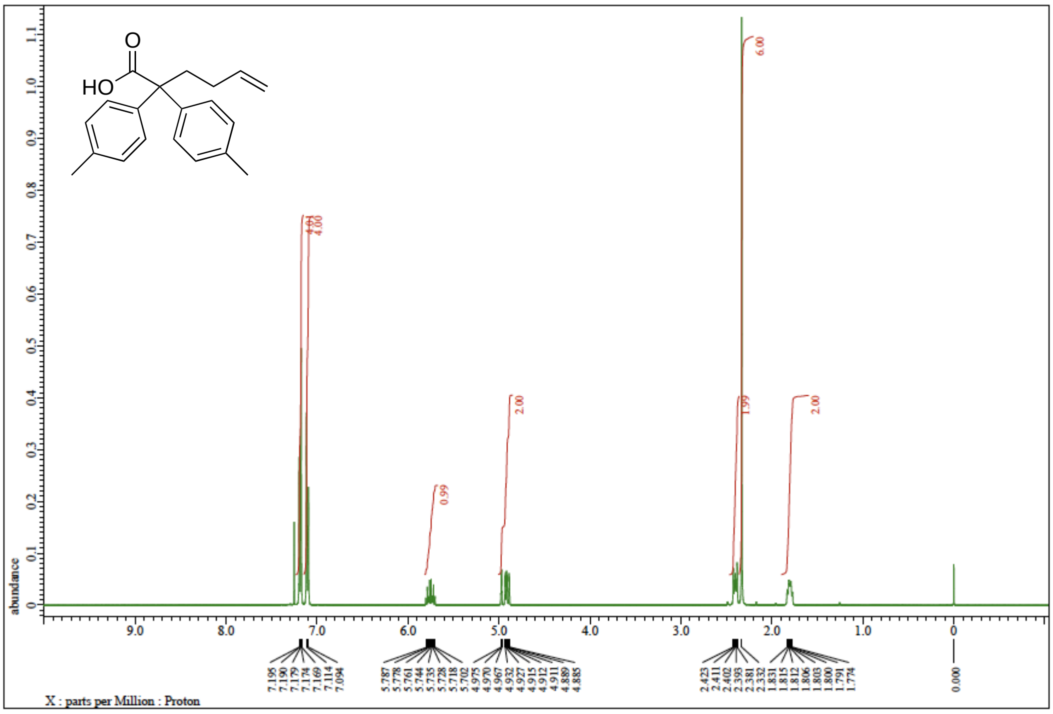

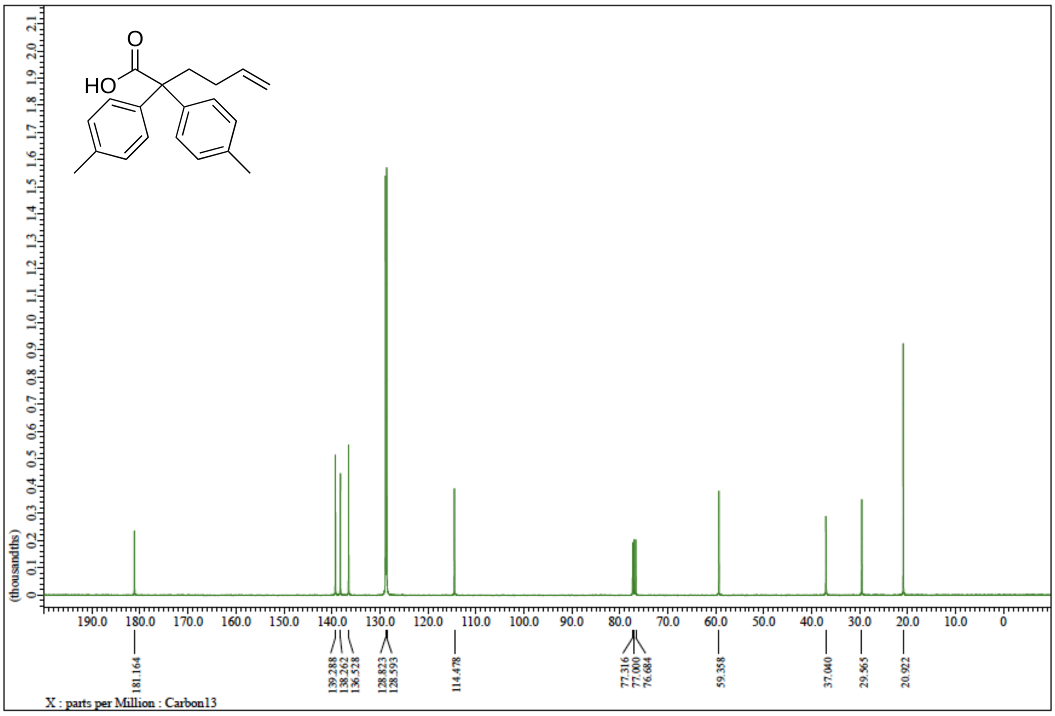

**2d**: $^{1}$H and $^{13}$C NMR

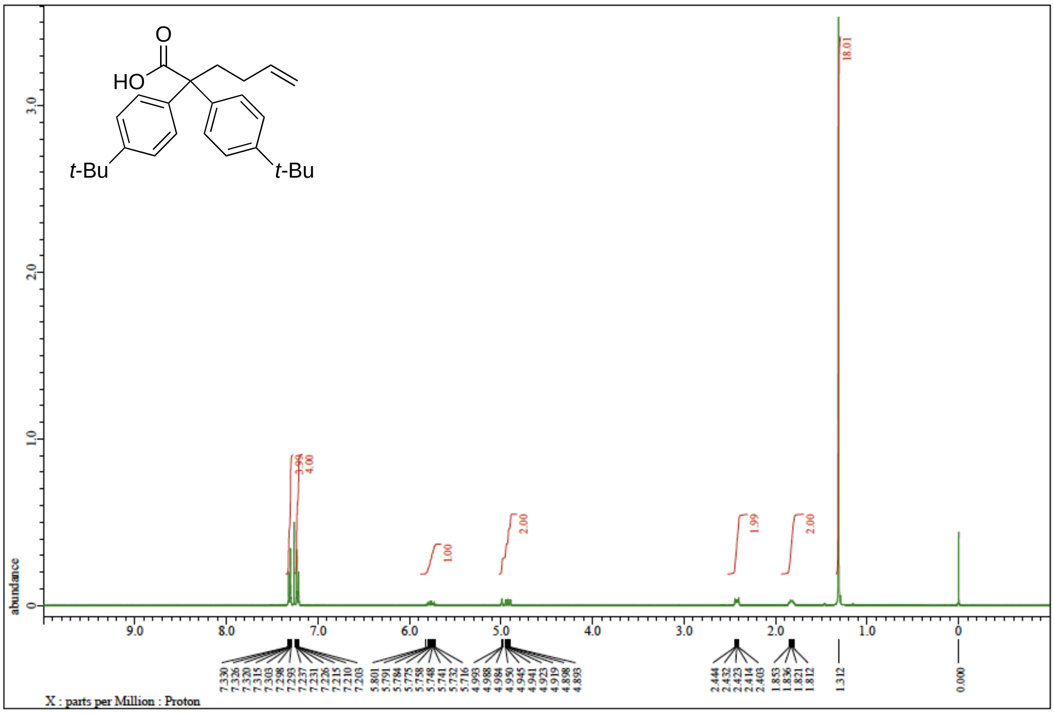

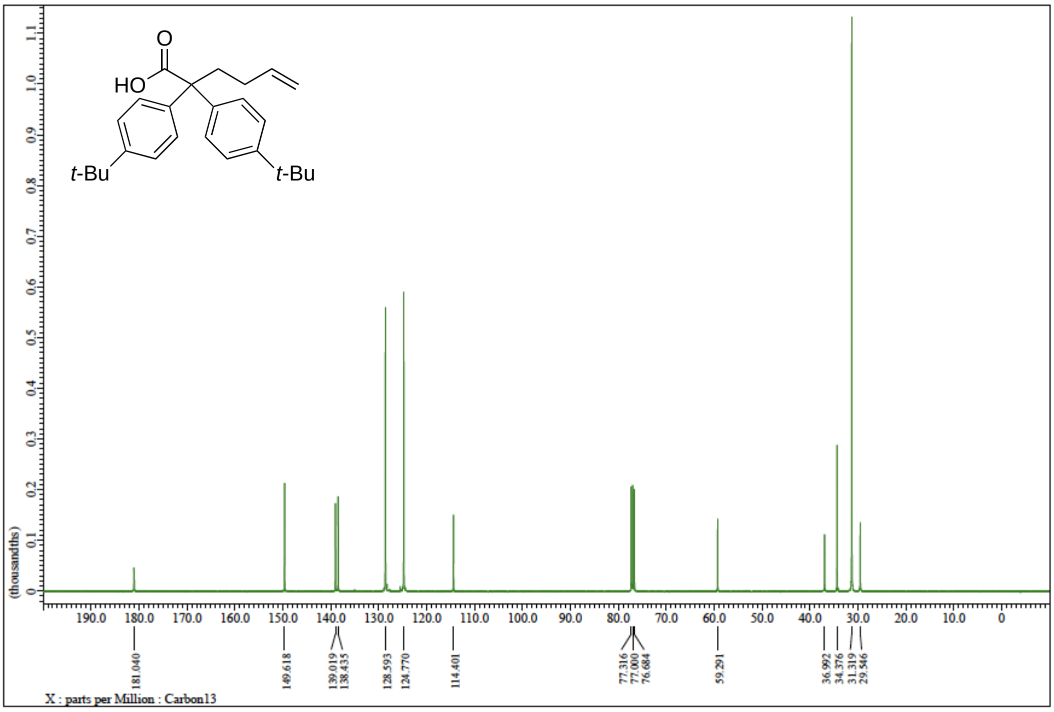

**2k**: $^{1}$H and $^{13}$C NMR

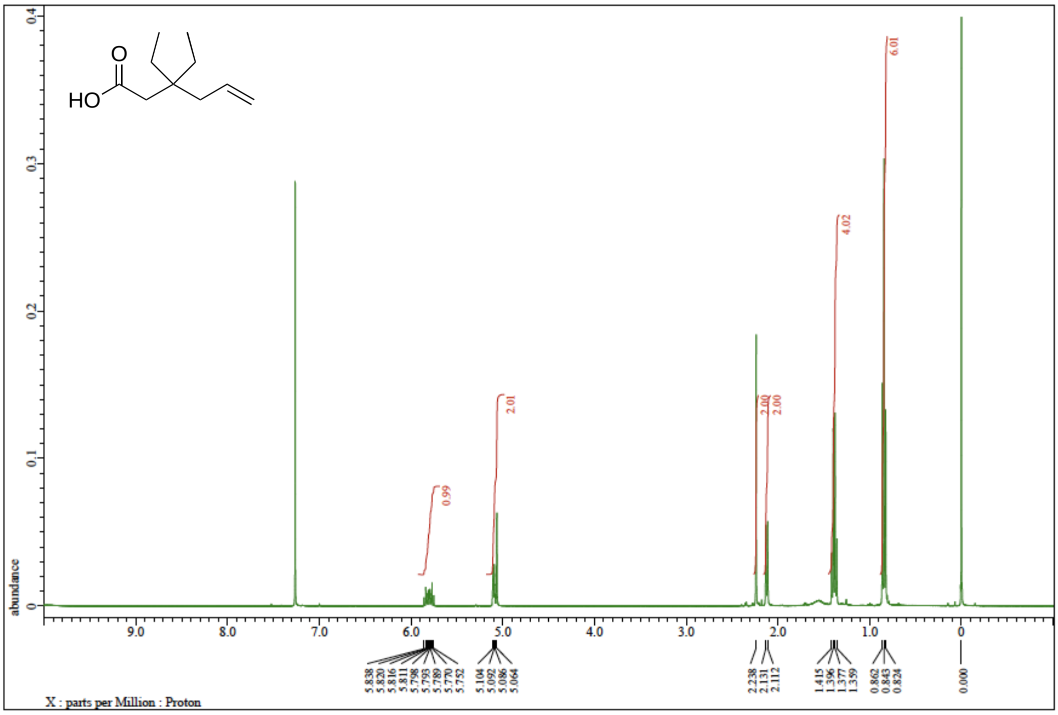


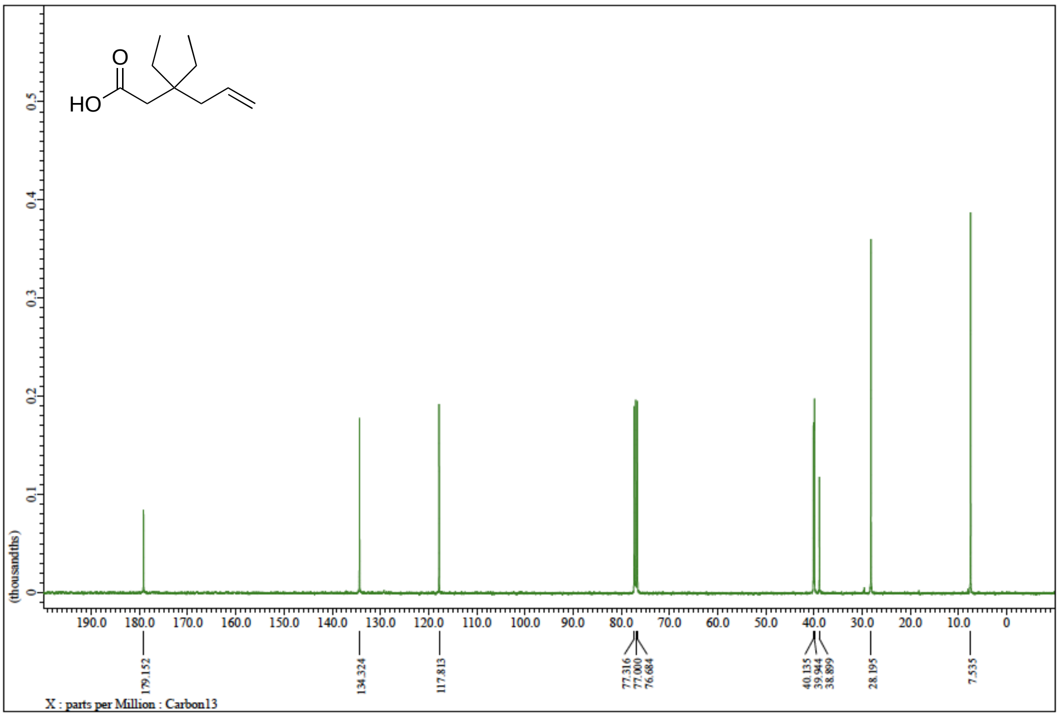

**2l**: $^{1}$H and $^{13}$C NMR

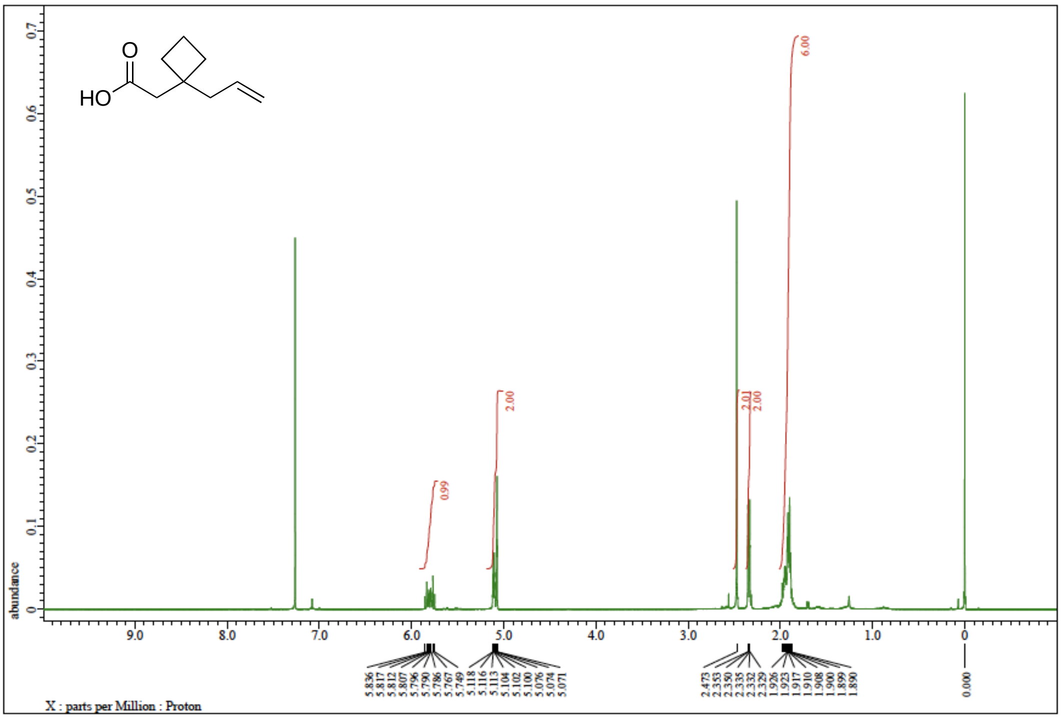

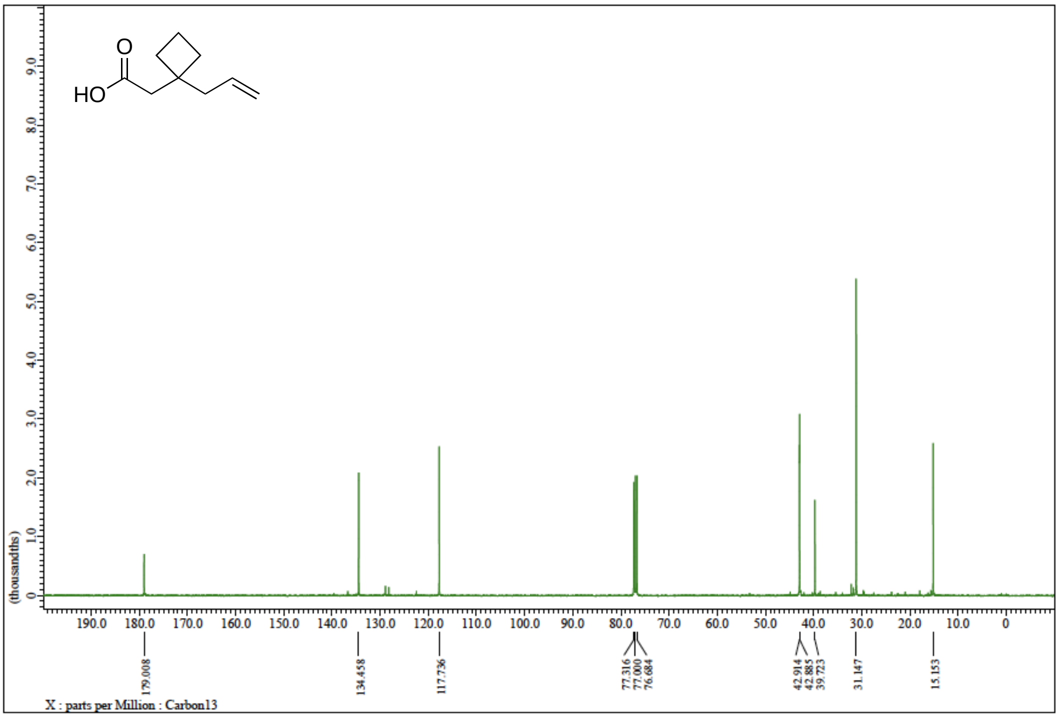

**2m**: $^{1}$H and $^{13}$C NMR

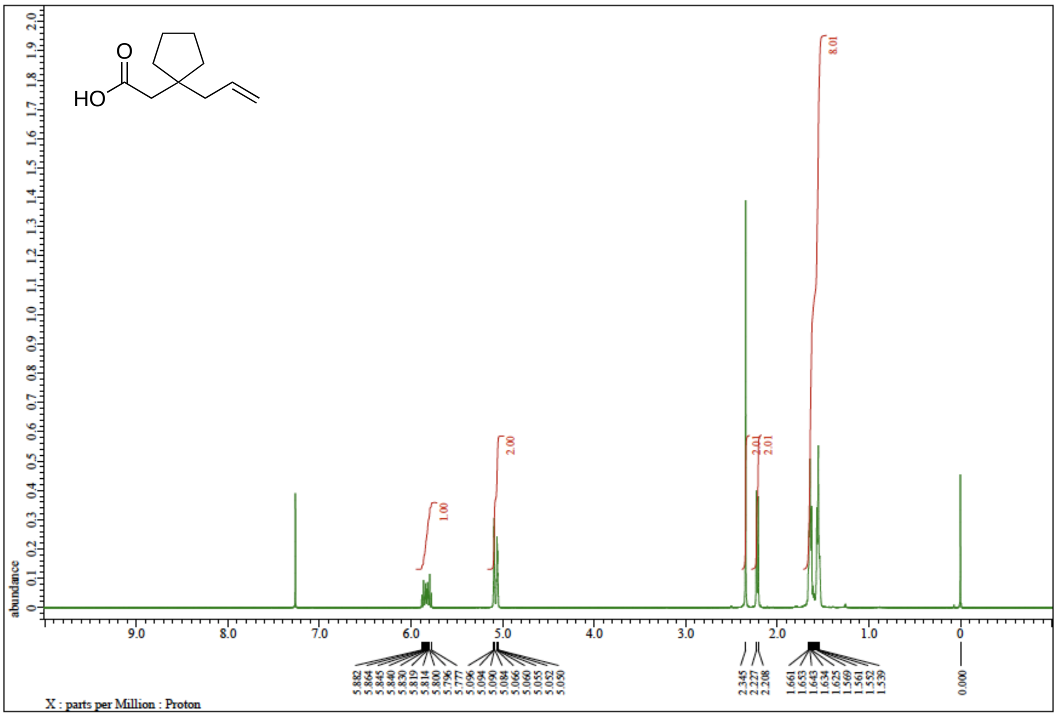


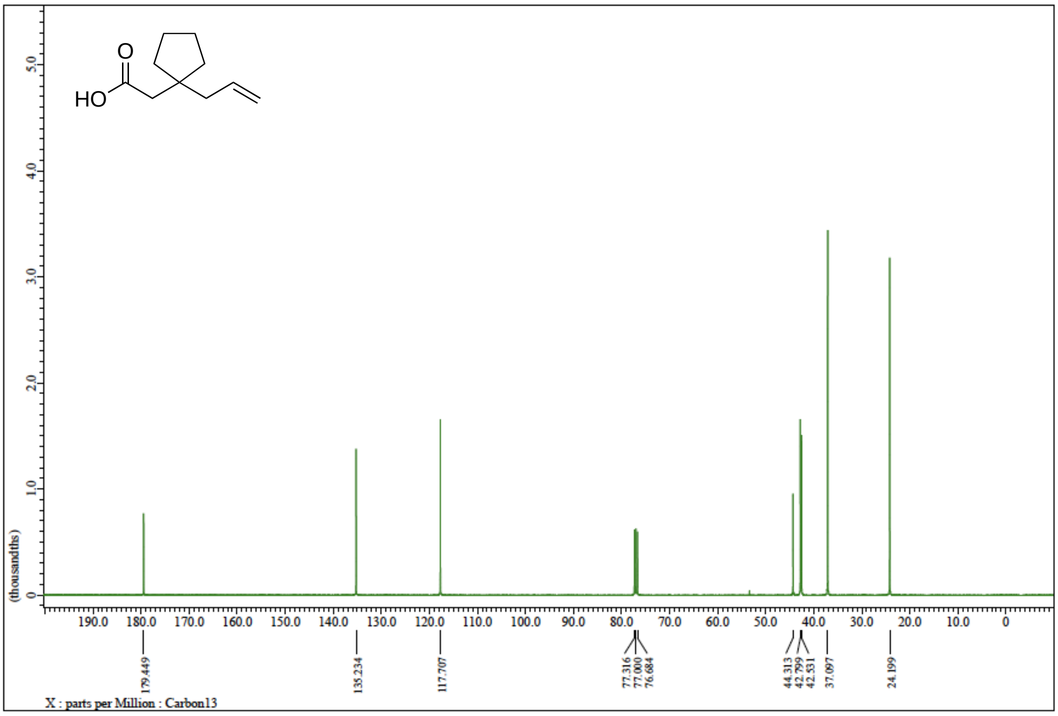

**2n**: $^{1}$H and $^{13}$C NMR

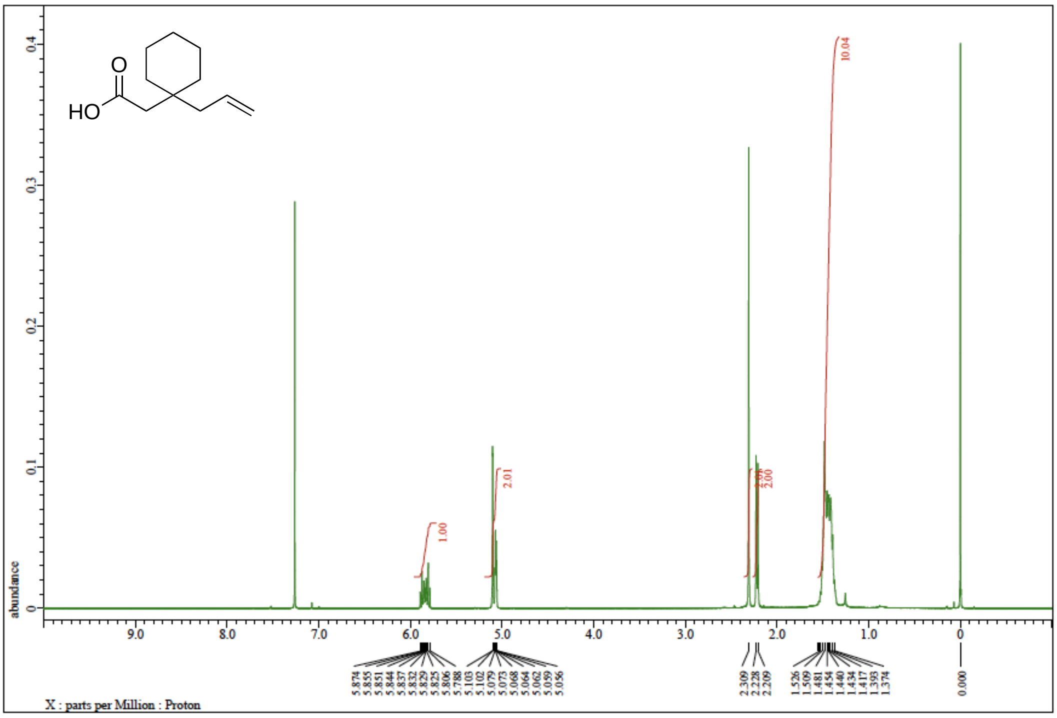


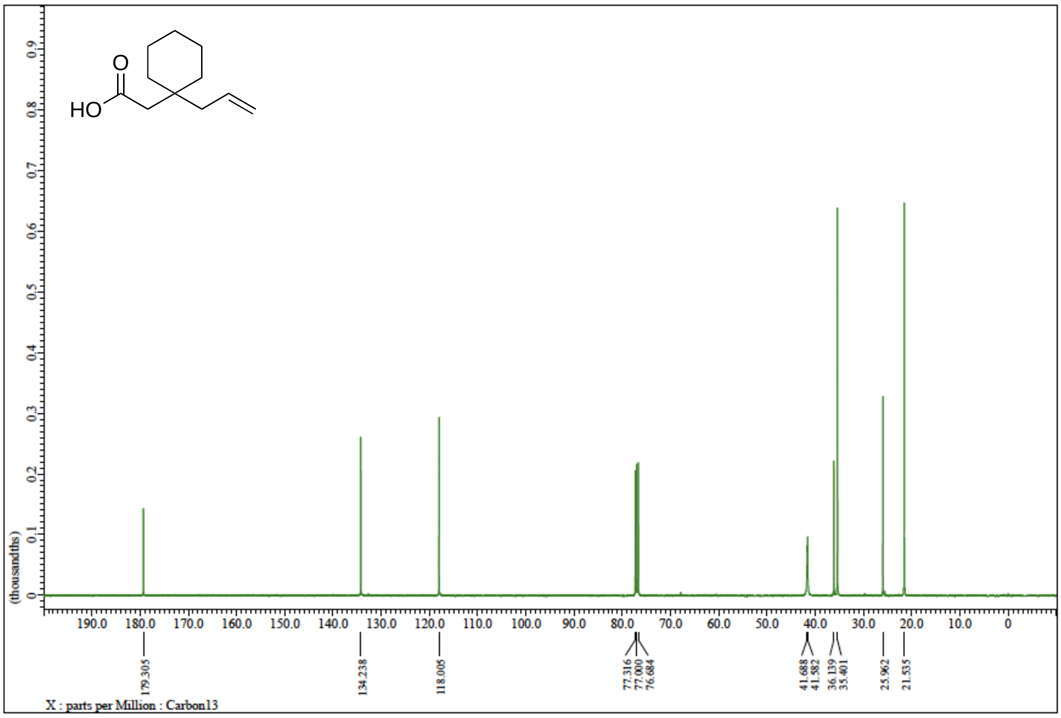

**3a**: $^{1}H$ and $^{13}C$ NMR

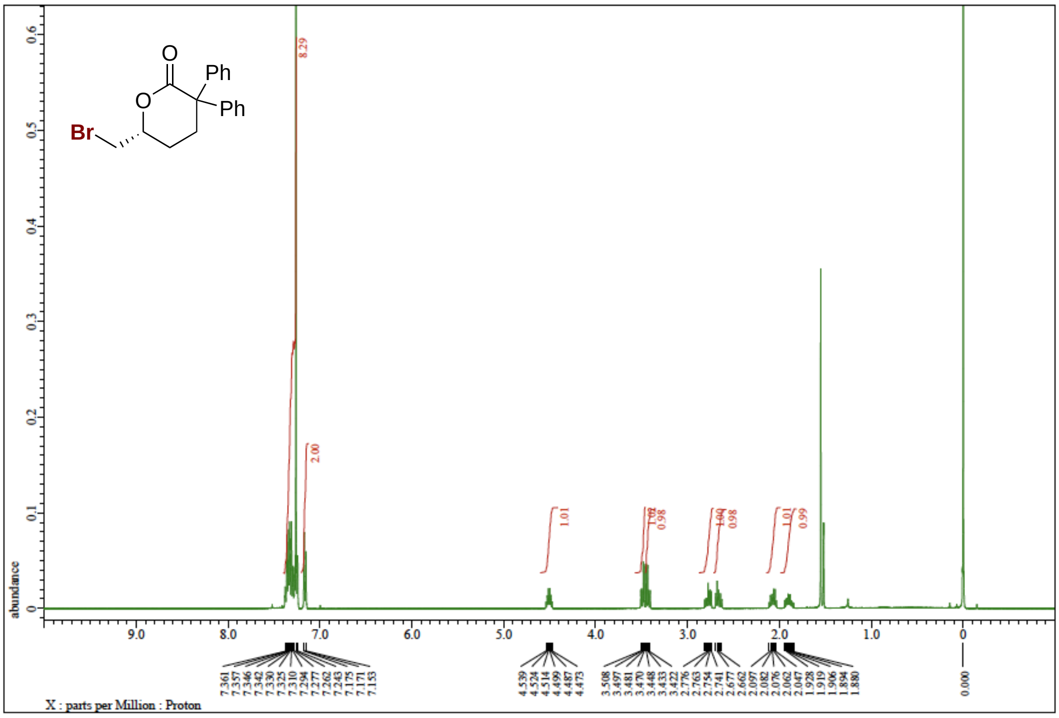

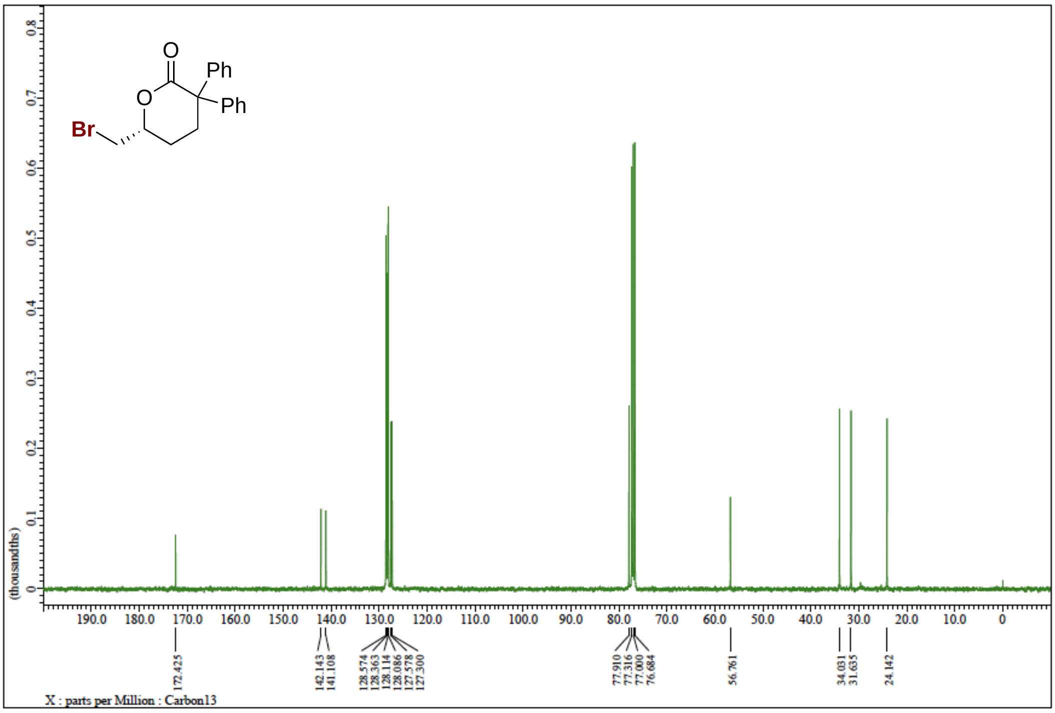

**Iodolactonization Product**: $^{1}$H and $^{13}$C NMR

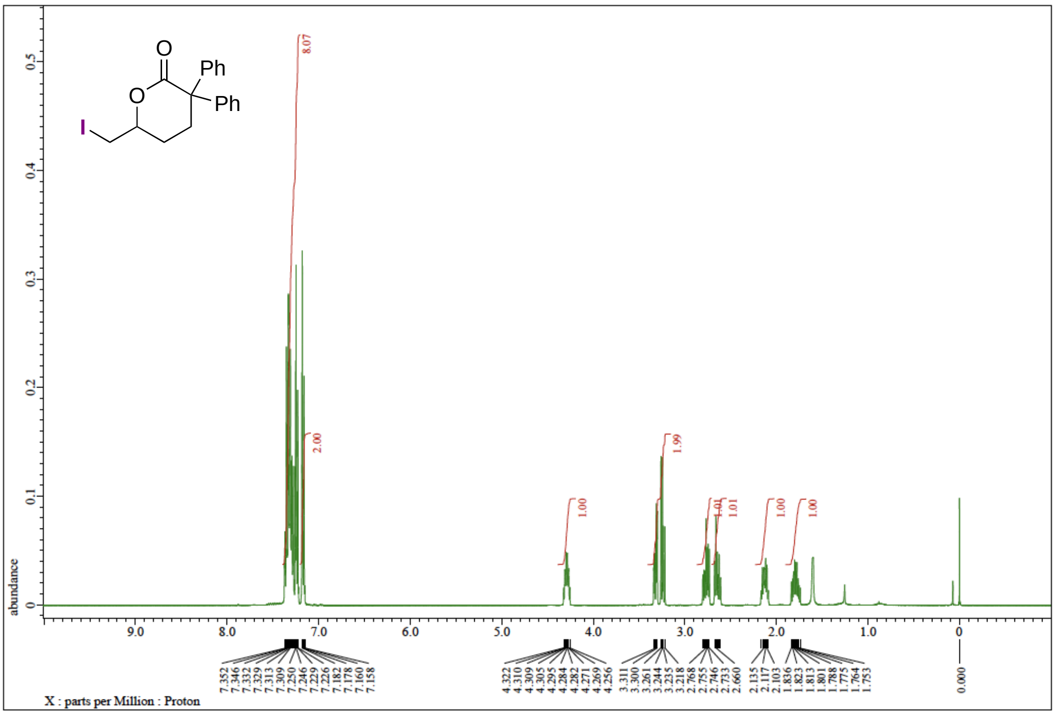


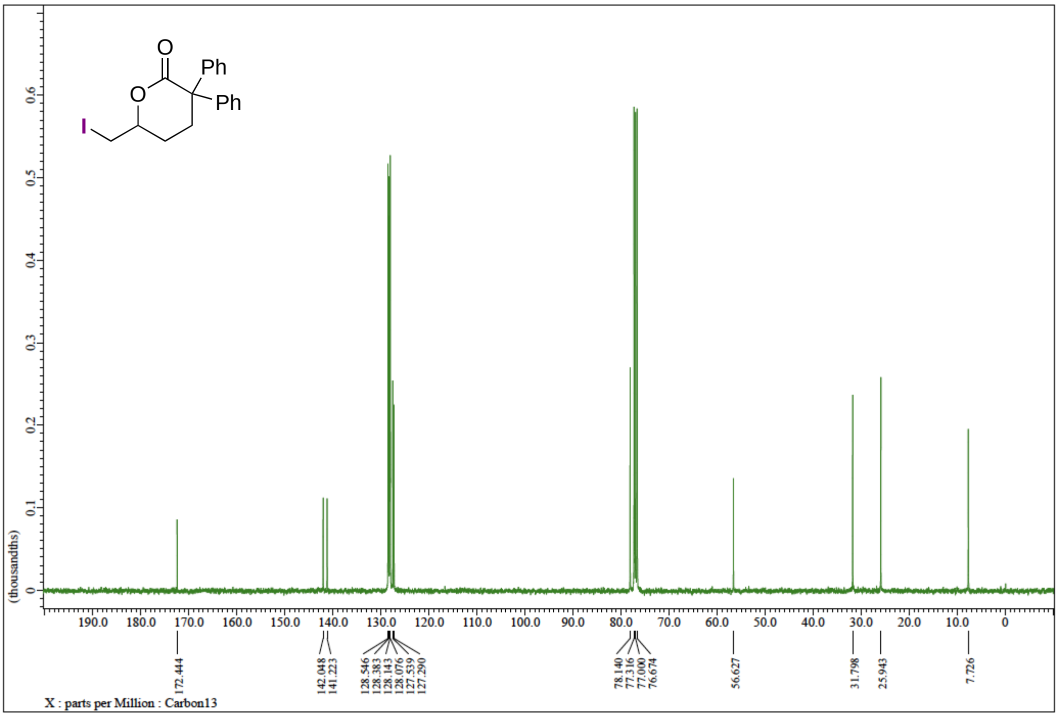

**3b**: $^{1}$H and $^{13}$C NMR

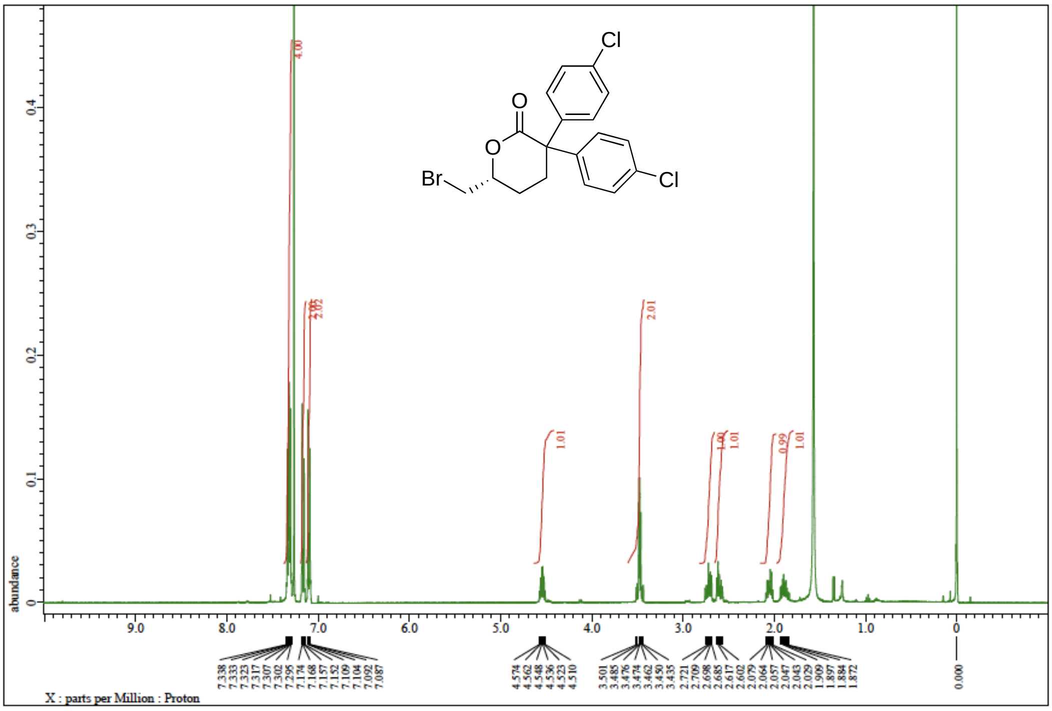


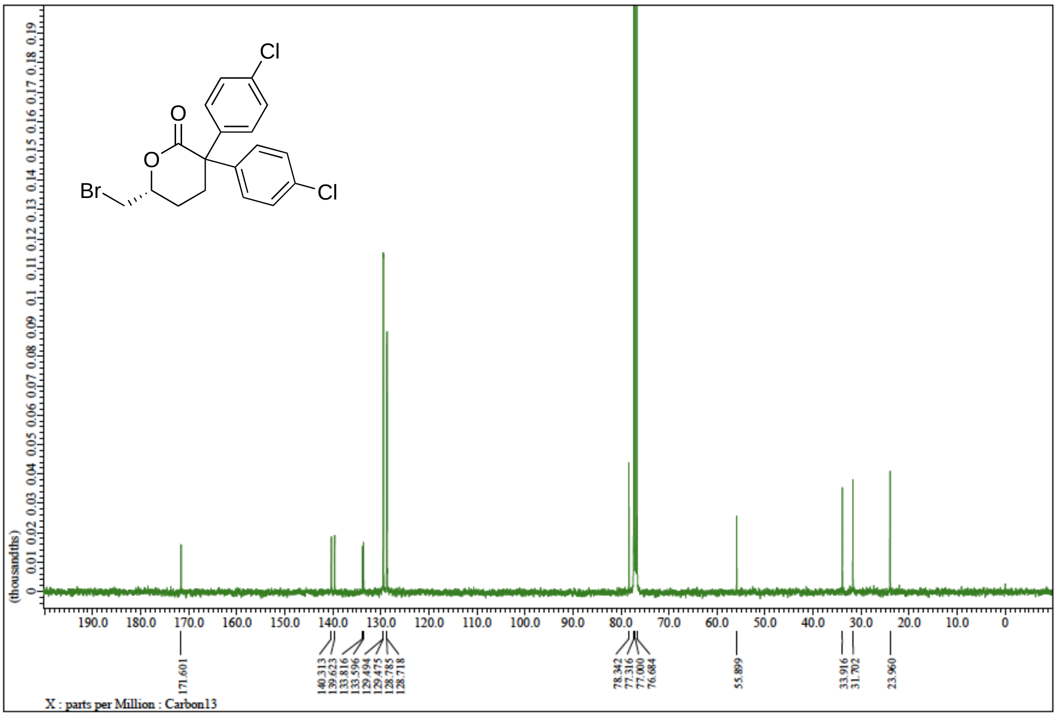

**3c**: $^{1}$H and $^{13}$C NMR

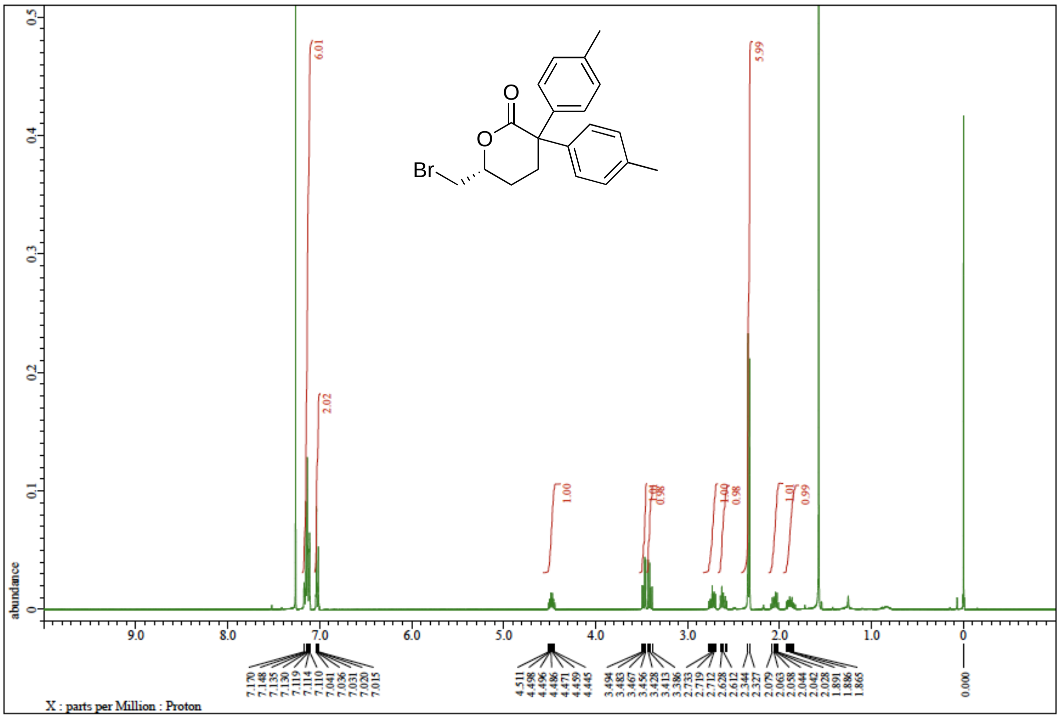

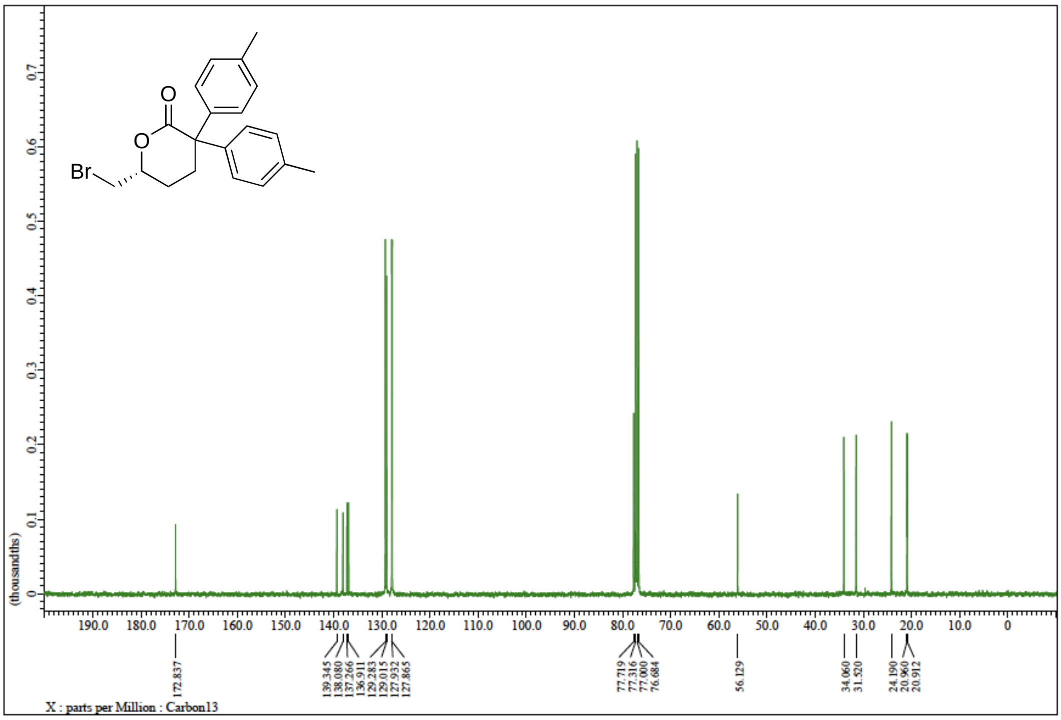

**3d**: $^{1}$H and $^{13}$C NMR

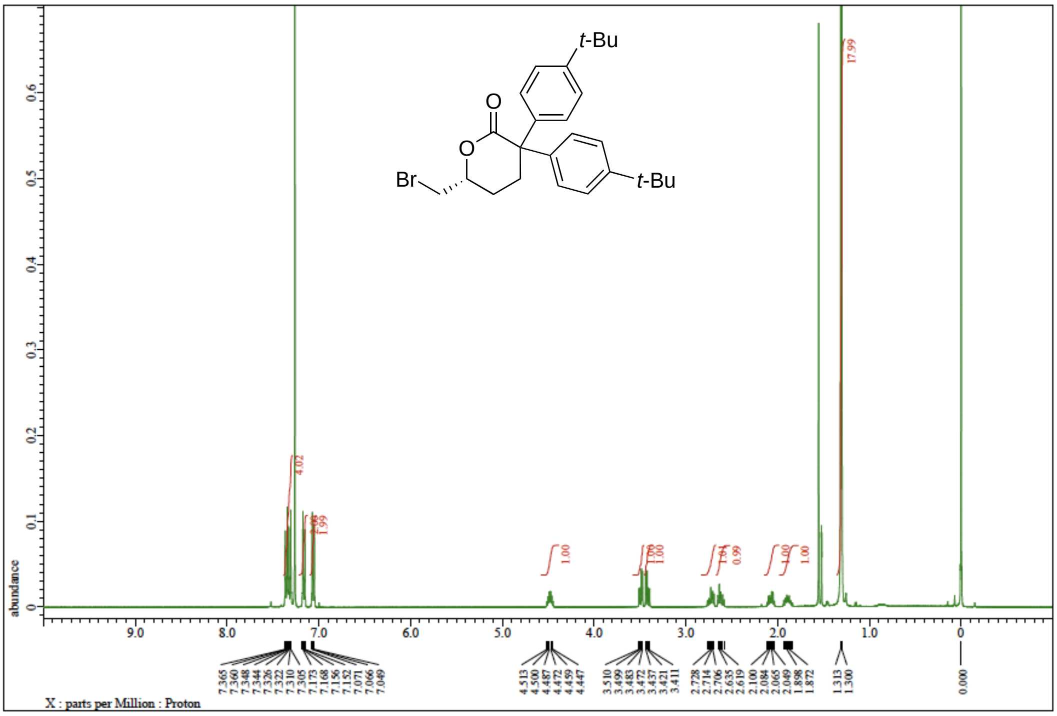

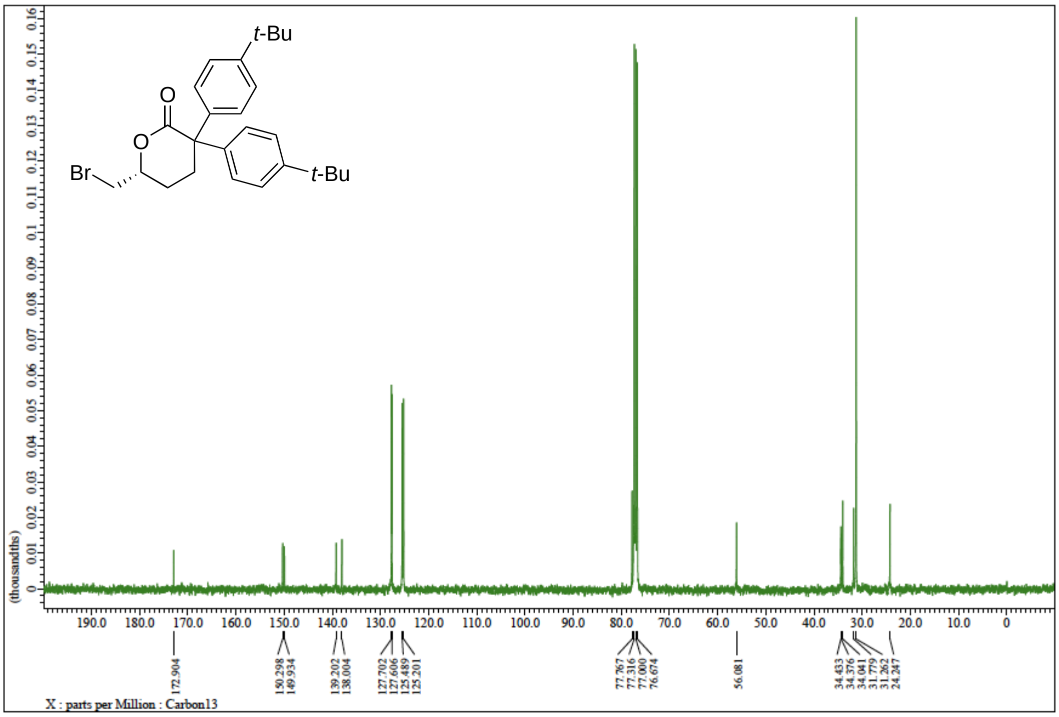

**3e**: $^{1}H$ and $^{13}C$ NMR

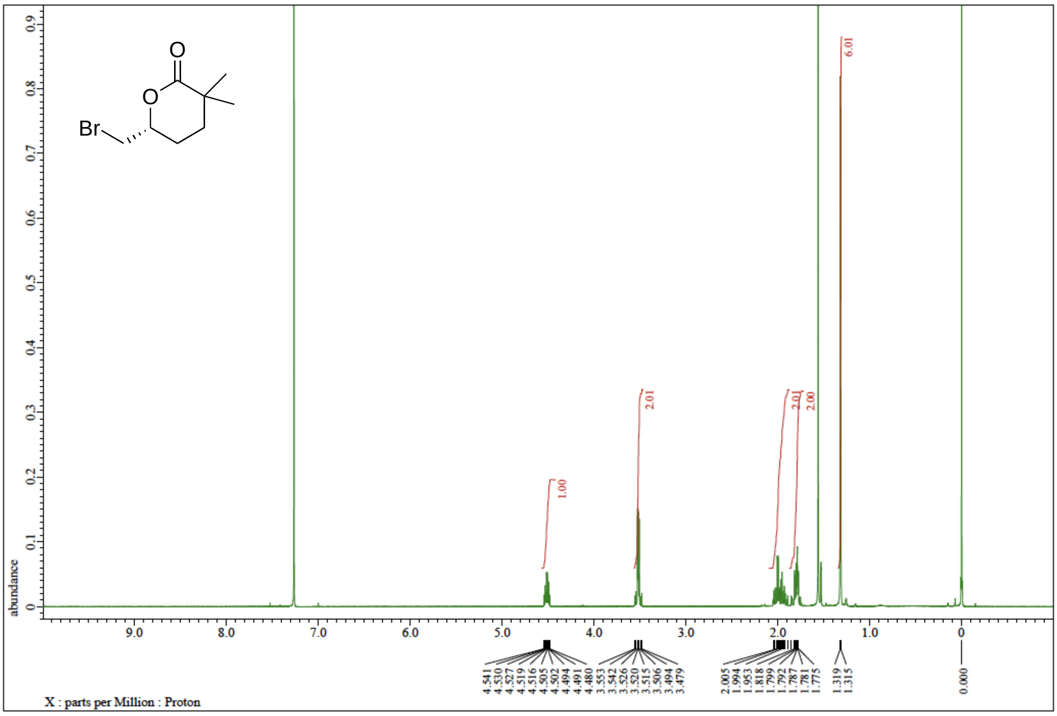


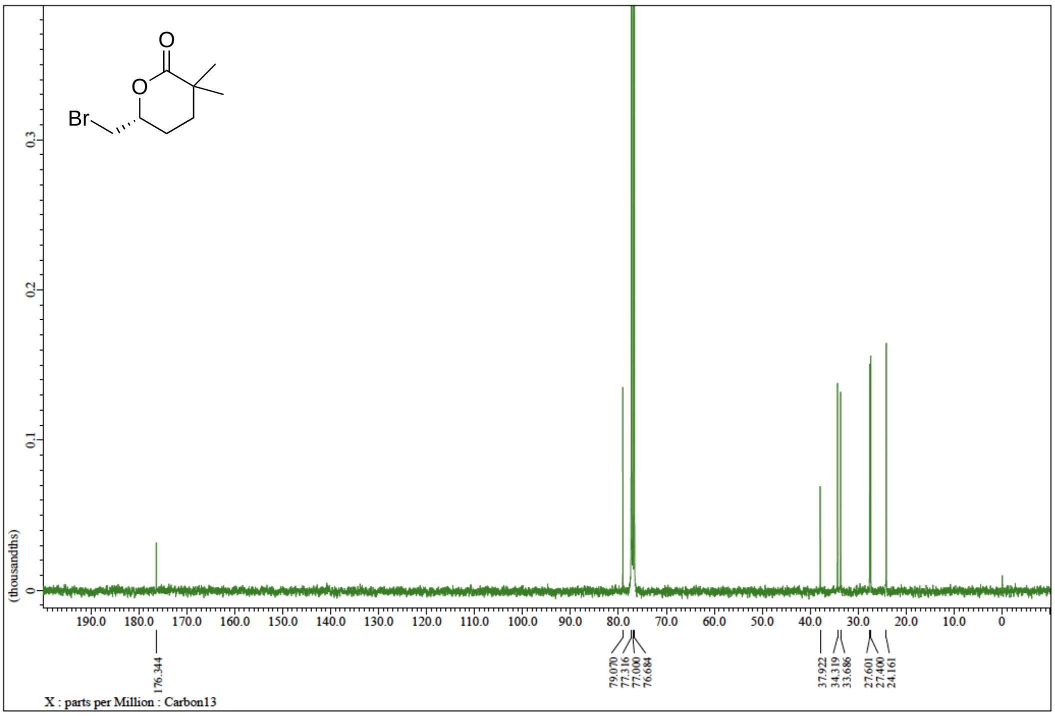

**3f**: $^{1}H$ and $^{13}C$ NMR

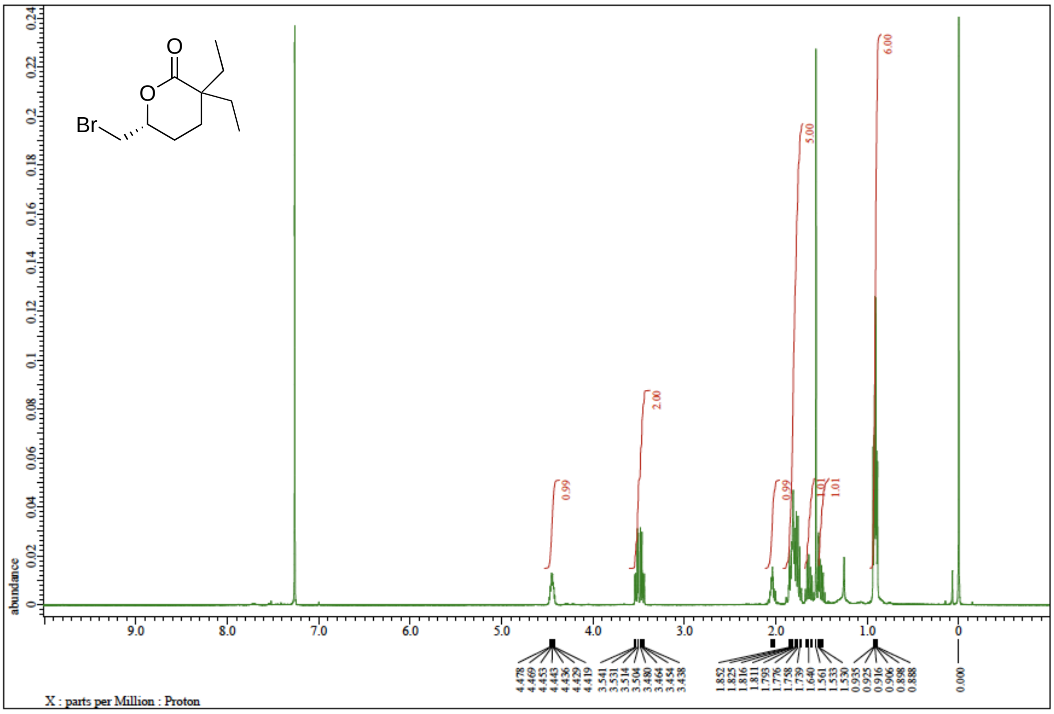


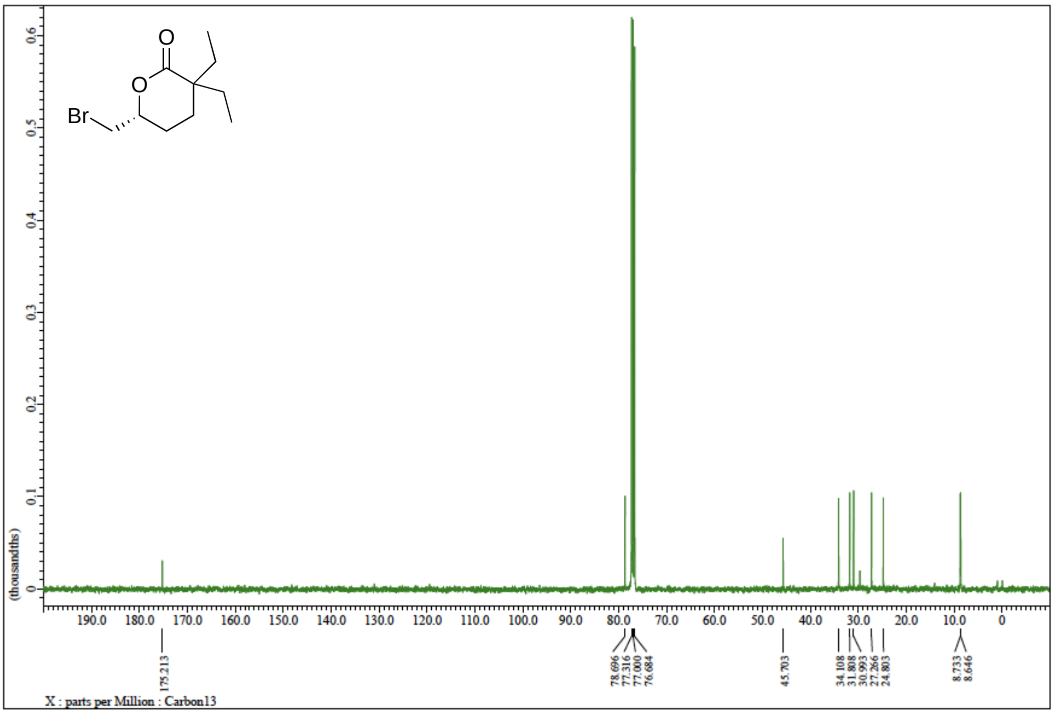

**3g**: $^{1}H$ and $^{13}C$ NMR

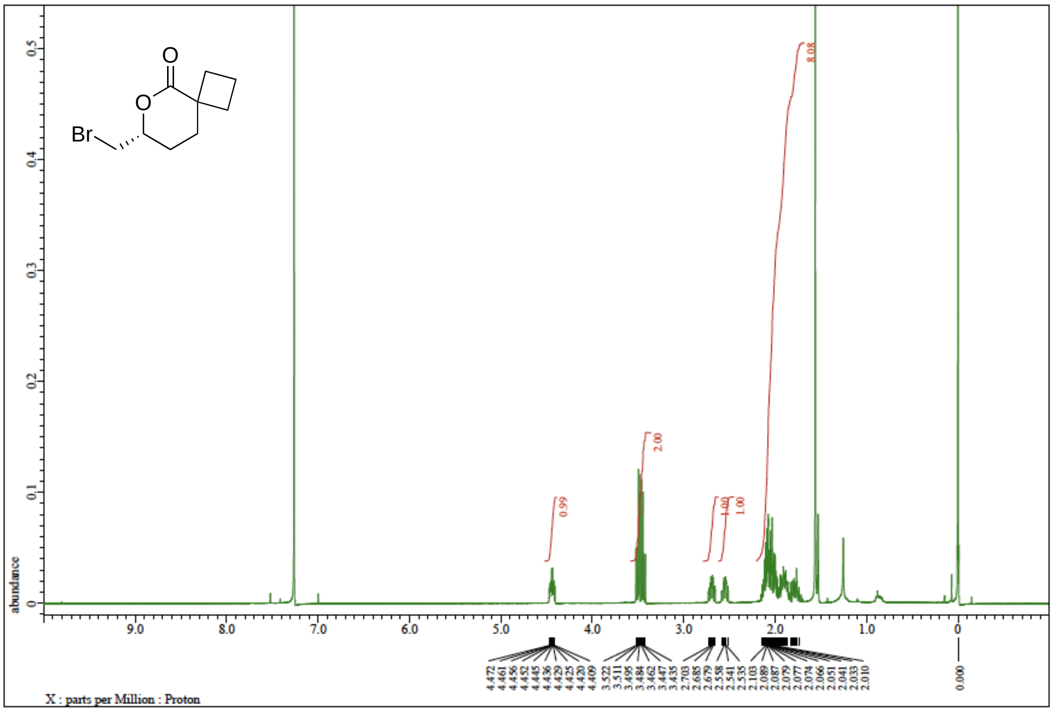

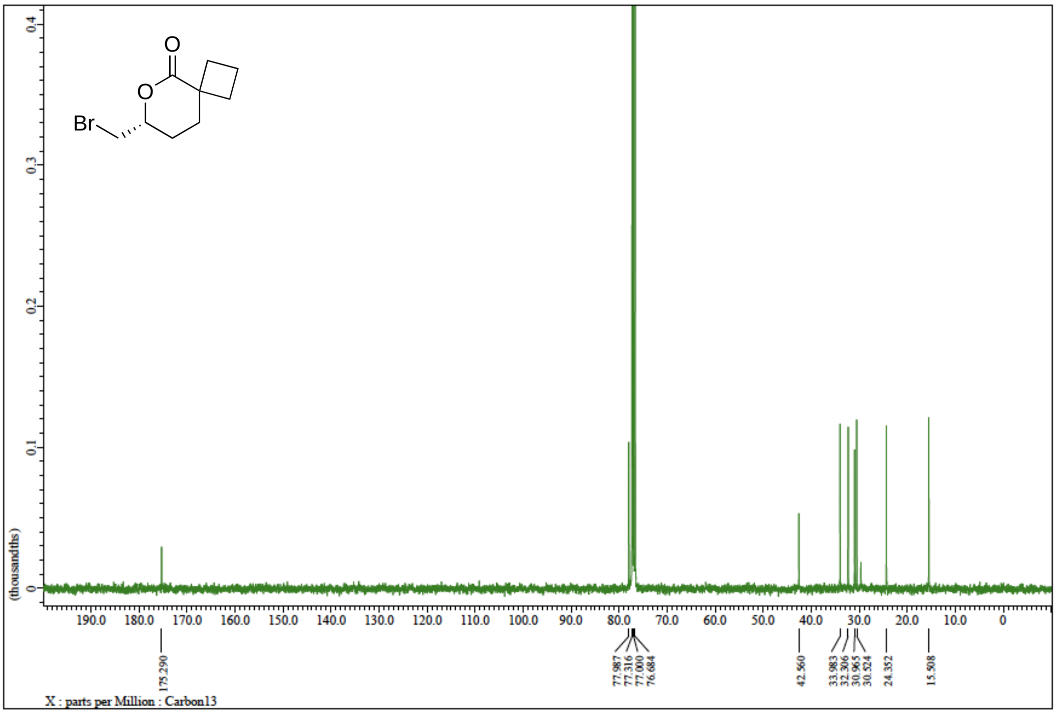

**3h**: $^{1}$H and $^{13}$C NMR

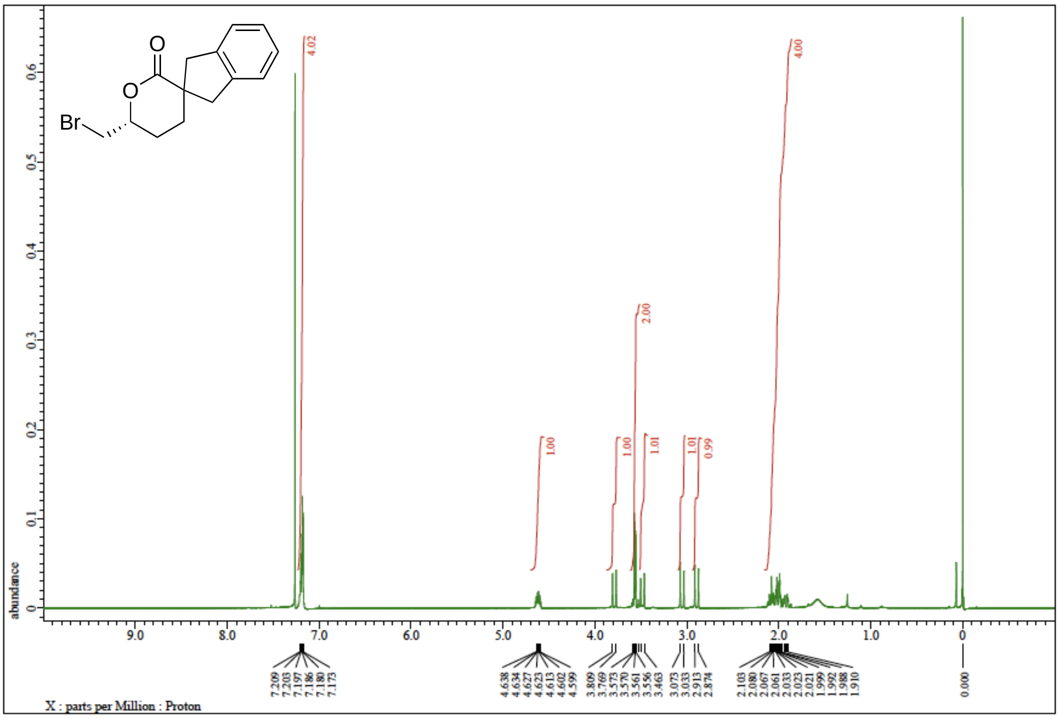

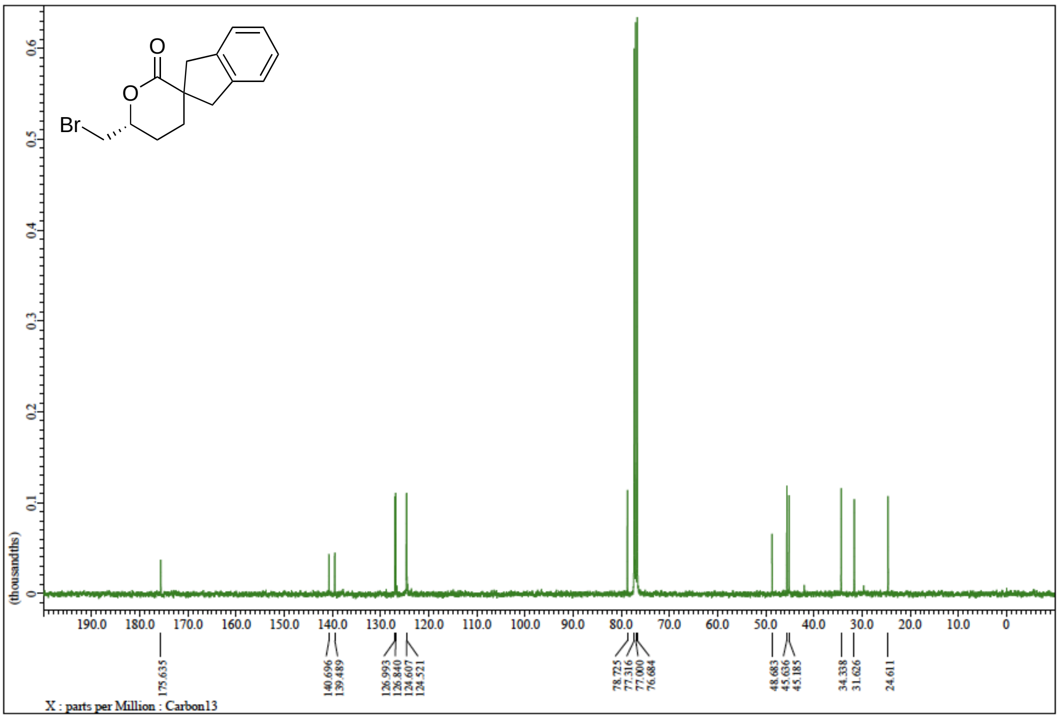

**3i**: $^{1}H$ and $^{13}C$ NMR

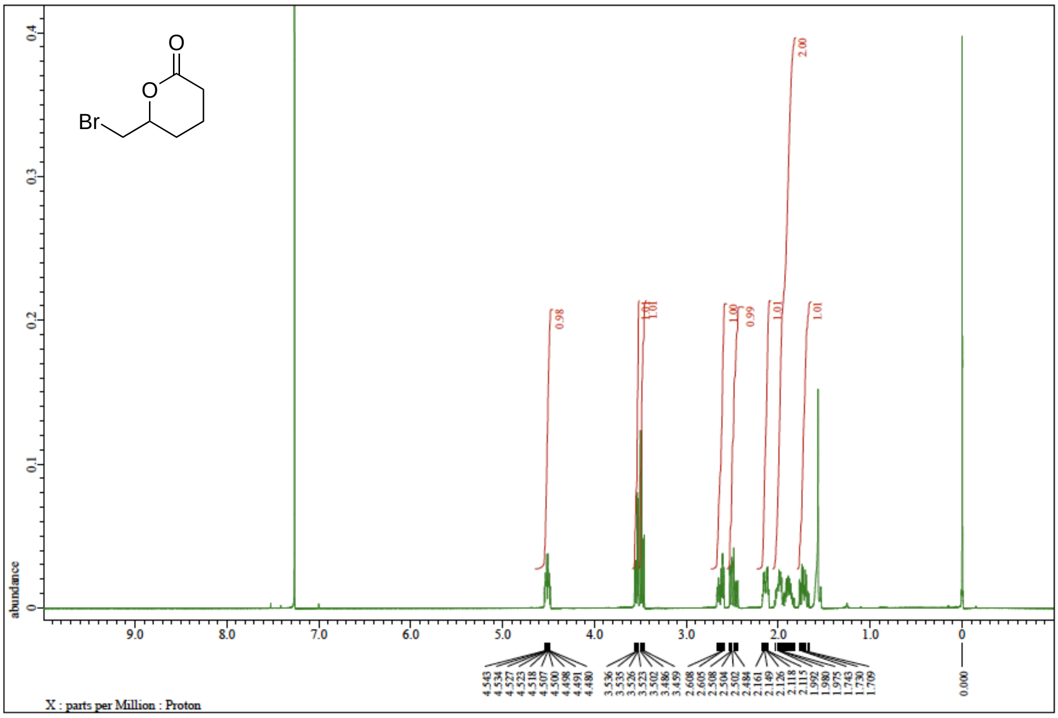


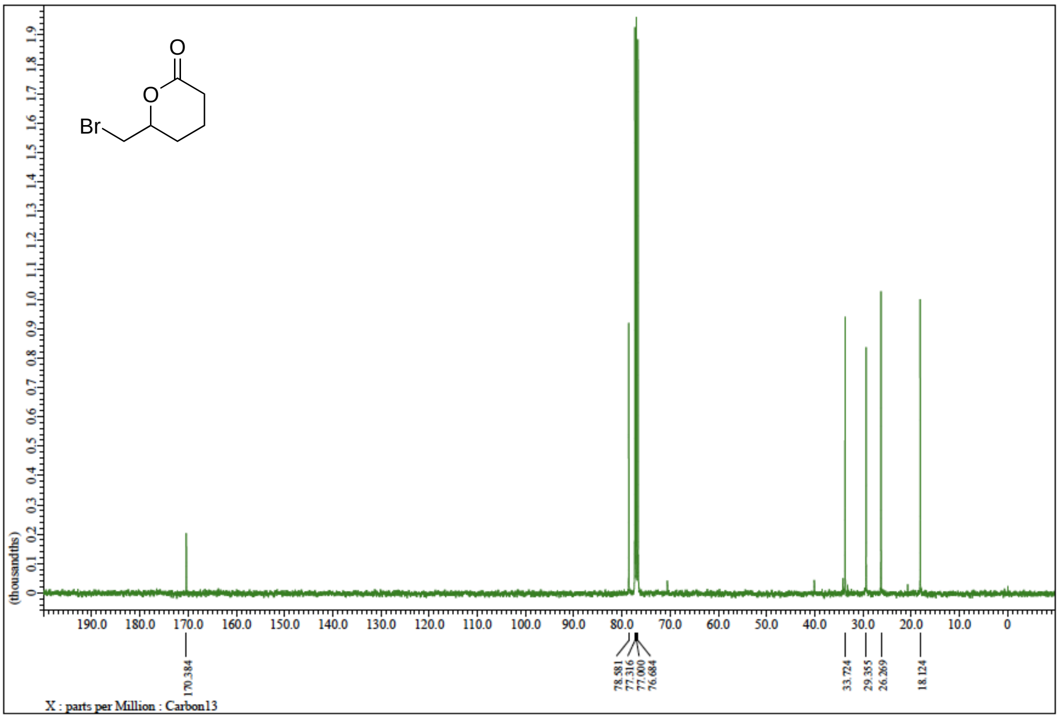

**3j**: $^{1}$H and $^{13}$C NMR

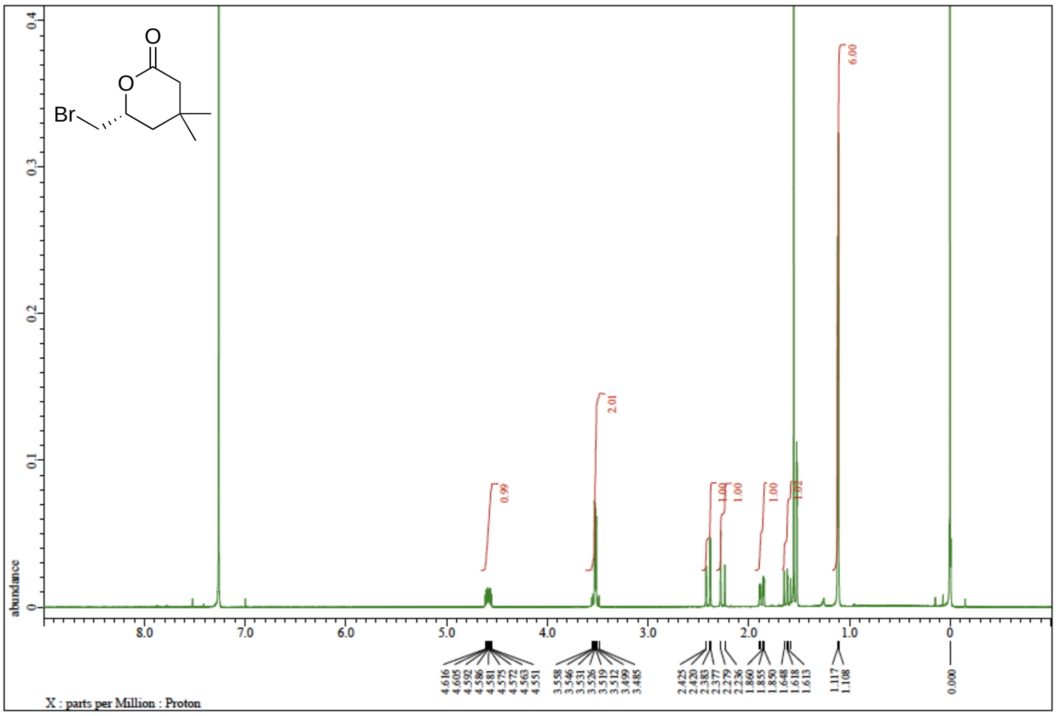


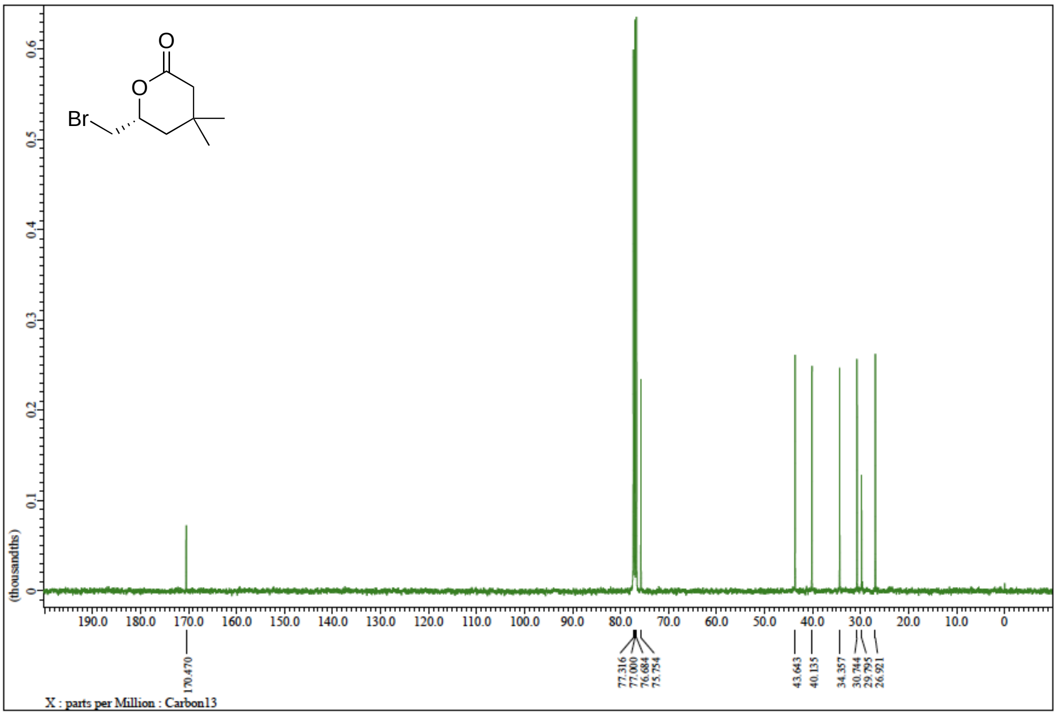

**3k**: $^{1}H$ and $^{13}C$ NMR

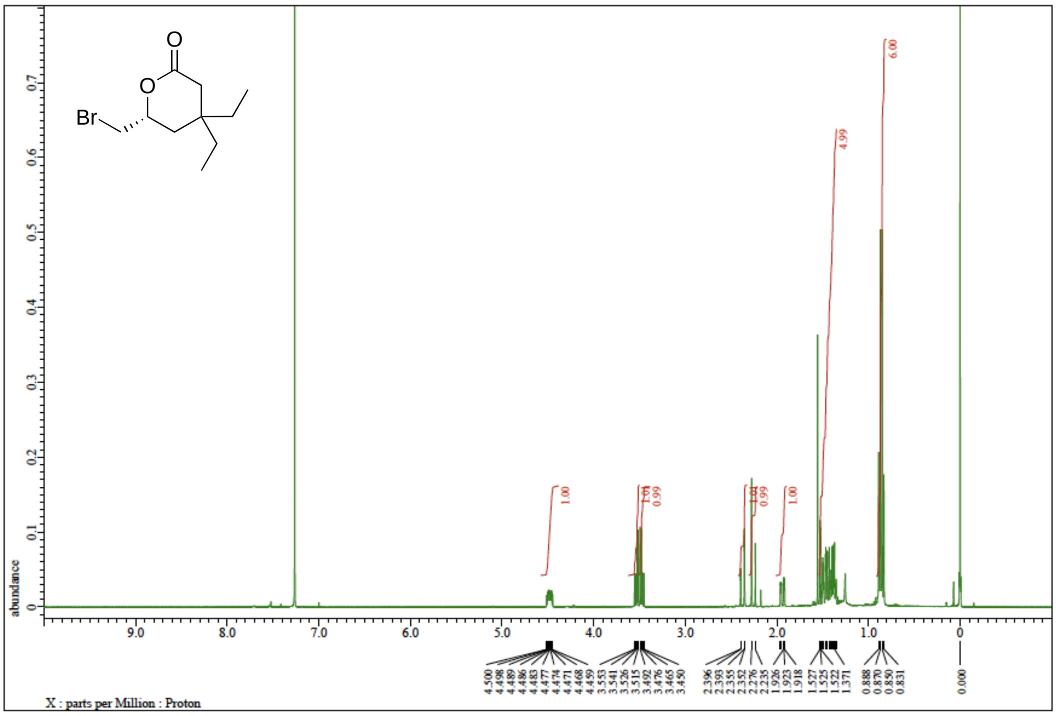

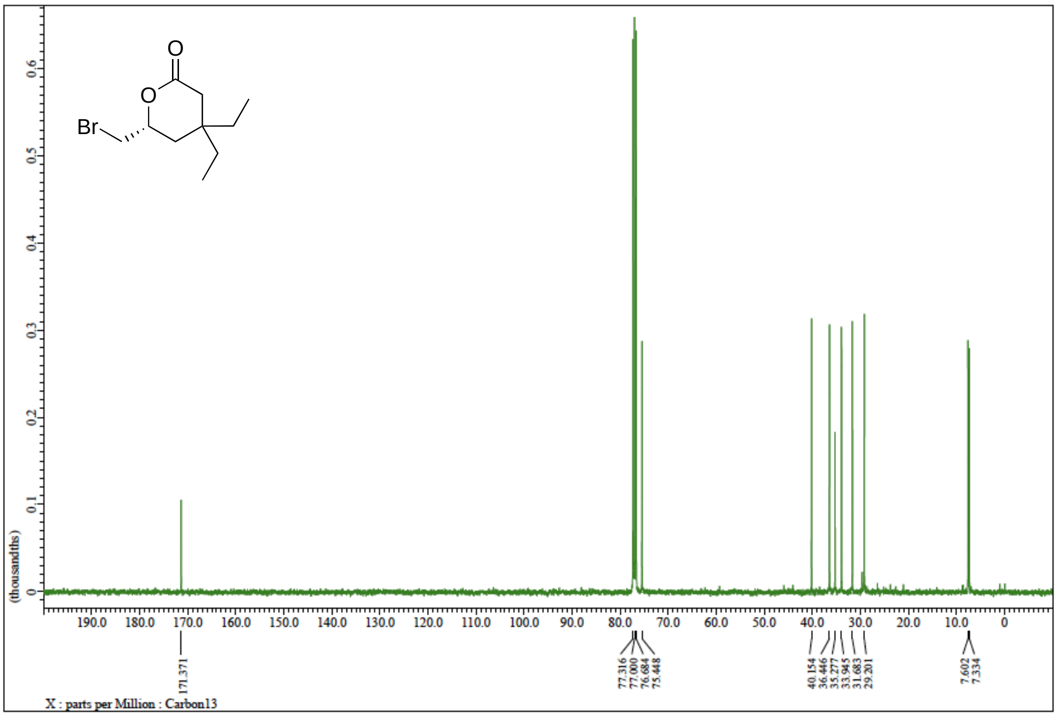

**3l**: $^{1}$H and $^{13}$C NMR

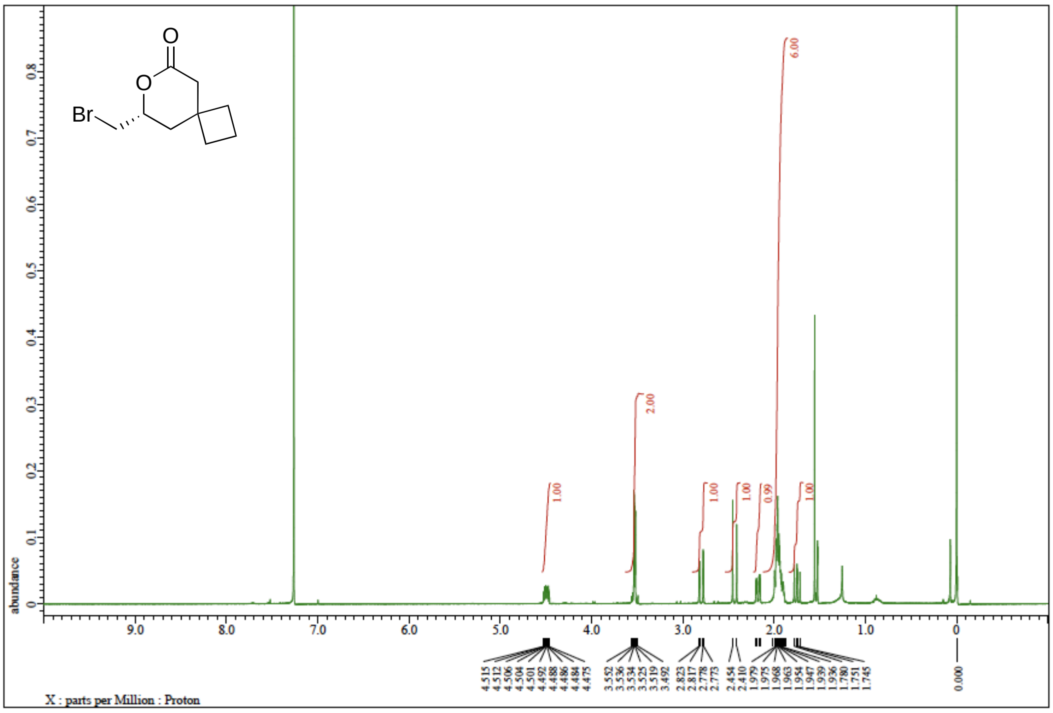

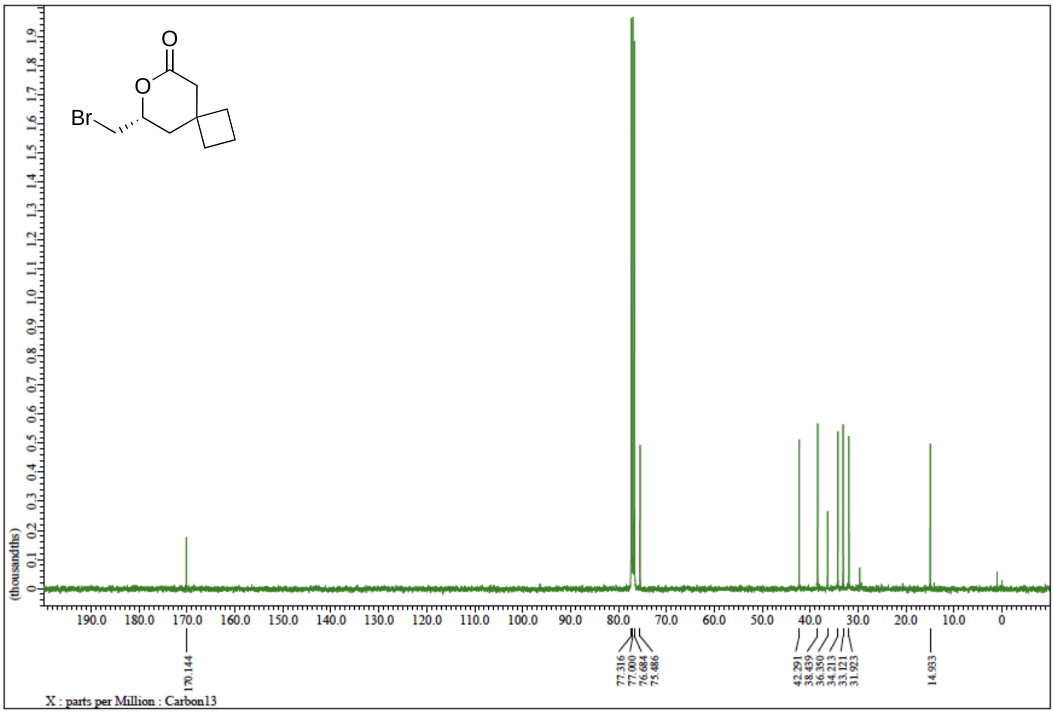

**3m**: $^{1}$H and $^{13}$C NMR

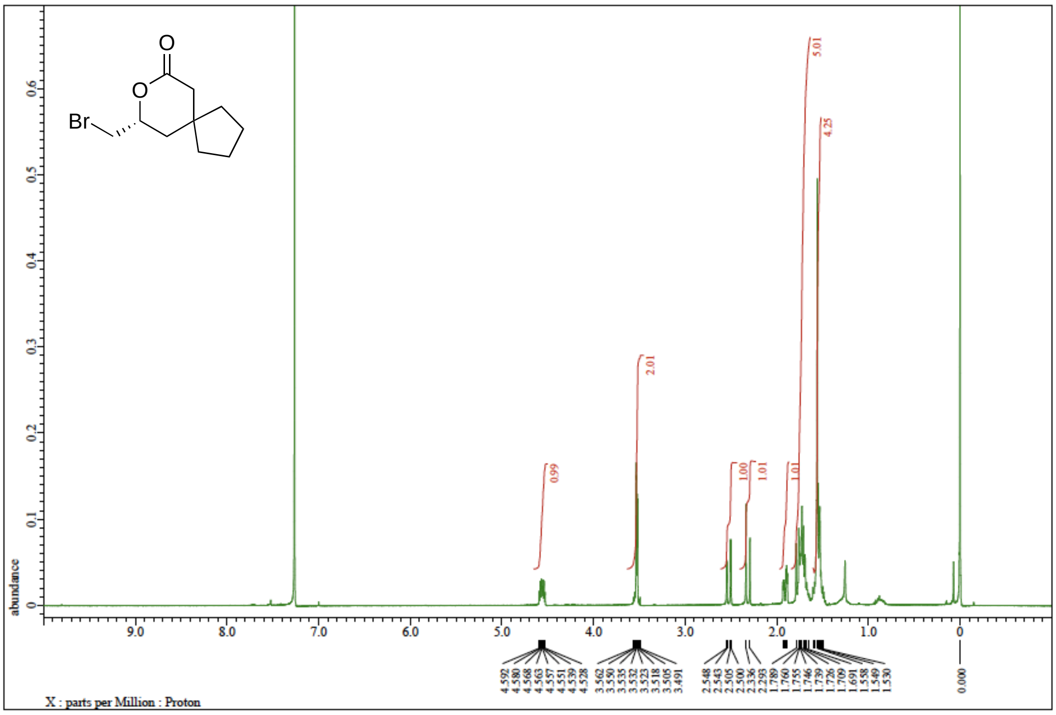


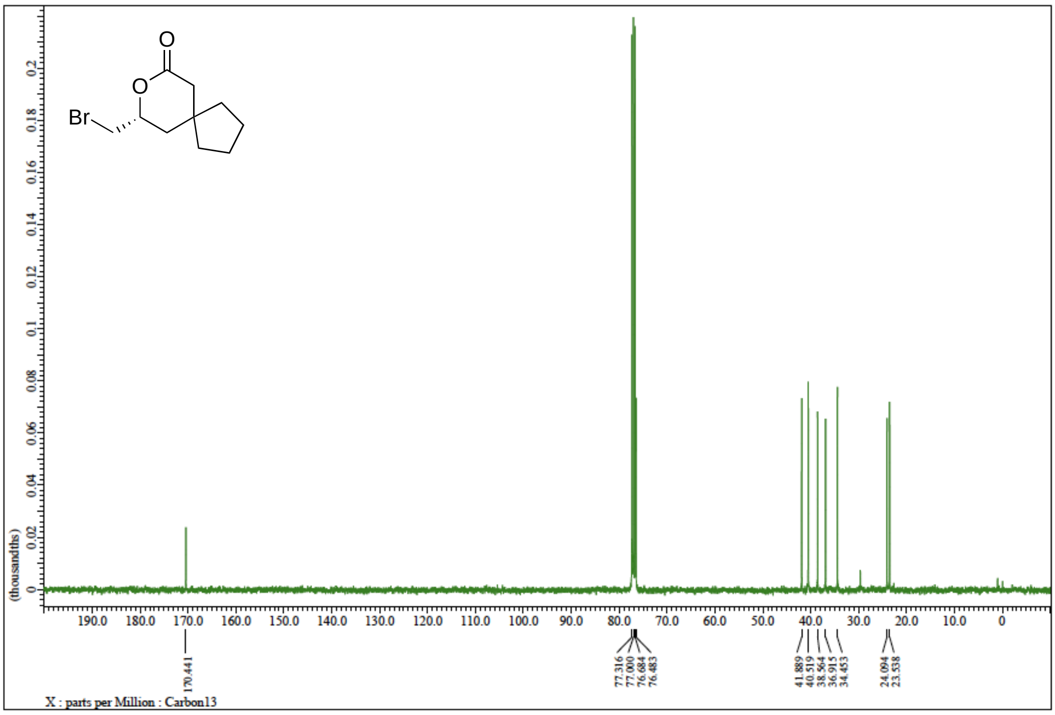

**3n**: $^{1}$H and $^{13}$C NMR

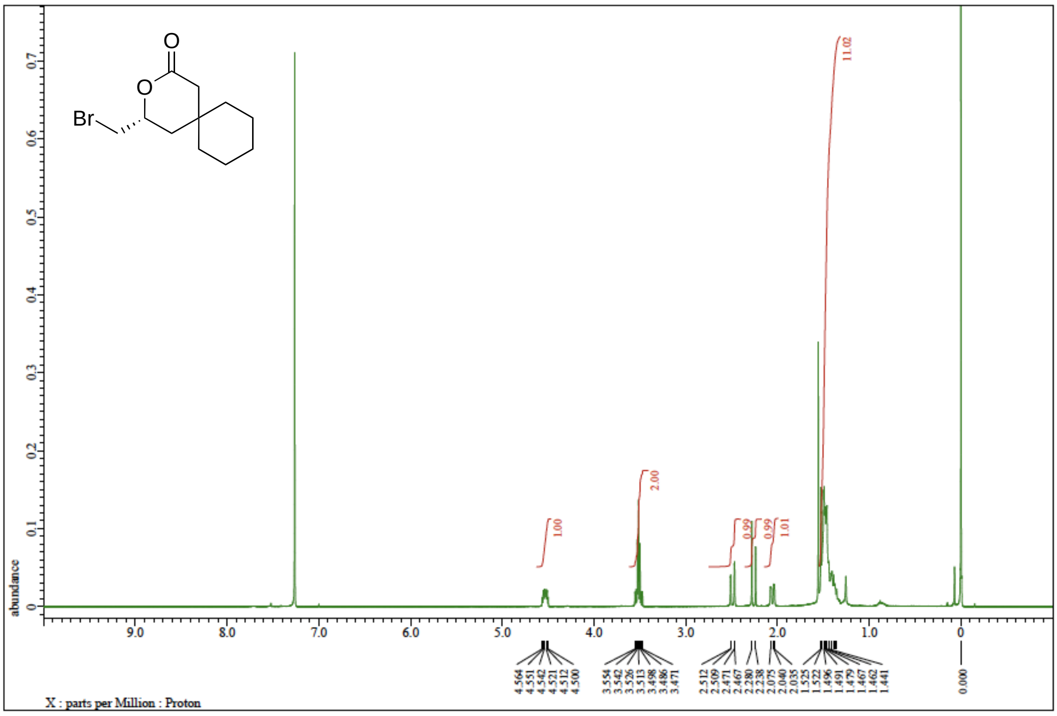


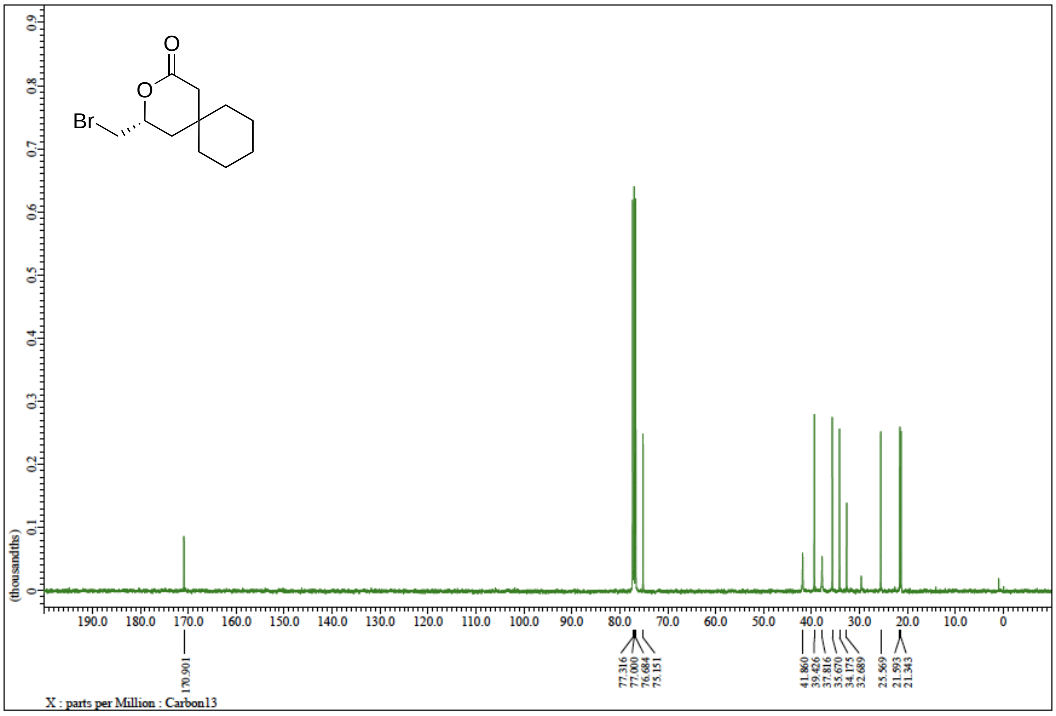

**3o**: $^{1}$H and $^{13}$C NMR

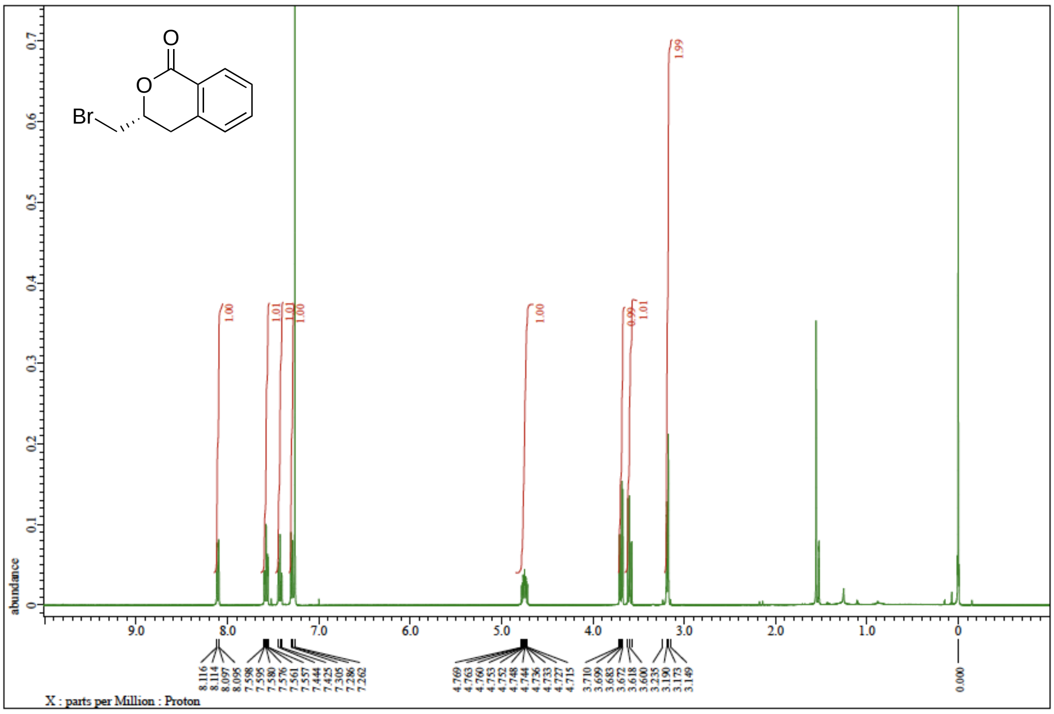

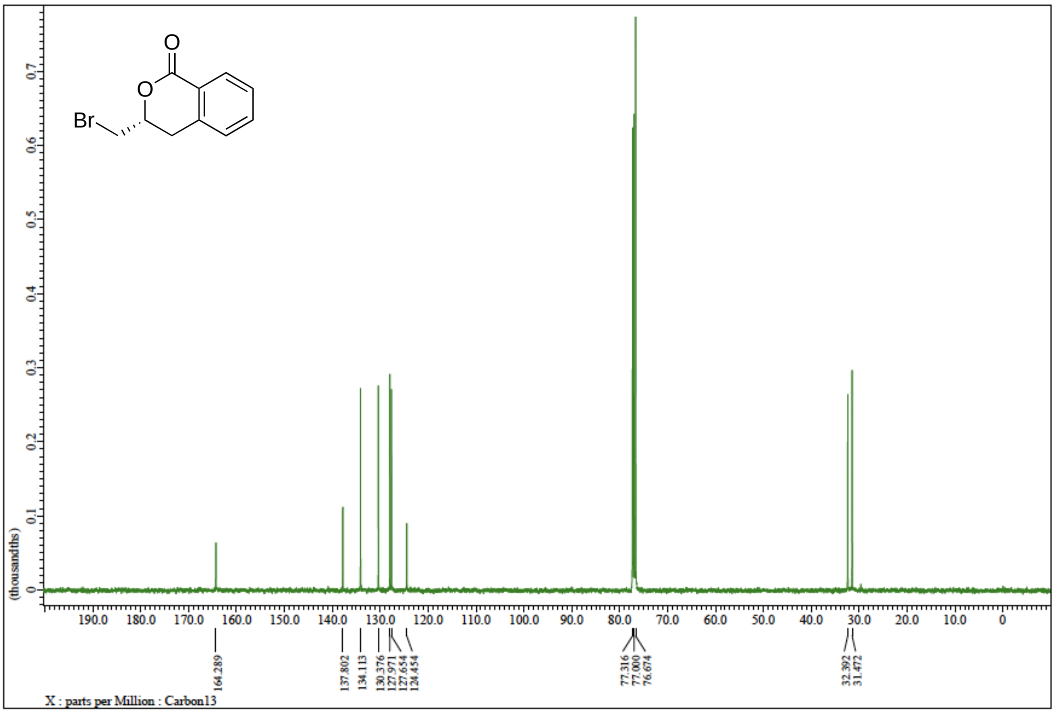

**7**: $^{1}H$ and $^{13}C$ NMR

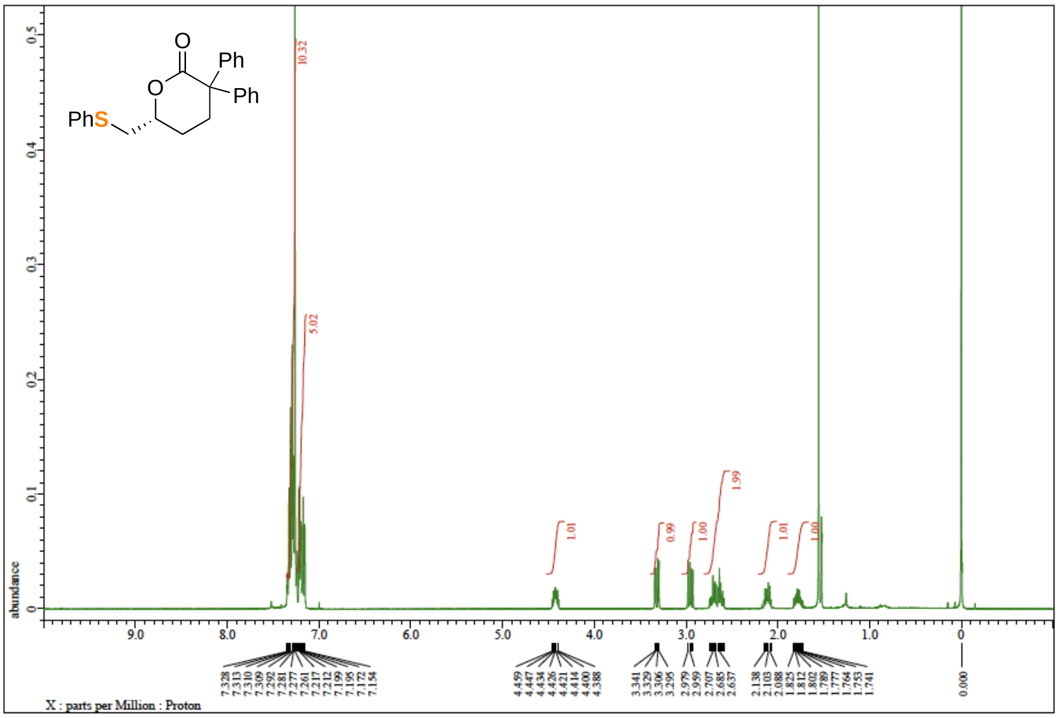


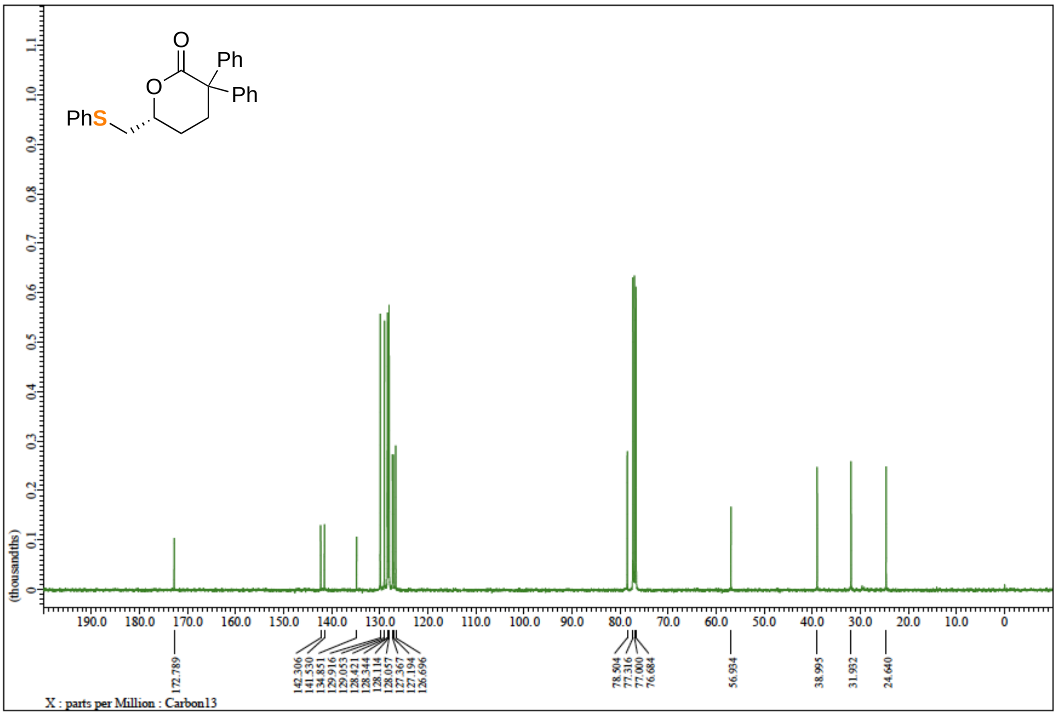

**8**: $^{1}$H and $^{13}$C NMR

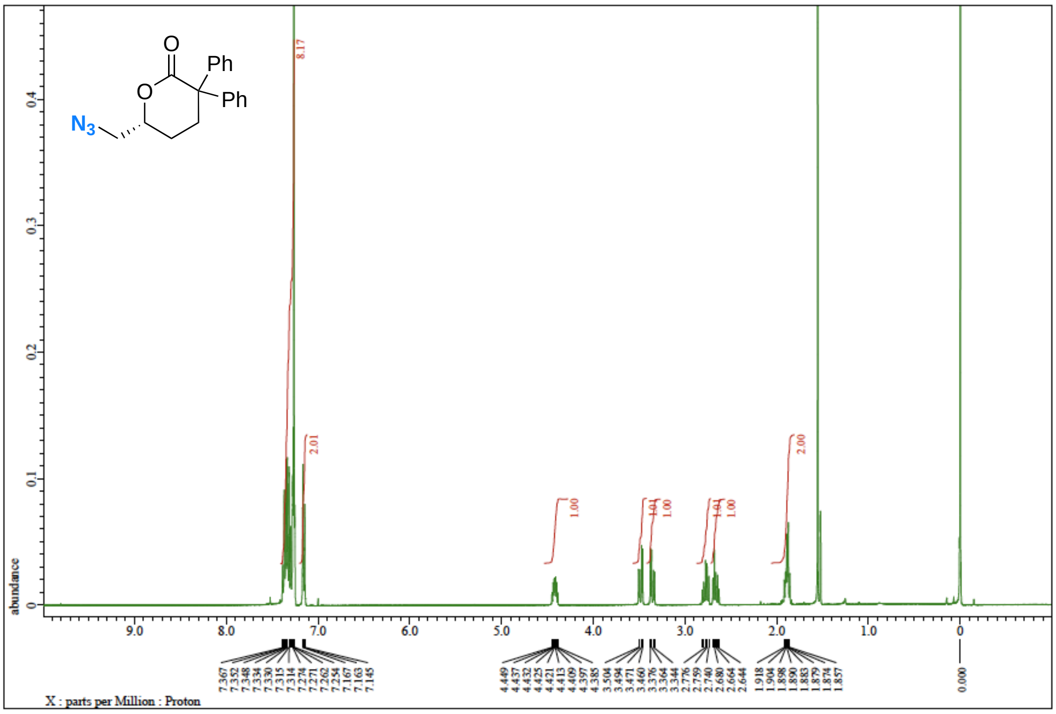

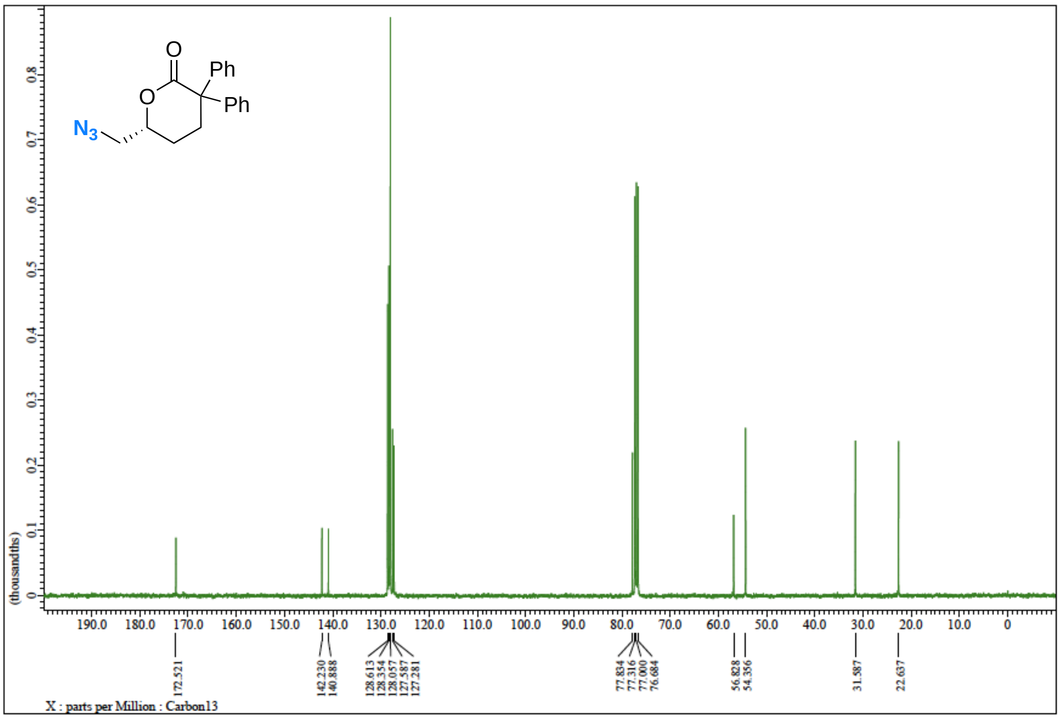

**9**: $^{1}$H and $^{13}$C NMR

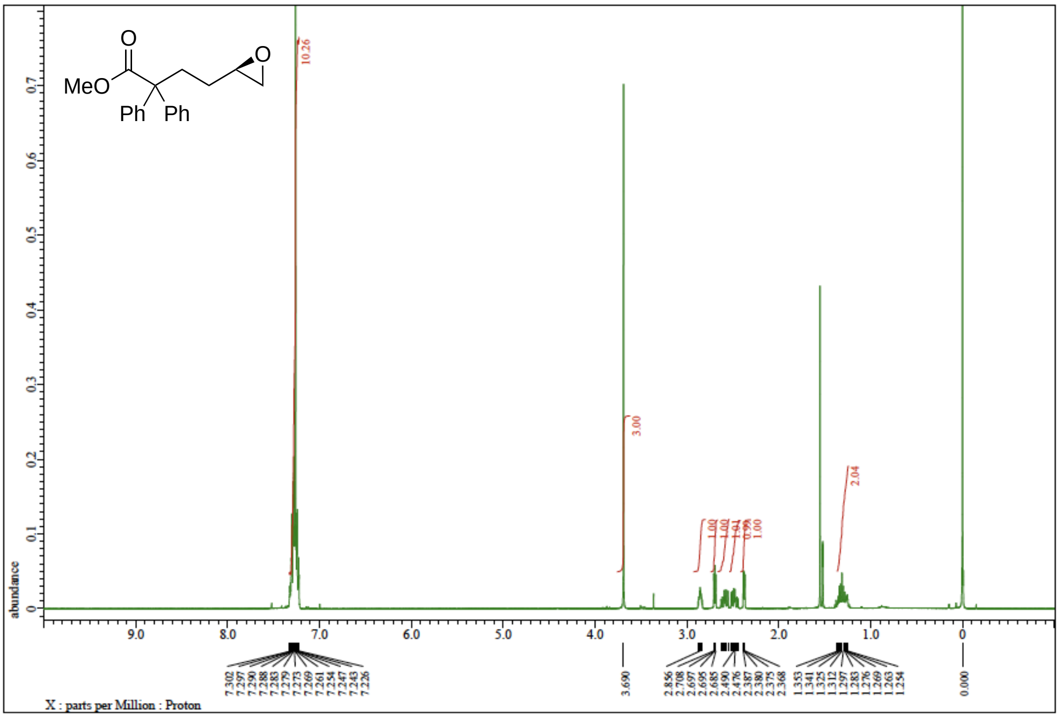

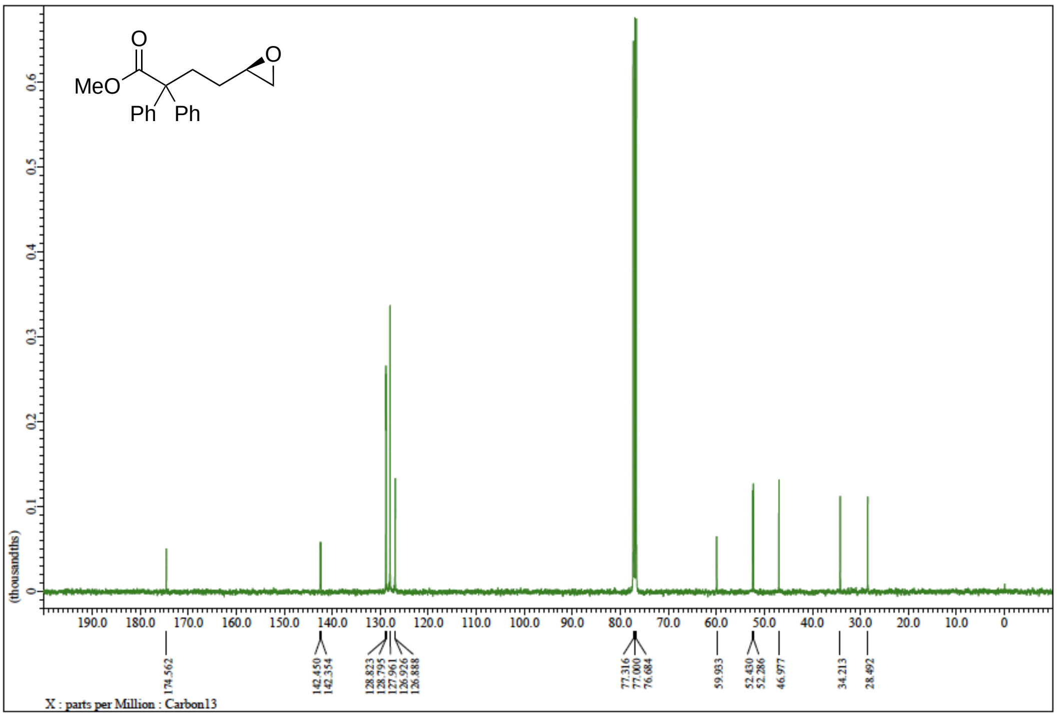

## HPLC Charts

**3a**: Daicel Chiralpak IE-3, hexane/2-propanol = 2:1, flow rate = 0.5 mL/min, 254 nm

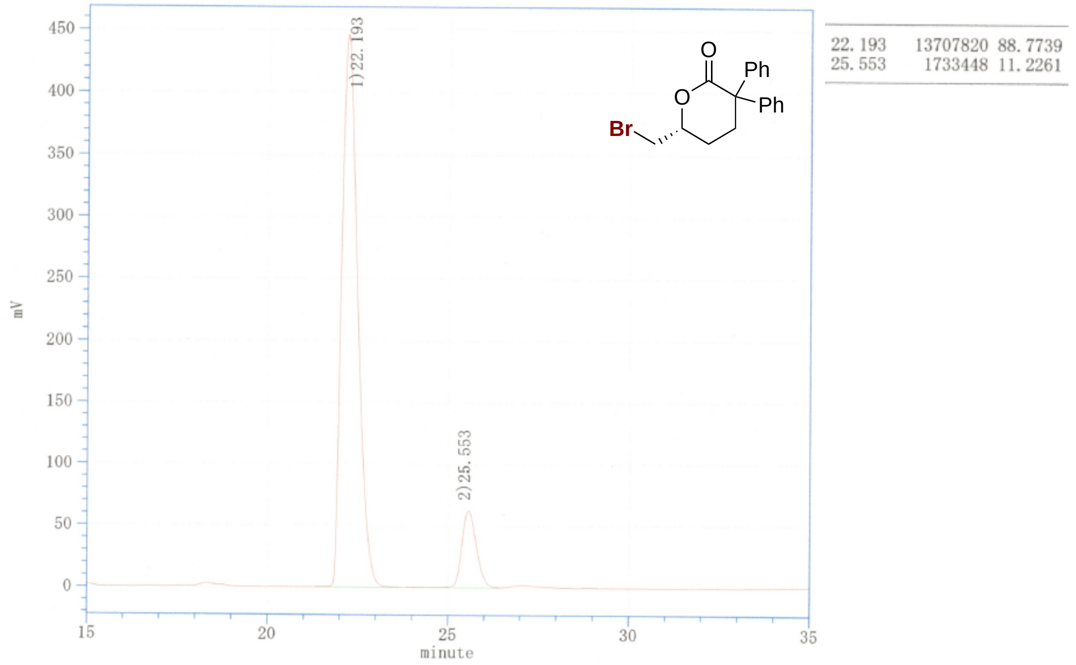


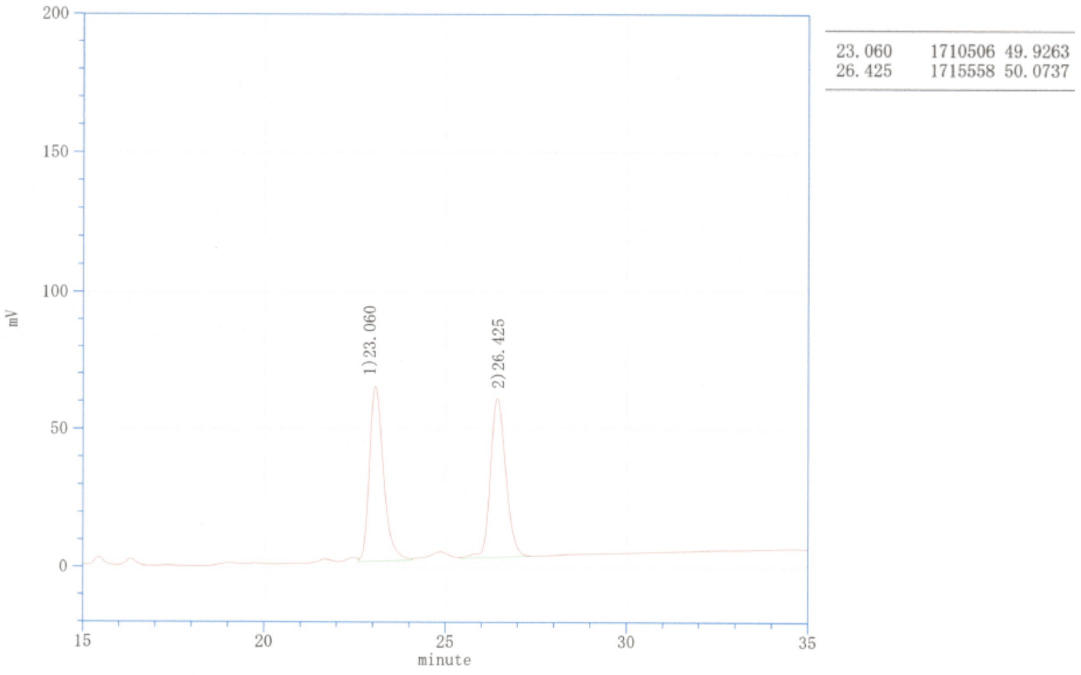

**Iodolactonization Product**: Daicel Chiralpak IE-3, hexane/2-propanol = 2:1, flow rate = 0.5 mL/min, 254 nm

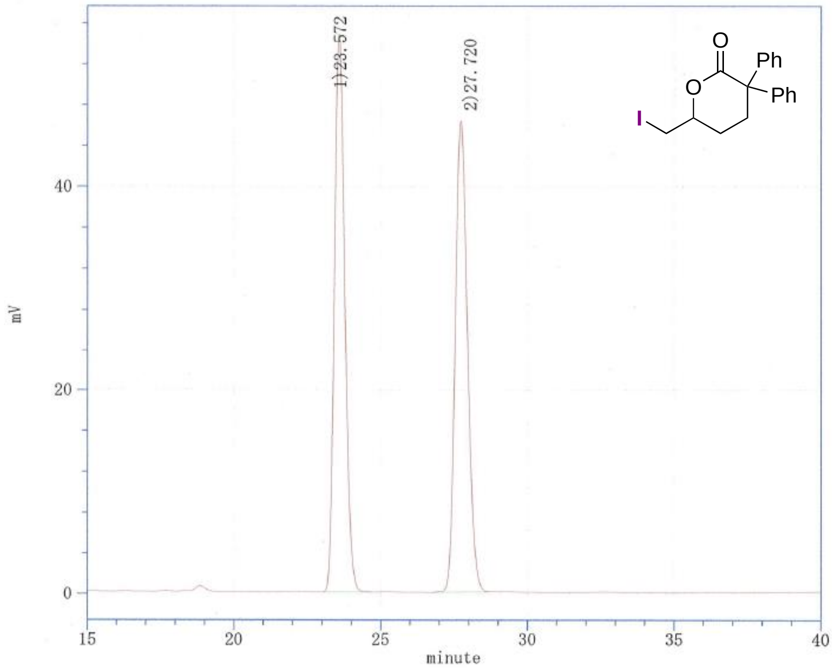


| | | |
|---|---|---|
| 23.572 | 1347435 | 50.1752 |
| 27.720 | 1338025 | 49.8248 |

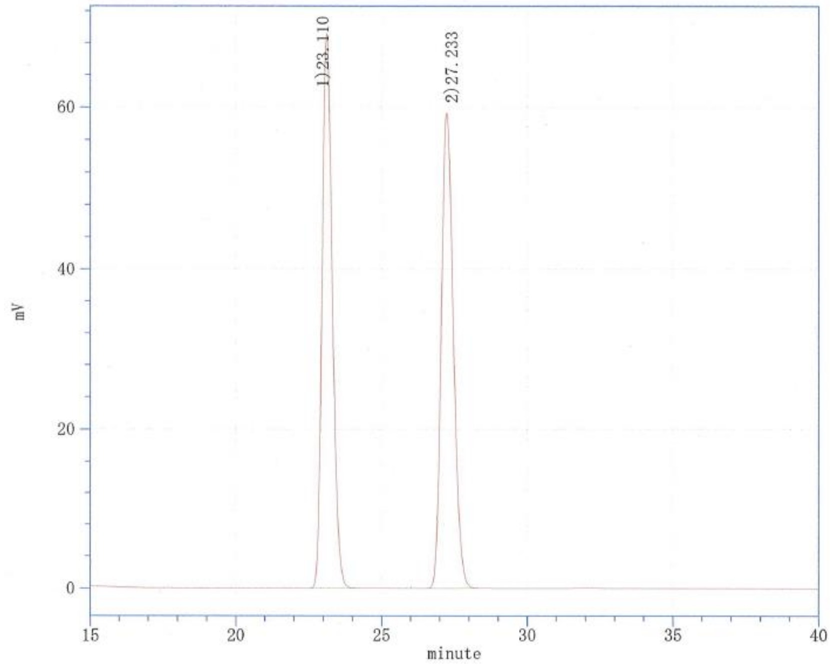


| | | |
|---|---|---|
| 23.110 | 1708735 | 50.0166 |
| 27.233 | 1707603 | 49.9834 |

**3b**: Daicel Chiralpak IC-3, hexane/2-propanol = 2:1, flow rate = 0.5 mL/min, 254 nm

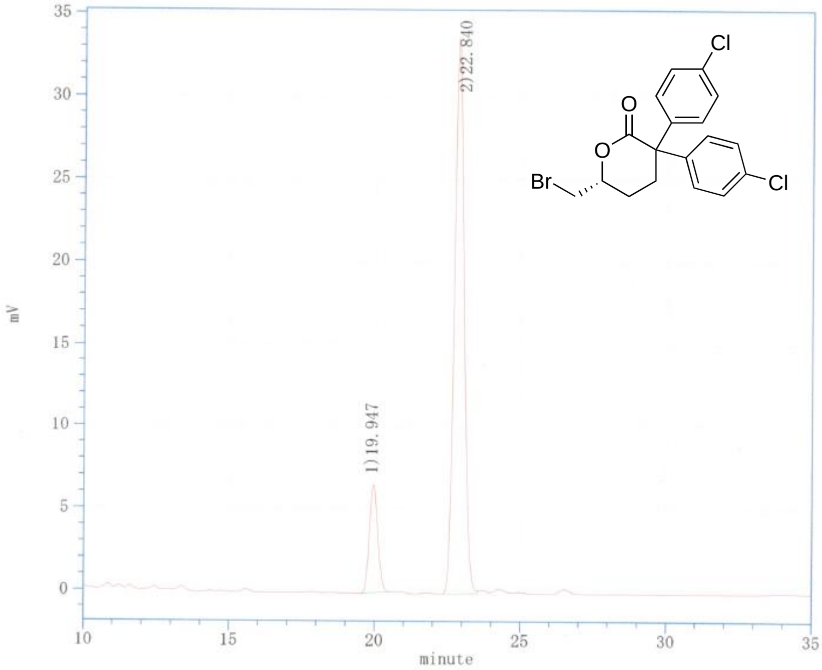


| | | |
|---|---|---|
| 19.947 | 131440 | 14.4174 |
| 22.840 | 780238 | 85.5826 |

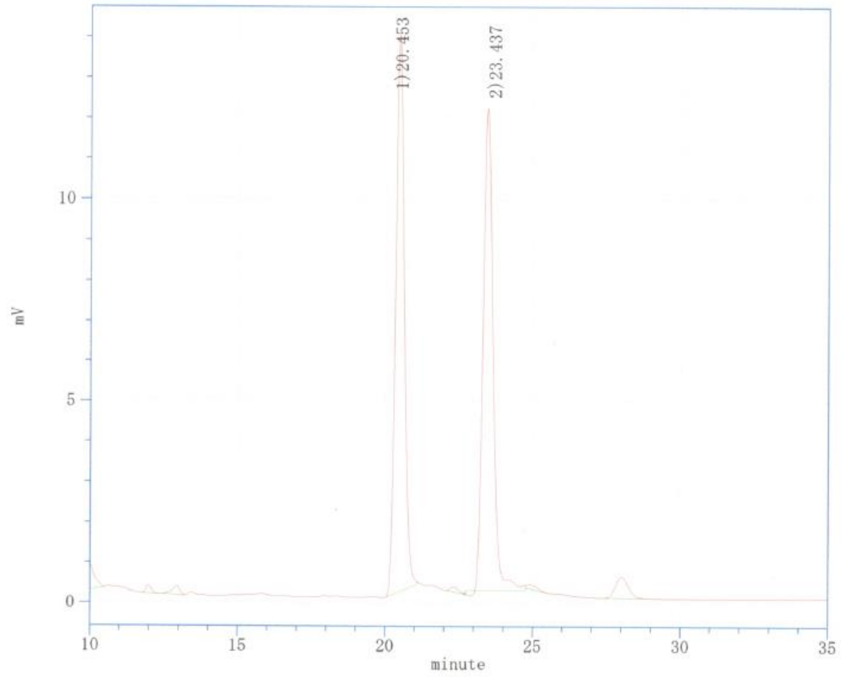


| | | |
|---|---|---|
| 20.453 | 282650 | 50.1116 |
| 23.437 | 281391 | 49.8884 |

**3c**: Daicel Chiralpak IE-3, hexane/2-propanol = 2:1, flow rate = 0.5 mL/min, 254 nm

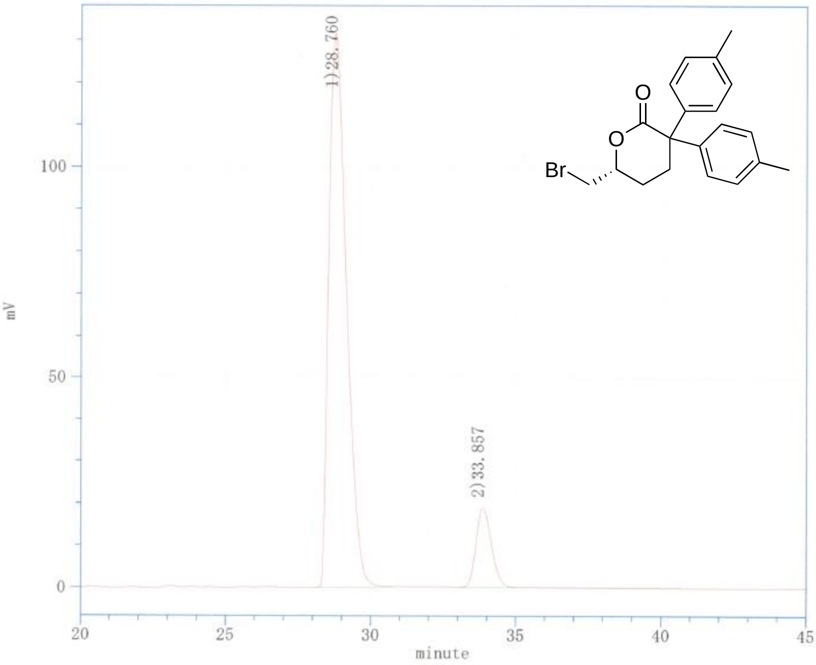


| | | |
|---|---|---|
| 28.760 | 5572493 | 88.9717 |
| 33.857 | 690729 | 11.0283 |

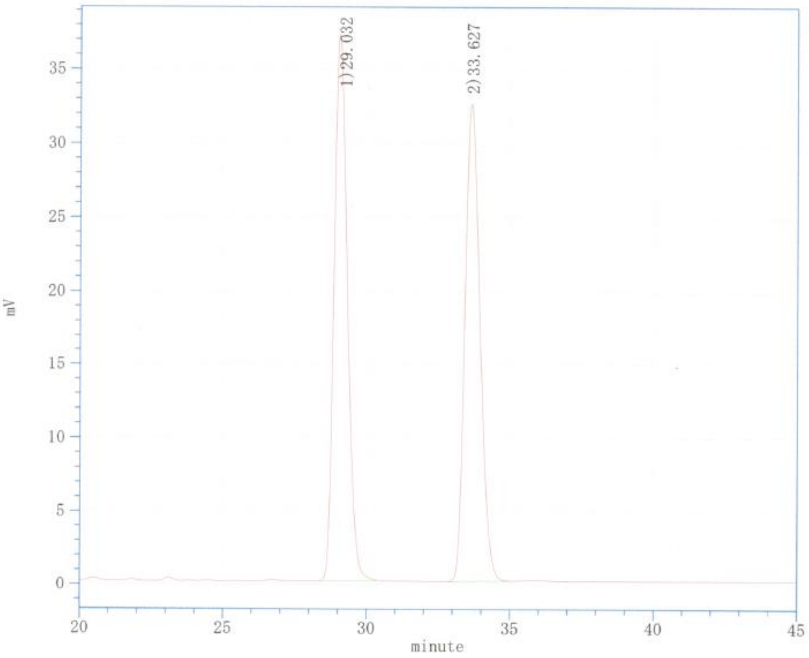


| | | |
|---|---|---|
| 29.032 | 1194906 | 50.2229 |
| 33.627 | 1184301 | 49.7771 |

**3d**: Daicel Chiralpak IE-3, hexane/2-propanol = 5:1, flow rate = 0.5 mL/min, 254 nm

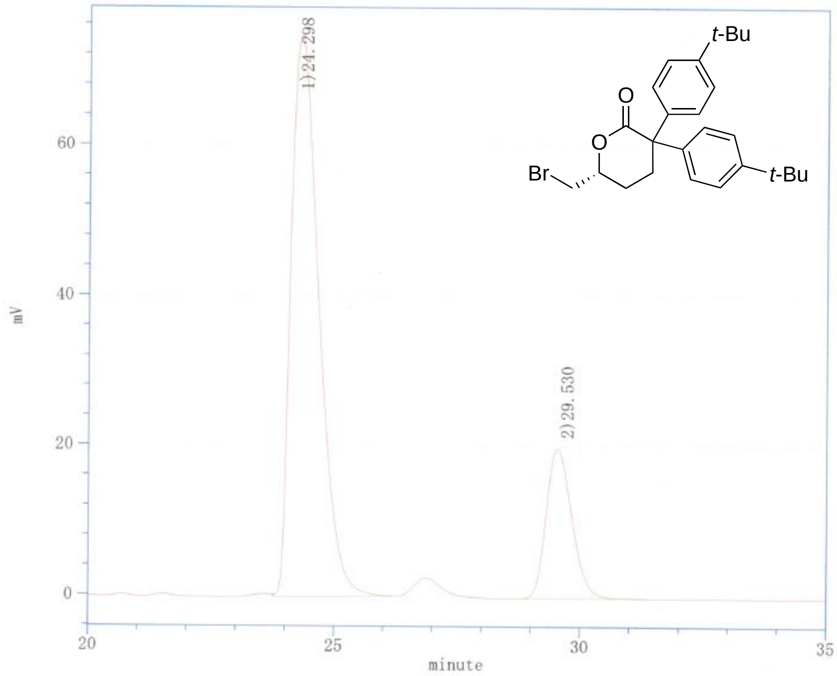


| | | |
|---|---|---|
| 24.298 | 2943890 | 80.2351 |
| 29.530 | 725188 | 19.7649 |

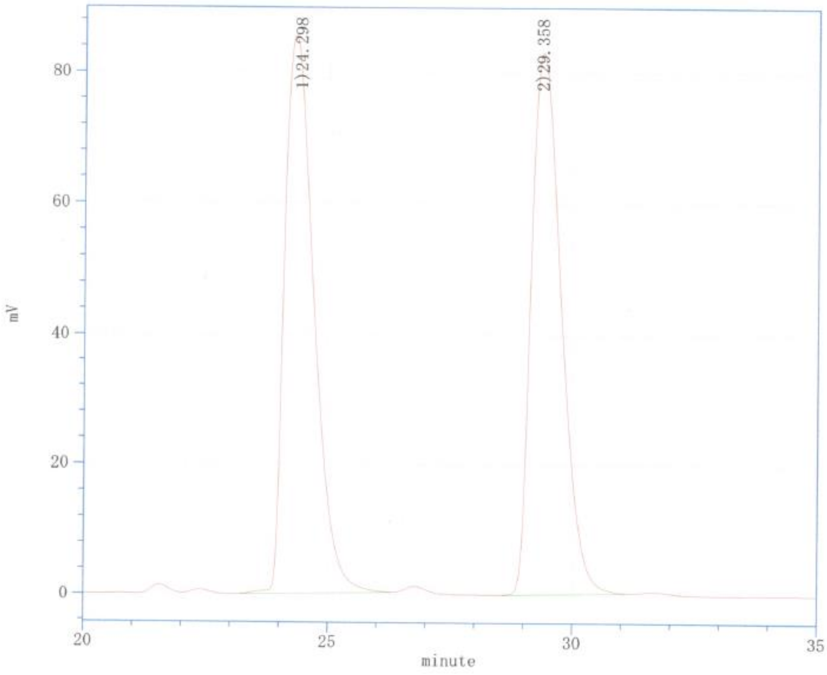


| | | |
|---|---|---|
| 24.298 | 3571091 | 50.1734 |
| 29.358 | 3546407 | 49.8266 |

**3e**: Daicel Chiralpak IC-3, hexane/2-propanol = 3:1, flow rate = 0.5 mL/min, 214 nm

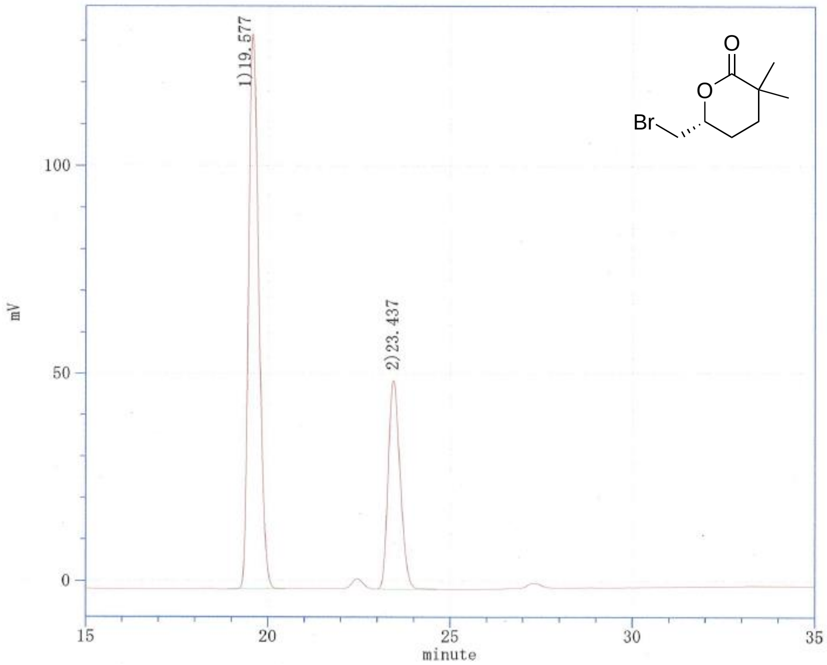


| | | |
|---|---|---|
| 19.577 | 2607105 | 69.4690 |
| 23.437 | 1145798 | 30.5310 |

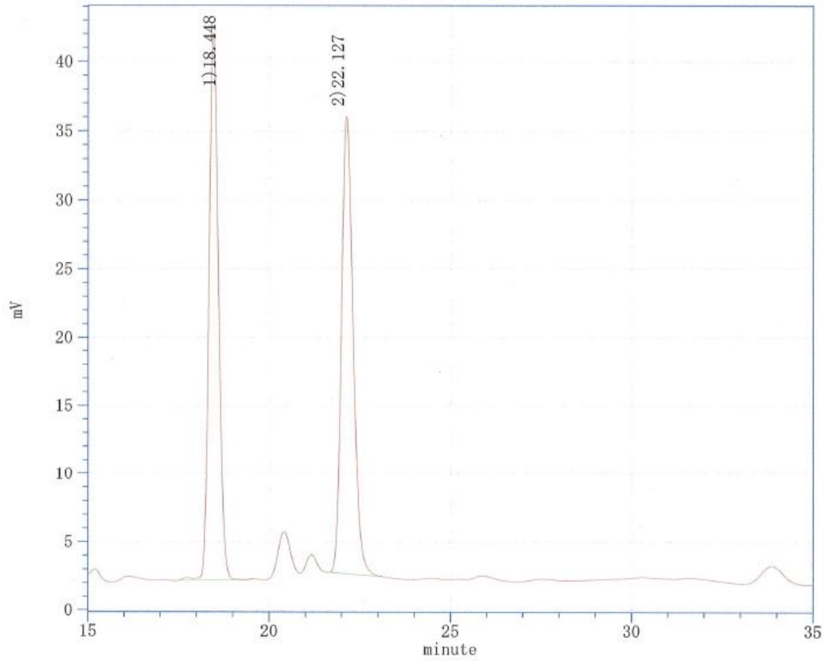


| | | |
|---|---|---|
| 18.448 | 730965 | 49.5018 |
| 22.127 | 745678 | 50.4982 |

**3f**: Daicel Chiralpak IC-3, hexane/2-propanol = 10:1, flow rate = 0.5 mL/min, 214 nm

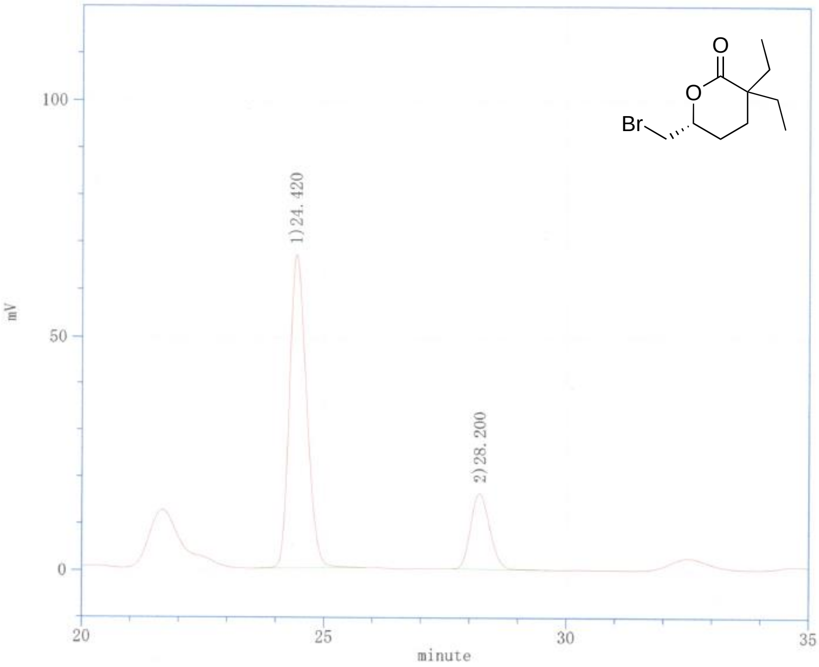


| | | |
|---|---|---|
| 24.420 | 1637718 | 78.8689 |
| 28.200 | 438788 | 21.1311 |

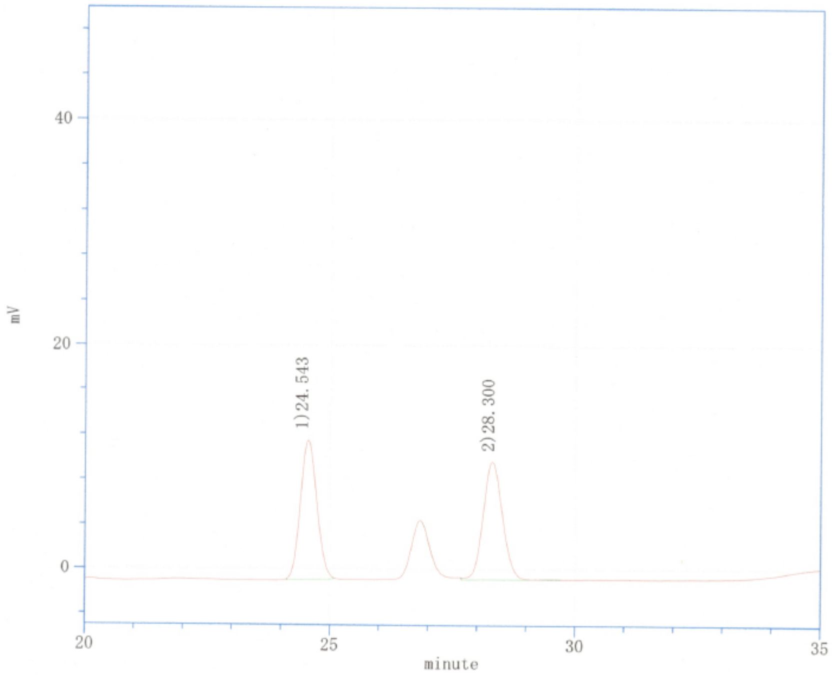


| | | |
|---|---|---|
| 24.543 | 291307 | 50.1254 |
| 28.300 | 289849 | 49.8746 |

**3g**: Daicel Chiralpak IC-3, hexane/2-propanol = 10:1, flow rate = 0.5 mL/min, 254 nm

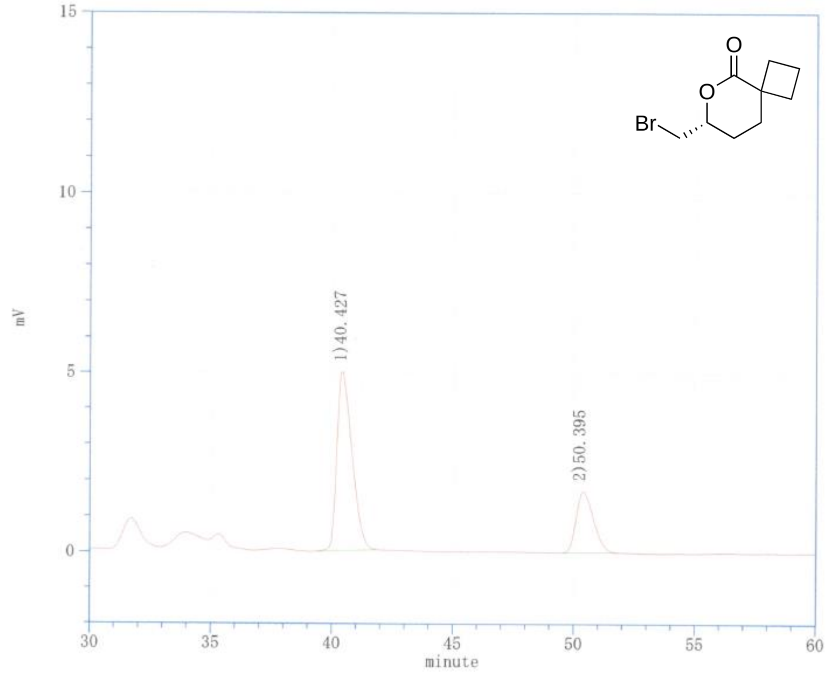


| | | |
|---|---|---|
| 40.427 | 216031 | 71.7456 |
| 50.395 | 85076 | 28.2544 |

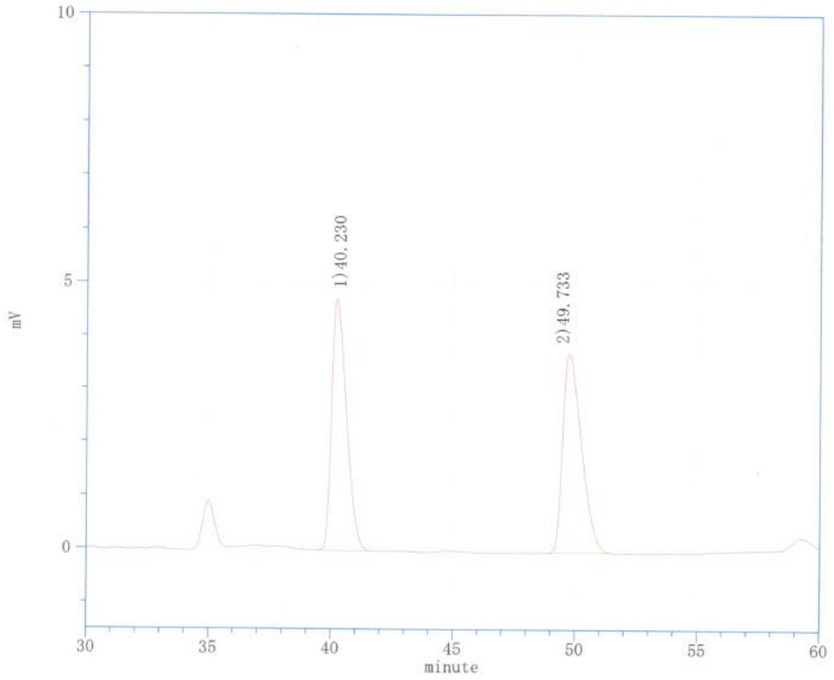


| | | |
|---|---|---|
| 40.230 | 198440 | 50.0708 |
| 49.733 | 197879 | 49.9292 |

**3h**: Daicel Chiralpak IE-3, hexane/2-propanol = 2:1, flow rate = 0.5 mL/min, 254 nm

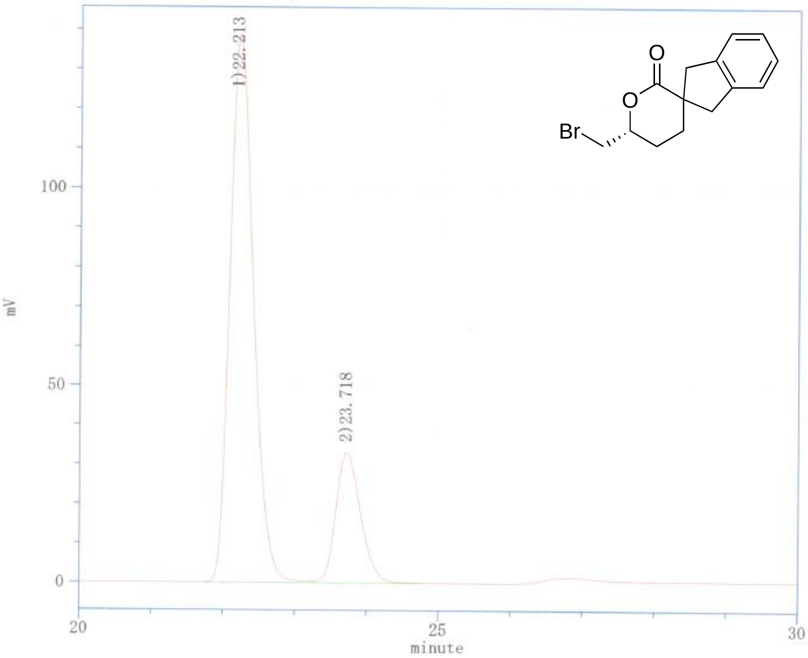


| | | |
|---|---|---|
| 22.213 | 3143495 | 80.0602 |
| 23.718 | 782921 | 19.9398 |

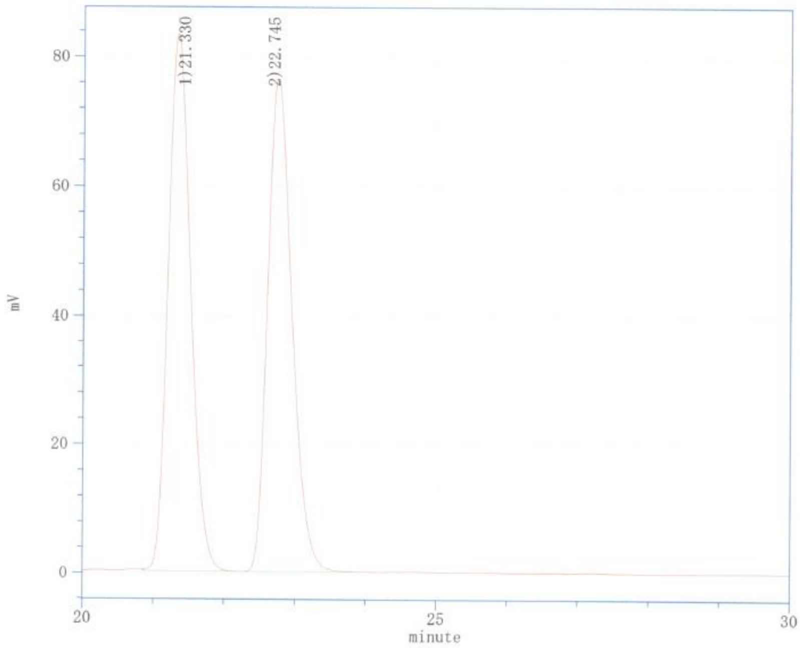


| | | |
|---|---|---|
| 21.330 | 1834694 | 50.1959 |
| 22.745 | 1820371 | 49.8041 |

**3i**: Daicel Chiralpak IE-3, hexane/2-propanol = 5:1, flow rate = 0.5 mL/min, 214 nm

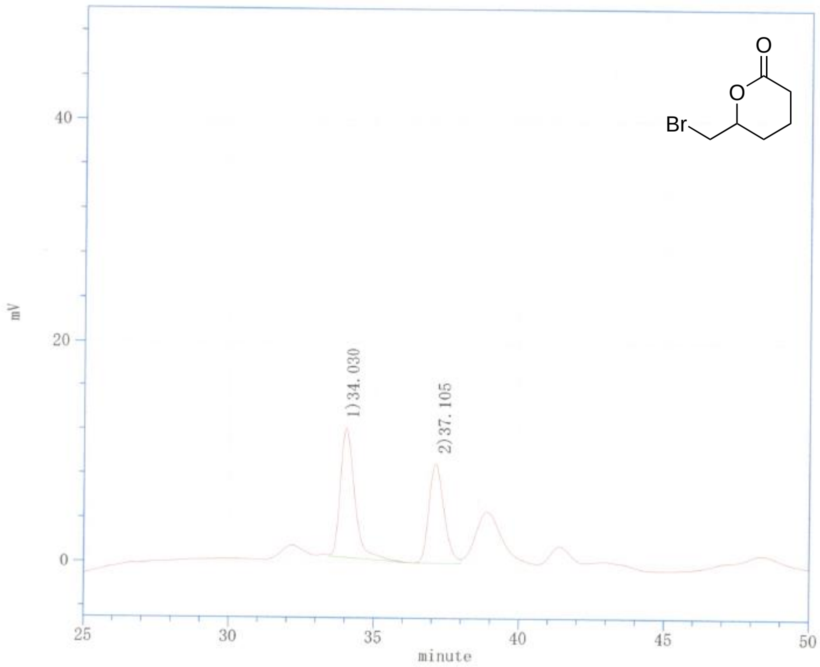


| | | |
|---|---|---|
| 34.030 | 402636 | 55.4373 |
| 37.105 | 323655 | 44.5627 |

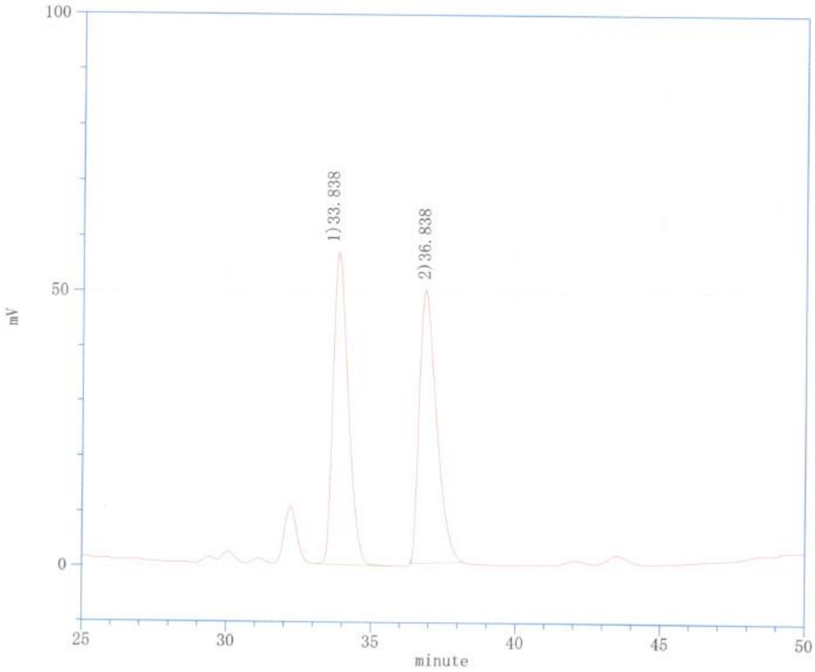


| | | |
|---|---|---|
| 33.838 | 2062228 | 50.0371 |
| 36.838 | 2059173 | 49.9629 |

**3j**: Daicel Chiralpak IE-3, hexane/2-propanol = 5:1, flow rate = 0.5 mL/min, 214 nm

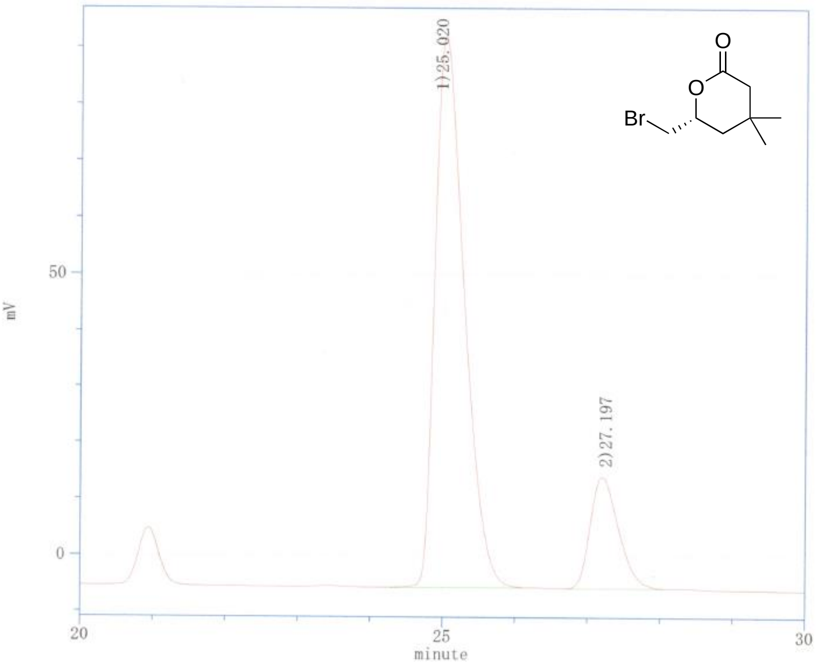


| | | |
|---|---|---|
| 25.020 | 2727226 | 83.6594 |
| 27.197 | 532689 | 16.3406 |

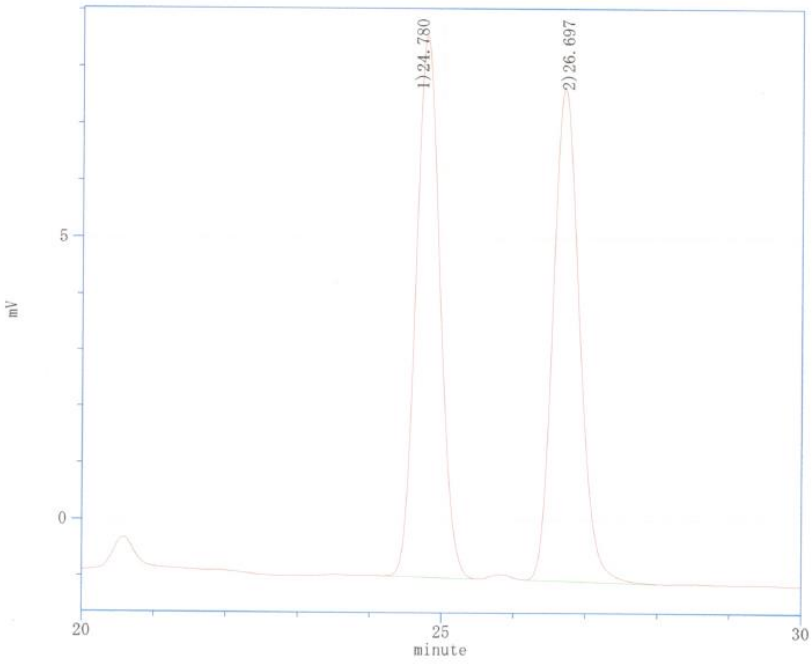


| | | |
|---|---|---|
| 24.780 | 221420 | 49.8743 |
| 26.697 | 222536 | 50.1257 |

**3k**: Daicel Chiralpak IG-3, hexane/2-propanol = 5:1, flow rate = 0.5 mL/min, 214 nm

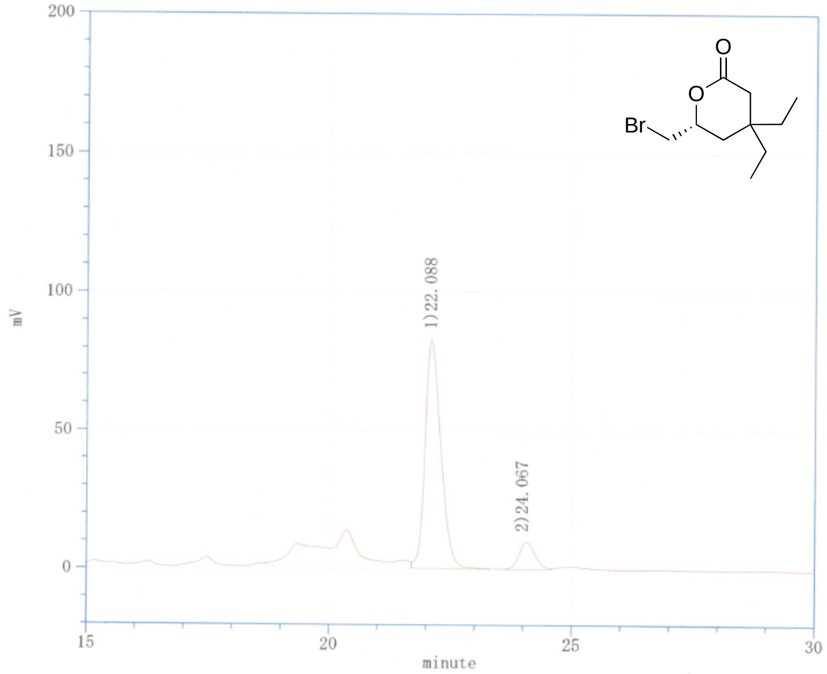


| | | |
|---|---|---|
| 22. 088 | 1861641 | 89. 1916 |
| 24. 067 | 225596 | 10. 8084 |

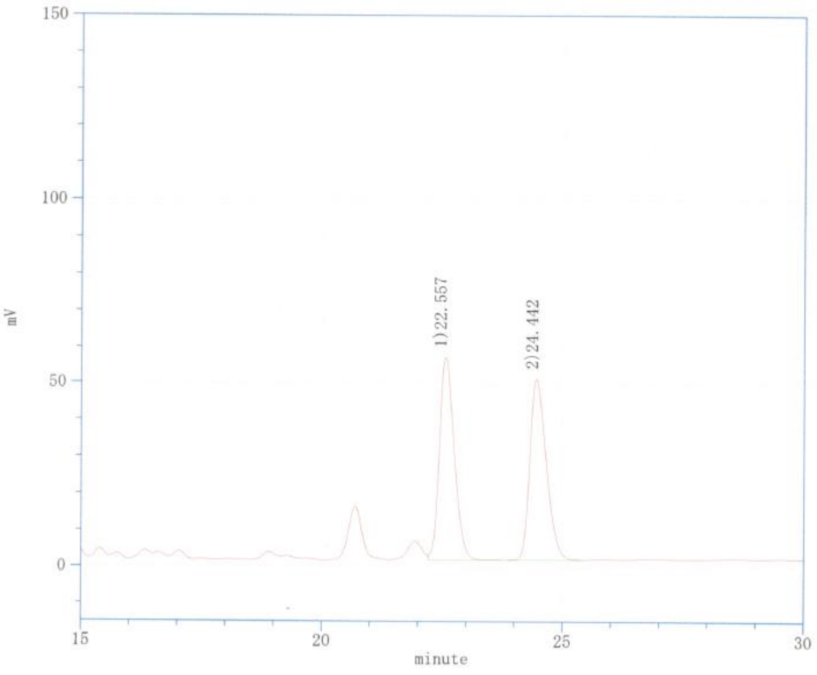


| | | |
|---|---|---|
| 22. 557 | 1182808 | 50. 2473 |
| 24. 442 | 1171165 | 49. 7527 |

**3l**: Daicel Chiralpak IE-3, hexane/2-propanol = 5:1, flow rate = 0.5 mL/min, 214 nm

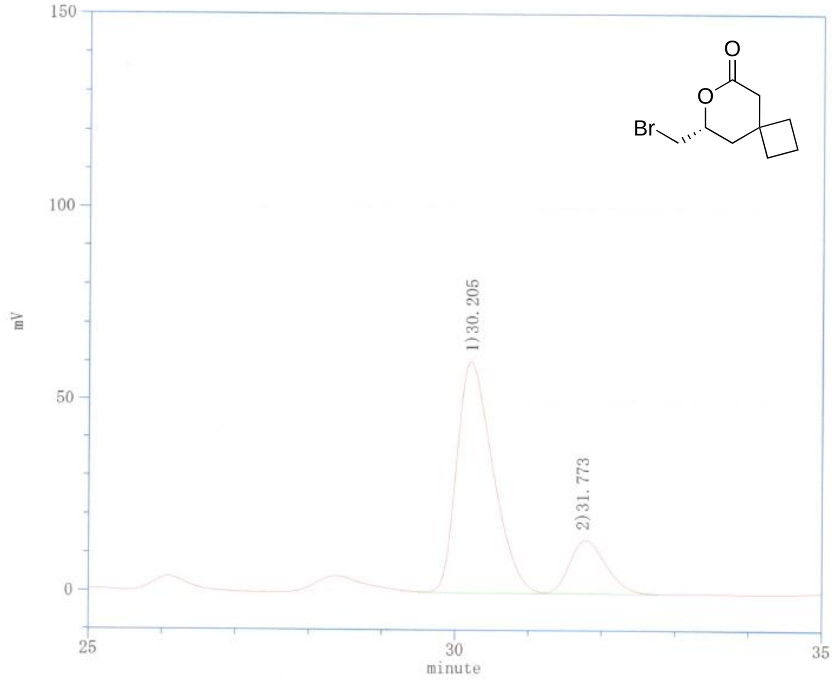


| | | |
|---|---|---|
| 30. 205 | 2065541 | 81. 2117 |
| 31. 773 | 477863 | 18. 7883 |

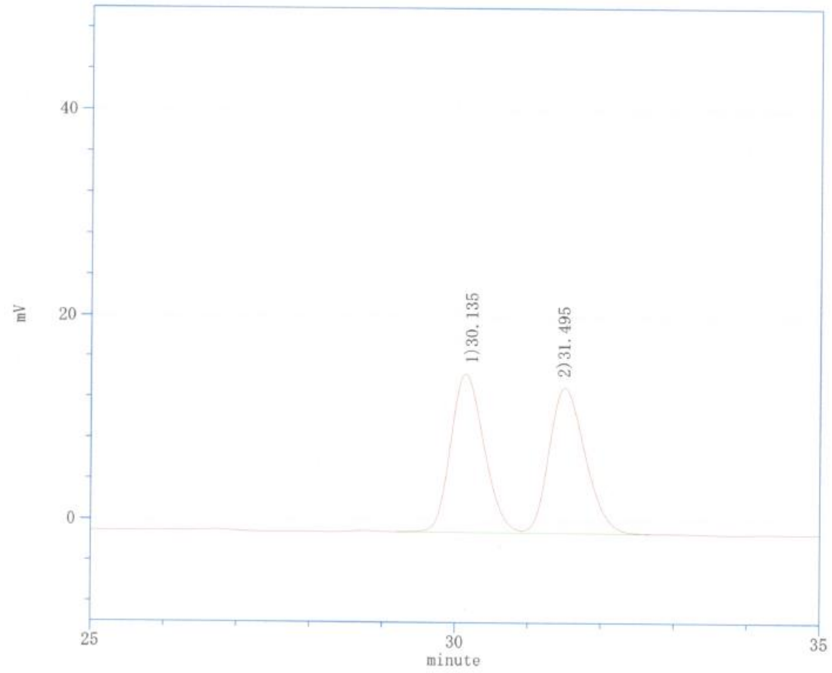


| | | |
|---|---|---|
| 30. 135 | 492944 | 50. 1758 |
| 31. 495 | 489489 | 49. 8242 |

**3m**: Daicel Chiralpak IG-3, hexane/2-propanol = 5:1, flow rate = 0.5 mL/min, 214 nm

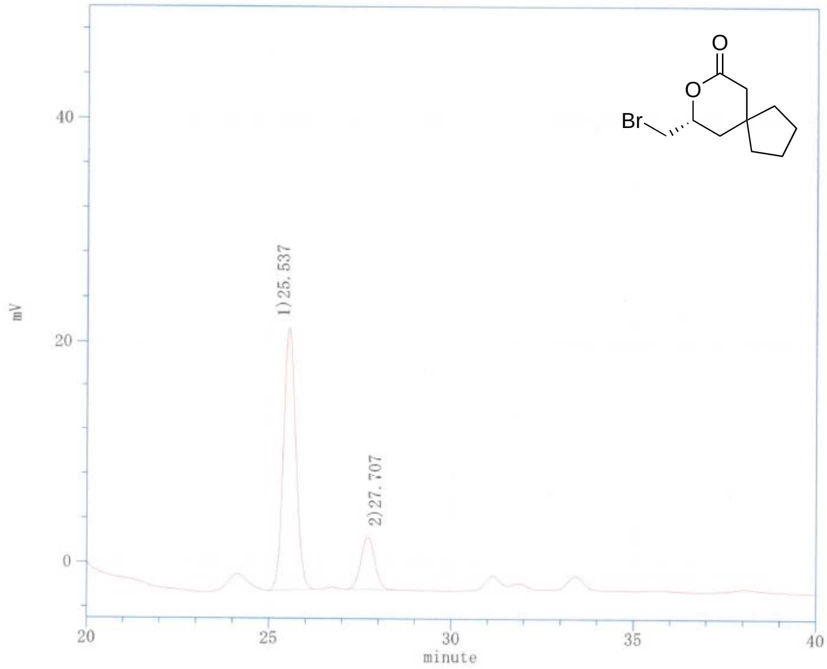


| | | |
|---|---|---|
| 25.537 | 558927 | 81.8608 |
| 27.707 | 123850 | 18.1392 |

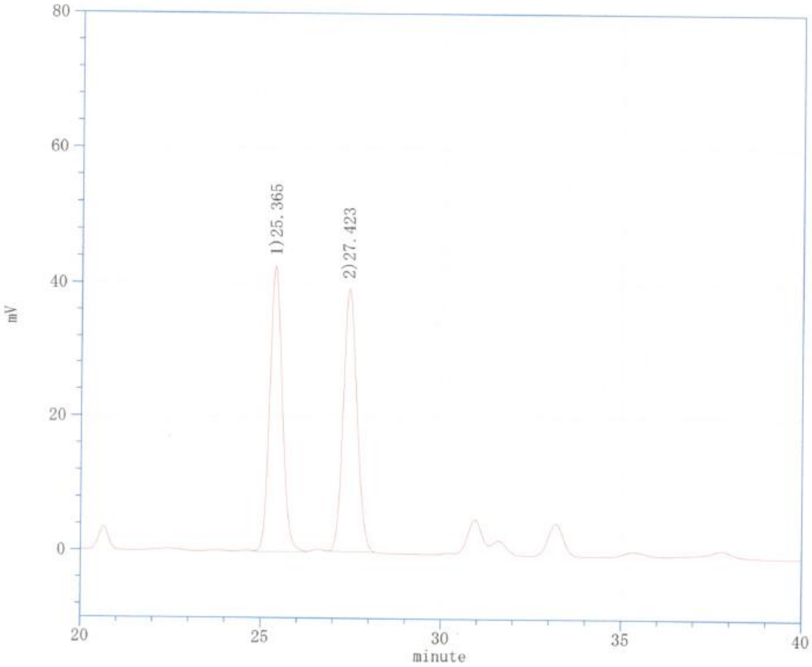


| | | |
|---|---|---|
| 25.365 | 1027807 | 49.8744 |
| 27.423 | 1032984 | 50.1256 |

**3n**: Daicel Chiralpak IG-3, hexane/2-propanol = 5:1, flow rate = 0.5 mL/min, 214 nm

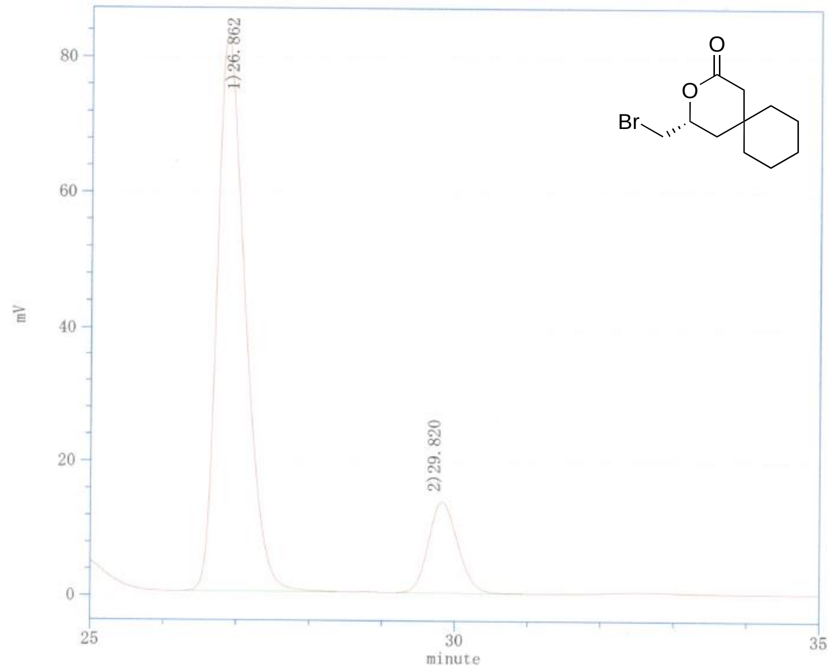


| | | |
|---|---|---|
| 26.862 | 2203906 | 85.3009 |
| 29.820 | 379778 | 14.6991 |

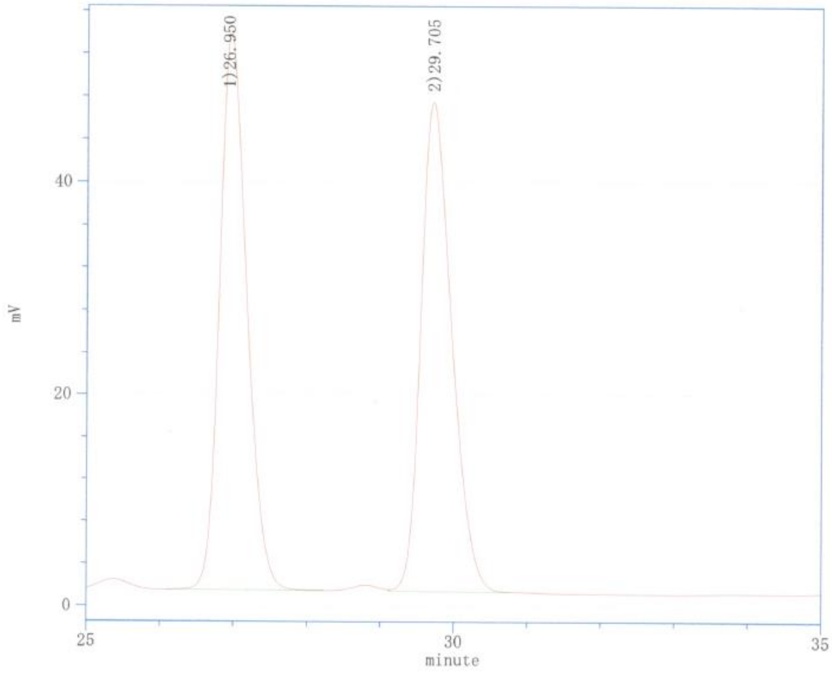


| | | |
|---|---|---|
| 26.950 | 1351054 | 50.1754 |
| 29.705 | 1341609 | 49.8246 |

**3o**: Daicel Chiralpak AS-3, hexane/2-propanol = 1:1, flow rate = 0.5 mL/min, 254 nm

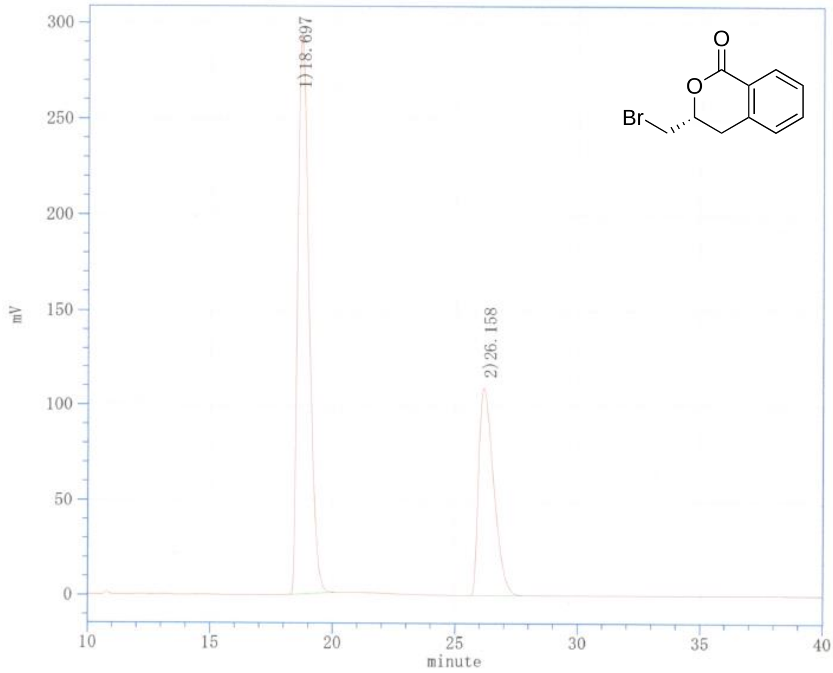


| | | |
|---|---|---|
| 18.697 | 9408720 | 66.9825 |
| 26.158 | 4637818 | 33.0175 |

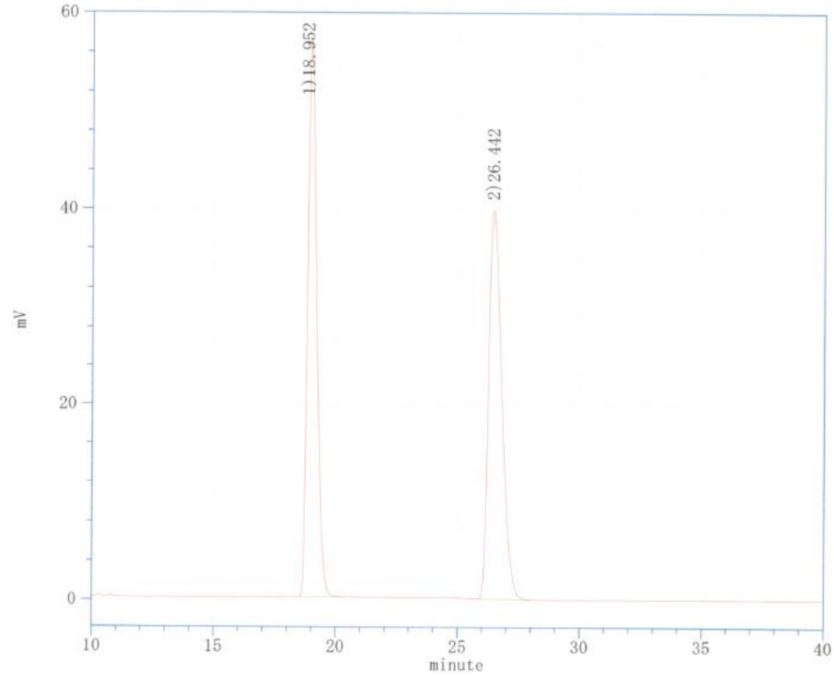


| | | |
|---|---|---|
| 18.952 | 1485652 | 49.9527 |
| 26.442 | 1488468 | 50.0473 |

**7**: Daicel Chiralpak IE-3, hexane/2-propanol = 2:1, flow rate = 0.5 mL/min, 254 nm

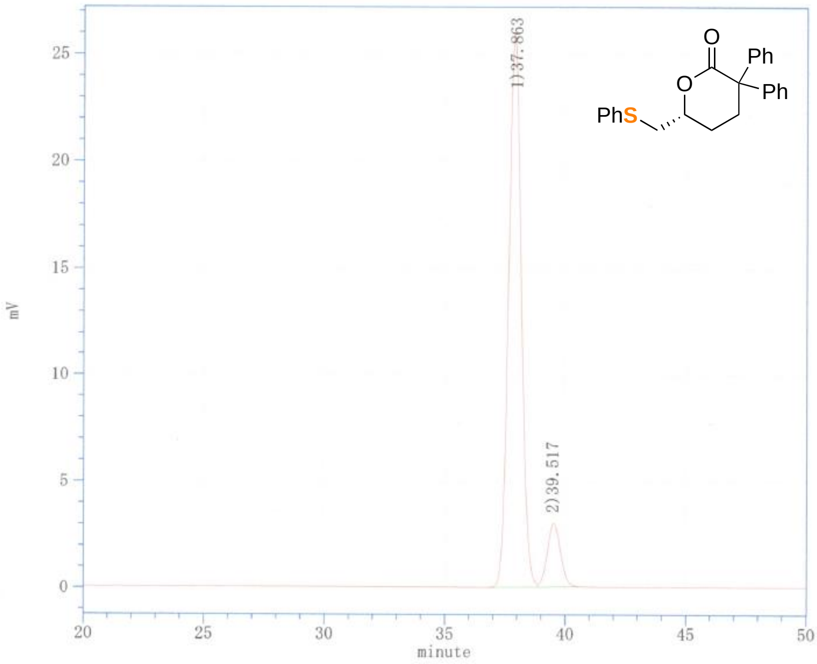

| | | |
|---|---|---|
| 37.863 | 947658 | 89.2704 |
| 39.517 | 113901 | 10.7296 |

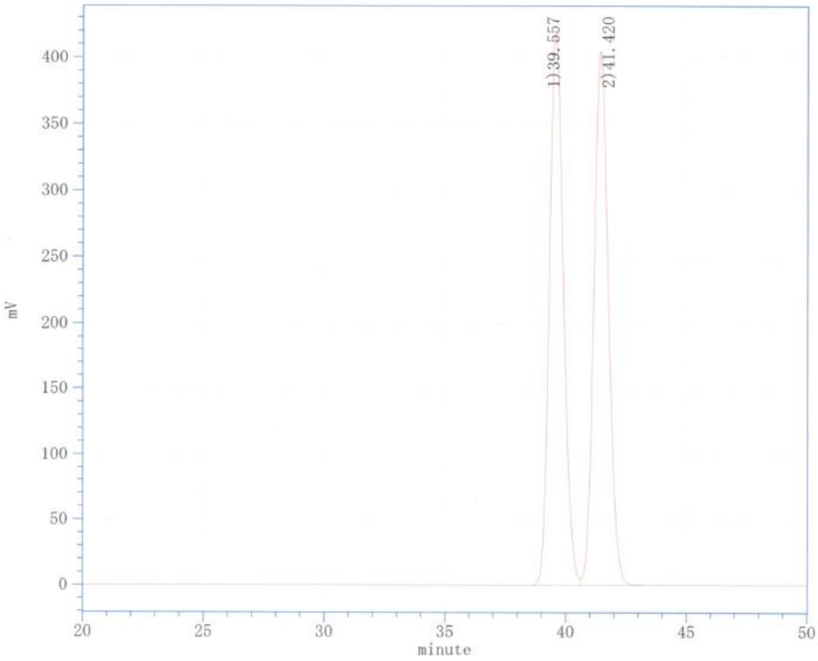

| | | |
|---|---|---|
| 39.557 | 16994030 | 50.0661 |
| 41.420 | 16949160 | 49.9339 |

**8**: Daicel Chiralpak IE-3, hexane/2-propanol = 2:1, flow rate = 0.5 mL/min, 254 nm

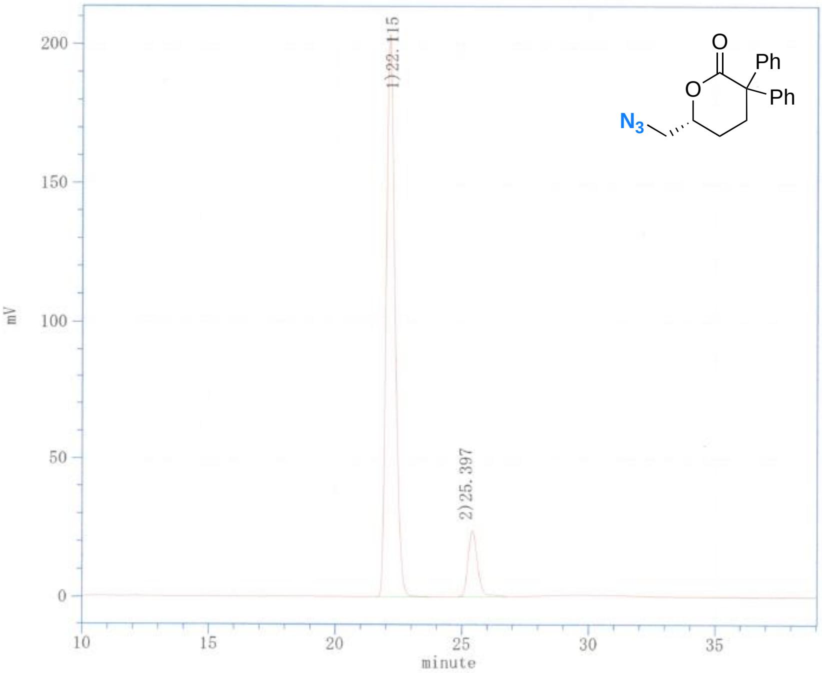


| | | |
|---|---|---|
| 22.115 | 4903154 | 88.7473 |
| 25.397 | 621694 | 11.2527 |

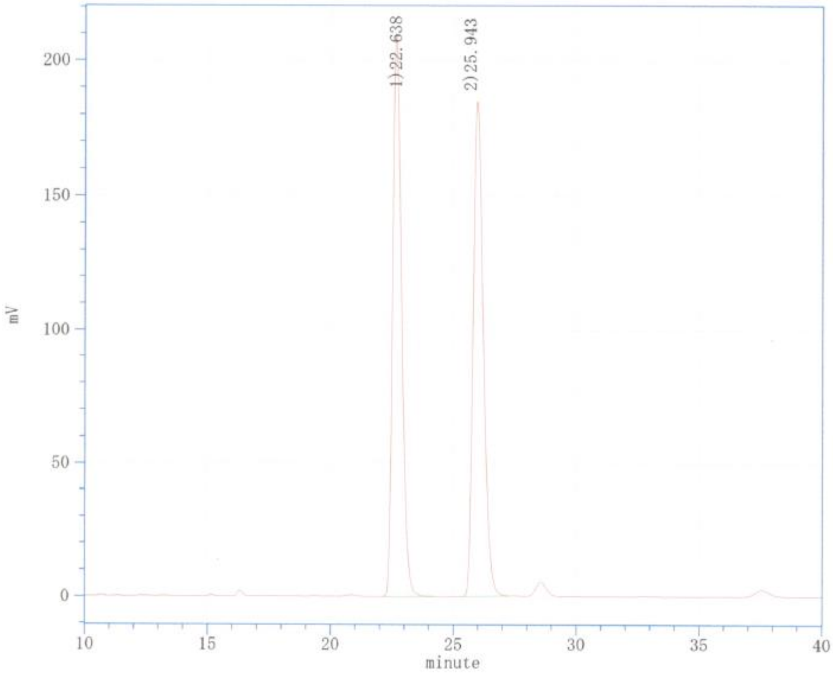


| | | |
|---|---|---|
| 22.638 | 5332079 | 50.0280 |
| 25.943 | 5326117 | 49.9720 |

**9**: Daicel Chiralpak IB-3, hexane/2-propanol = 20:1, flow rate = 0.5 mL/min, 254 nm

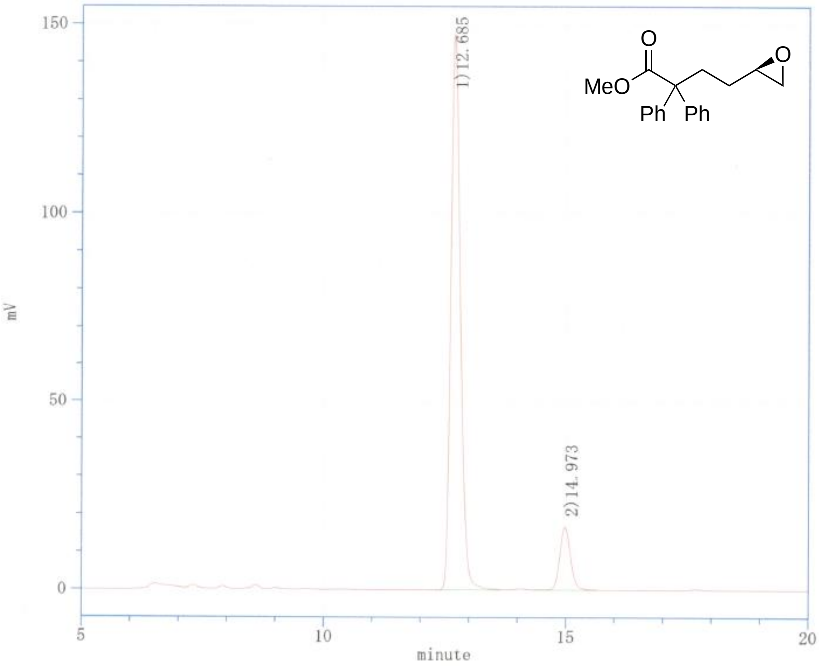


| | | |
|---|---|---|
| 12.685 | 2029262 | 88.8373 |
| 14.973 | 254984 | 11.1627 |

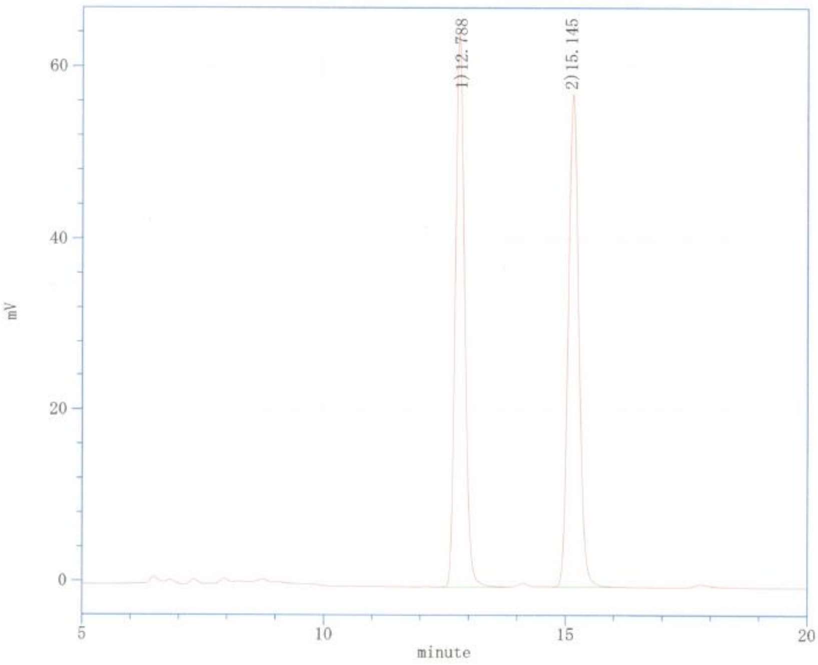


| | | |
|---|---|---|
| 12.788 | 862647 | 49.7922 |
| 15.145 | 869846 | 50.2078 |
